# Spatiotemporal changes in influenza A virus prevalence among wild waterfowl inhabiting the continental United States throughout the annual cycle

**DOI:** 10.1038/s41598-022-17396-5

**Published:** 2022-07-29

**Authors:** Cody M. Kent, Andrew M. Ramey, Joshua T. Ackerman, Justin Bahl, Sarah N. Bevins, Andrew S. Bowman, Walter M. Boyce, Carol J. Cardona, Michael L. Casazza, Troy D. Cline, Susan E. De La Cruz, Jeffrey S. Hall, Nichola J. Hill, Hon S. Ip, Scott Krauss, Jennifer M. Mullinax, Jacqueline M. Nolting, Magdalena Plancarte, Rebecca L. Poulson, Jonathan A. Runstadler, Richard D. Slemons, David E. Stallknecht, Jeffery D. Sullivan, John Y. Takekawa, Richard J. Webby, Robert G. Webster, Diann J. Prosser

**Affiliations:** 1 Department of Environmental Science and Technology, University of Maryland, College Park, MD USA; 2 U.S. Geological Survey, Eastern Ecological Science Center, Laurel, MD USA; 3 U.S. Geological Survey, Alaska Science Center, Anchorage, AK USA; 4 U.S. Geological Survey, Western Ecological Research Center, Dixon Field Station, Dixon, CA USA; 5 Department of Infectious Diseases, University of Georgia, Athens, GA USA; 6 U.S. Department of Agriculture, Wildlife Services, National Wildlife Research Center, Fort Collins, CO USA; 7 Department of Veterinary Preventive Medicine, The Ohio State University, Columbus, OH USA; 8 School of Veterinary Medicine, University of California Davis, Davis, CA USA; 9 Department of Veterinary and Biomedical Sciences, University of Minnesota, St. Paul, MN USA; 10 Department of Biological Sciences, California State University Chico, Chico, CA USA; 11 U.S. Geological Survey, San Francisco Bay Estuary Field Station, Western Ecological Research Center, Moffett Field, CA USA; 12 U.S. Geological Survey, National Wildlife Health Center, Madison, WI USA; 13 Department of Biology, University of Massachusetts, Boston, MA USA; 14 Department of Infectious Diseases, St. Jude Children’s Research Hospital, Memphis, TN USA; 15 Department of Population Health, University of Georgia, Athens, GA USA; 16 Department of Infectious Disease and Global Health, Tufts University, Medford, MA USA

**Keywords:** Zoology, Viral reservoirs, Influenza virus

## Abstract

Avian influenza viruses can pose serious risks to agricultural production, human health, and wildlife. An understanding of viruses in wild reservoir species across time and space is important to informing surveillance programs, risk models, and potential population impacts for vulnerable species. Although it is recognized that influenza A virus prevalence peaks in reservoir waterfowl in late summer through autumn, temporal and spatial variation across species has not been fully characterized. We combined two large influenza databases for North America and applied spatiotemporal models to explore patterns in prevalence throughout the annual cycle and across the continental United States for 30 waterfowl species. Peaks in prevalence in late summer through autumn were pronounced for dabbling ducks in the genera *Anas* and *Spatula*, but not *Mareca*. Spatially, areas of high prevalence appeared to be related to regional duck density, with highest predicted prevalence found across the upper Midwest during early fall, though further study is needed. We documented elevated prevalence in late winter and early spring, particularly in the Mississippi Alluvial Valley. Our results suggest that spatiotemporal variation in prevalence outside autumn staging areas may also represent a dynamic parameter to be considered in IAV ecology and associated risks.

## Introduction

Influenza A viruses (hereafter “IAV”), particularly highly pathogenic avian influenza viruses, pose a worldwide threat to the agricultural sector, public health, and some wild bird populations^1^. Of particular concern are the financial impacts of IAV to domestic poultry production associated with direct losses, culling and response efforts, and trade restrictions^2^. There may also be health risks associated with exposure and subsequent spillover of IAV from poultry into humans by direct contact with infected birds and virus-contaminated environments^3^. Finally, IAV can pose risks to some susceptible wild populations, when highly pathogenic viruses escape from poultry facilities^1^. Wild waterfowl act as the primary reservoir host of precursor viruses spread to poultry farms and these viruses can ultimately lead to poultry outbreaks^4^; the distribution and timing of which can be, at least partially, explained by wild duck movements^5^. Therefore, understanding spatiotemporal as well as taxonomic variation in the prevalence of IAV in wild waterfowl is useful for assessing risk posed to wild birds, the poultry industry, and human health^1,6,7^, developing effective response strategies^4^, and better understanding the viral ecology of this agriculturally important pathogen.

Extensive research has sought to understand temporal fluctuations in IAV prevalence in wild birds; however, sampling strategies may be hampered by temporal biases in field-collected samples. Numerous studies have shown that prevalence is greatest during the autumn as immunologically naïve juveniles become infected^8^. Comparatively less work exists outside of this season despite evidence that winter and spring may be important to the underlying viral ecology^9,10^. Additionally, past work has documented the potential for strong spatial variation^11^. Specifically, a broad seasonal latitudinal shift is well documented, with areas of comparatively high IAV prevalence shifting to northern latitudes as ducks migrate north during spring migration and occurring at more southern latitudes as ducks migrate back to the southern states during the winter^11,12^. Less work has examined longitudinal trends, though it is well established that viruses move among both global and North American flyways^13,14^. Although several studies have examined multiple waterfowl species, and it is generally supported both from field and host challenge studies that dabbling ducks are more susceptible to a wide diversity of IAV compared to other waterfowl taxa^15,16^, species-specific temporal variation in IAV prevalence remains understudied.

The primary objectives for this study were to better understand species-specific and spatiotemporal patterns of IAV prevalence. Here, we combined the two largest North American surveillance datasets, the U.S. Department of Agriculture (USDA), Animal and Plant Health Inspection Service (APHIS), National Wild Bird Avian Influenza Surveillance Program (hereafter “USDA”) and the National Institutes of Health (NIH), National Institute of Allergy and Infectious Diseases (NIAID) Influenza Research Database (hereafter “IRD”), to quantify taxonomic and spatiotemporal trends in IAV prevalence in wild waterfowl. In doing so, we seek to further our understanding of the broad risks posed by this virus, including to domestic poultry production and public health. Such information could be useful to managers seeking to optimize surveillance and improve biosecurity relative to the ecology of IAV in natural reservoir hosts.

## Results

**Figure 1:**
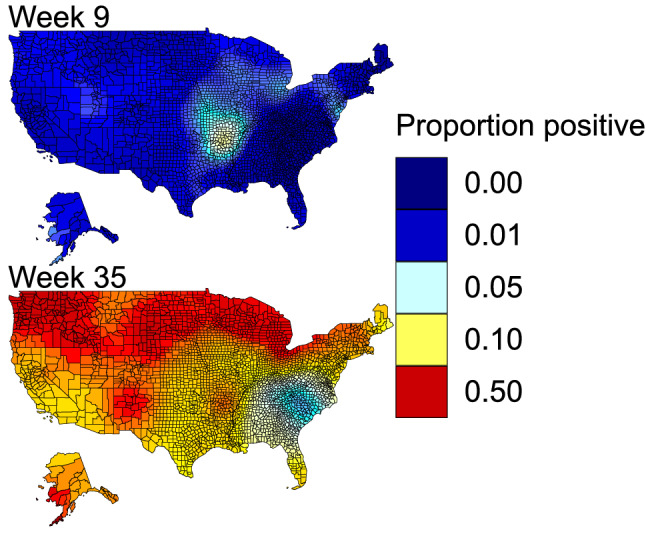
Example of spatiotemporal predictions of IAV prevalence in mallards. The listed weeks roughly correspond to the first week of March and September, highlighting the low levels of IAV prevalence in Spring outside of a relative hot-spot in the Mississippi Alluvial Valley, as well as the wide-spread elevated prevalence in fall across the northern latitudes. Maps were produced in ggplot2^68^. Full predictions for all species and week combinations are available in Supplementary Material 2.

Overall, 11.8% of all birds included in the analysis tested positive for IAV (see Table 1 for species summaries). Predicted proportion of birds testing positive for IAV for each species at weekly intervals for each county centroid in the continental United States are available at the U.S. Geological Survey (USGS) ScienceBase repository^17^ and are viewable in Supplementary Material 2. As an example, a subset of weekly spatiotemporal predictions for mallard are shown (Fig. 1). The model explained much of the variance in the training dataset (Supplementary Fig. S1 online, R^2^ = 0.58) and made reasonable predictions for the testing data (Spearman’s rho = 0.57, AUC = 0.77). We found a consistent pattern of elevated IAV detection in the USDA data compared to IRD and some modest variation by biological year (Table 2, Supplementary Fig. S2 online).

**Table 1:** Sample sizes for species included in the analysis from the IRD and USDA datasets and raw positivity rates not accounting for seasonal or spatial biases in sampling effort addressed in the provided predictions.

Common name	Scientific name	IRD	USDA	Total	% Positive
Snow Goose	*Anser caerulescens*	1518	5963	7481	6.17
Ross's Goose	*Anser rossii*	138	869	1007	2.98
Greater White-fronted Goose	*Anser albifrons*	821	674	1495	3.99
Brant	*Branta bernicla*	25	1843	1868	1.94
Cackling Goose	*Branta hutchinsii*	144	1507	1651	6.89
Canada Goose	*Branta canadensis*	630	20,279	20,909	2.37
*Mute Swan	*Cygnus buccinator*	1	2046	2047	3.57
Tundra Swan	*Cygnus columbianus*	12	1045	1057	4.82
Wood Duck	*Aix sponsa*	2958	26,213	29,171	3.21
Blue-winged Teal	*Spatula discors*	20,957	20,475	41,432	12.31
Cinnamon Teal	*Spatula cyanoptera*	334	1692	2026	19.30
Northern Shoveler	*Spatula clypeata*	6121	13,770	19,891	13.19
Gadwall	*Mareca strepera*	4306	18,565	22,871	4.35
American Wigeon	*Mareca americana*	4354	13,086	17,440	6.24
Mallard	*Anas platyrhynchos*	38,215	86,676	124,891	19.12
American Black Duck	*Anas rubripes*	727	4979	5706	15.98
Mottled Duck	*Anas fulvigula*	442	1717	2159	5.42
Northern Pintail	*Anas acuta*	12,290	18,165	30,455	13.04
Green-winged Teal	*Anas carolinensis*	10,787	35,530	46,317	11.60
Canvasback	*Aythya valisineria*	750	882	1632	3.19
Redhead	*Aythya americana*	664	2625	3289	4.59
Ring-necked Duck	*Aythya collaris*	1330	4085	5415	4.62
Greater Scaup	*Aythya marila*	324	795	1119	6.21
Lesser Scaup	*Aythya affinis*	2506	2678	5184	3.62
Common Eider	*Somateria mollissima*	1249	616	1865	1.23
Long-tailed Duck	*Clangula hyemalis*	1039	274	1313	2.58
Bufflehead	*Bucephala albeola*	869	2840	3709	5.55
Common Goldeneye	*Bucephala clangula*	681	778	1459	7.53
Hooded Merganser	*Lophodytes cucullatus*	167	866	1033	2.13
Ruddy Duck	*Oxyura jamaicensis*	210	697	907	5.73

*Mute Swan is non-native and non-migratory.

**Table 2:** Untransformed model effects giving the mean, standard deviation (Sd) and lower and upper 95% credible intervals of the posterior.

Effect	Mean	Sd	Lower 95%	Upper 95%
Intercept	− 4.686	0.326	− 5.326	− 4.047
Dataset	1.057	0.032	0.996	1.119
Precision for year	13.133	1.776	9.941	16.911
Precision for week	1.100	0.127	0.863	1.394
Correlation between weeks	0.840	0.013	0.813	0.865
Among-species correlation	0.247	0.060	0.136	0.383
Precision for phylogenetic effect	50.140	11.891	30.196	79.131
Precision for county	1.234	0.055	1.127	1.345
Correlation between months for counties	0.247	0.025	0.199	0.296
Range for spatial field	0.214	0.016	0.183	0.249
Sd for spatial field	0.970	0.052	0.872	1.078
Correlation between months for spatial field	0.773	0.015	0.743	0.802

**Figure 2:**
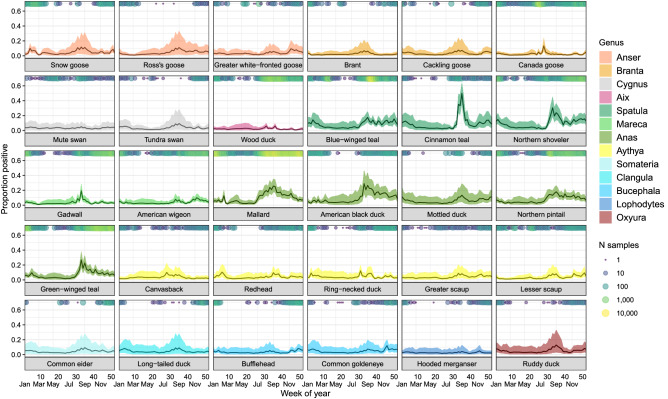
Percentage of individuals predicted to test positive for IAV (± 95% CI) for each species for each week. Predictions are based on the overall prevalence values in the USDA dataset, which had a higher detection rate, and ignore the spatial component. Circles running along the x-axis indicate the number of samples for each species taken during that week. Estimates for time periods without samples for a given species should be taken with caution as predictions are primarily based on the among-species correlation. Detailed images for each species can be found in Supplementary Material 3.

We found the temporal effects among species to be significantly correlated (Table 2), though distinct patterns were also identified (Fig. 2). Overall, most species show an increase in IAV positivity beginning in late June (~ week 25). For dabbling ducks in the genus *Spatula* (e.g., northern shoveler) and *Anas* (e.g., mallard), this peak reaches a maximum in late summer or early autumn (~ weeks 30–35) before declining into the winter. However, dabblers in the genus *Mareca* (gadwall and American wigeon) exhibited lower peaks in prevalence more comparable to other taxa (e.g., geese and diving ducks). In addition, we inferred a second, albeit generally lower, peak in prevalence during spring in many species (particularly evident in mallard and snow goose), generally occurring around February or March (~ weeks 5–10).

**Figure 3:**
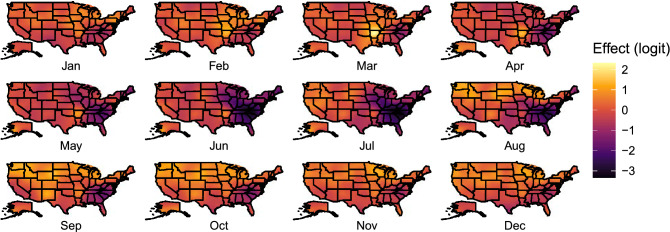
Monthly realizations of the spatial random field for the continental United States. Brighter colors indicate locations within a month with relatively greater IAV prevalence. Maps were produced in ggplot2^68^. Maps of the standard deviation can be found in Supplementary Fig. S3 online.

We also document clear spatial variation in IAV prevalence varying by month (Fig. 3, Supplementary Fig. S3 online). Spatially, we found evidence for elevated levels of IAV prevalence across the north-central United States, especially around the Prairie Pothole Region, starting in August and continuing through October. During autumn, areas of elevated prevalence shifted southward as well as into the Great Lakes and Pacific Northwest. Our results also supported elevated prevalence among birds in the Mississippi Alluvial Valley in the spring, particularly in March. Regardless of time of year, we found evidence for the lowest levels of IAV prevalence among wild birds in the southeast, where prevalence appeared to reach a minimum during summer months.

## Discussion

In this investigation, we provide a spatiotemporal model of IAV prevalence for 30 waterfowl species across the annual cycle and for the continental United States using two of the most comprehensive datasets available. These findings improve our understanding of variation in IAV prevalence, which may broadly be useful in assessing risks posed to wildlife, domestic poultry, and public health. This information is also useful in helping to inform management practices aiming to mitigate IAV outbreaks in the United States. Specifically, we confirmed the highest IAV prevalence in many dabbling duck species in the autumn, consistent with prior research^15,18–20^. We also elucidate broad temporal trends in prevalence. Moreover, we found evidence for additional time periods (e.g., spring) and geographic areas (e.g., the Mississippi Alluvial Valley) with increased IAV prevalence, which although lower than the autumn levels, may nevertheless play an important role in maintaining endemic circulation outside the major post-breeding pulse in North America.

We found greater IAV prevalence in the USDA dataset compared to IRD, presumably driven by methodological variation. This difference may be caused by the type of laboratory screening method that is used to determine an IAV positive result or possibly by field methods used when capturing waterfowl. Of these possibilities, the most likely may be the difference in detection for real-time reverse transcriptase PCR (rRT-PCR) as compared to virus isolation (VI) in embryonated chicken eggs, which reflects methodological differences influenced by the sampling objectives for agencies and institutions contributing to the USDA^21,22^ and IRD^23^ datasets. While all positive cases in the USDA dataset represent samples that tested positive with rRT-PCR^11^, positive cases in the IRD dataset may be the result of various methods depending on individual laboratories, sometimes first applying rRT-PCR and sometimes only applying VI, for which the number of positive detections may be lower as this method targets only viable viruses^24^. Unfortunately, while the type of test used to assess positivity may be submitted to the system, the final publicly available IRD dataset does not allow access to this information, preventing inclusion in our model. It is also possible that some other methodological differences are at play, such as trapping techniques (e.g., hunter harvest vs. baited live captures). That is, sampling of hunter-harvested birds or the capture of individuals via night-lighting may result in lower and less biased prevalence estimates as compared to birds congregating at bait, which can facilitate transmission among birds with increased contact rates^25^. However, data on sampling technique are generally missing from both datasets and would be useful to include in future efforts.

Regardless of the cause of these differences, the IRD and USDA datasets show similar spatial, temporal, and taxonomic trends, with analysis of model residuals showing little evidence of an interaction between dataset and any other explanatory variable (Supplementary Figs. S4, S5 and S6 online). As such, any difference between the two datasets may be one of detection probabilities rather than one that would shape our understanding of the underlying dynamics and thus we have merged these two datasets into one model. That is, both datasets show the same underlying trends, just with prevalence estimates from the USDA dataset being consistently higher than IRD. As differences may be primarily methodological, and the consistency of the USDA dataset leads to greater reproducibility, the predicted values provided in the main manuscript include the coefficient for the USDA dataset. We have also included predicted values using (a) the coefficient for the IRD dataset and (b) using the averaged coefficients for the IRD and USDA datasets in the data release^17^.

We found seasonal variation both among and within species. Overall, most species showed an increase in IAV prevalence in the autumn, a pattern that is well established and consistent with past work^15,18–20^. This pattern is presumably caused by high infection rates among recently fledged, immune-naïve juvenile birds entering the population in late summer and fall^8,26^. We then see decreased IAV prevalence through the winter as birds, especially juveniles, gain immunity^8^. This pattern is most pronounced in two genera of dabbling ducks (subfamily Anatinae), *Anas* and *Spatula*, where the peak in IAV prevalence is higher and lasts over a longer period than other species. Past work has shown elevated risk and prevalence of IAV in dabbling ducks, and this pattern has been linked to differences in foraging behavior^15,27,28^ but could also be caused by other differences in exposure or immune response^16,29,30^. Notably, and potentially evidence against the foraging behavior hypothesis, this pattern was not observed in both *Mareca* species, which are imbedded between *Anas* and *Spatula* within the larger dabbling-duck phylogeny^31,32^, and is consistent with past findings of lower prevalence in gadwall^33,34^. As such, we recommend additional research of IAV prevalence, exposure, and immune response among birds of different genera within Anatinae to uncover drivers of infection patterns.

Our results also provide evidence for a smaller peak of increased IAV prevalence for many species in late winter and early spring, typically between January and April, although the exact timing varies by species. Most past work notes only an autumn increase in prevalence^11^, with low levels of infection persisting year-round^10,20^. However, surveillance during February in a single Texas county in 2001–2002 did document an elevated prevalence of IAV^35^ consistent with our findings. There are several potential causes for this spring increase, including changes in circulating subtypes^9,12^ to which ducks may lack immunity^36^ or a decline in the strength of immune response after the autumn peak^37^. Alternatively, this pattern may be linked to spring migration either due to physical stress depressing the immune response or large congregations of ducks at staging areas increasing transmission; an explanation further bolstered by recent findings of modestly elevated prevalence rates in migrating teal along the Gulf Coast^38^.

Though we did not find clear differences in IAV prevalence among flyways, with areas of high and low prevalence within each, we did find spatiotemporal variation. Although not tested here, these spatiotemporal trends may be related to regional duck densities and distributions. From August through October, during the period of the highest IAV prevalence, we see increased infections across the north-central United States. This is generally consistent with the areas of the greatest duck population densities within the United States during early autumn^39^. As autumn progresses, we see these areas of highest prevalence shift to the south, presumably with duck migration, as well as into the Pacific Northwest and Great Lakes regions where large populations of ducks congregate^40^. As such, it appears that patterns in IAV prevalence, at least at large spatial scales, may be related to duck density, which presumably increases viral transmission rates^41^. However, testing this specifically will require further work, especially to elucidate if the distributions of specific species drive this overall spatial trend.

**Figure 4:**
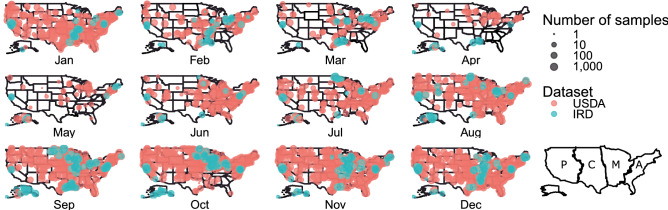
Sampling effort by month for both the USDA national surveillance program and the NIAID Influenza Research Database (IRD) datasets and map of the waterfowl flyways. USDA provides greater coverage across the continental United States as sampling was stratified by flyway and watershed. The majority of IRD sampling events are located either in the Mississippi (M) and Central (C) Migratory Flyways, California and Alaska in the Pacific (P) Migratory Flyway, and Maine and Delaware in the Atlantic (A) Flyway. However, IRD adds greatly to the total sample coverage during the spring lull in sampling effort. Maps were produced in ggplot2^68^.

Interestingly, though reported sampling efforts and overall IAV infections are generally low during the spring, we also see recurring elevated prevalence in the Mississippi Alluvial Valley in Arkansas during this period. The exact cause of this is unclear, however we offer several potential hypotheses that may explain this observation. First, this is a region of particularly high densities of wintering ducks, especially mallards^42^, though this peak occurs in March after many of these birds have departed for spring migration^43^. It is possible that viruses shed into the water from the previous season persist^44,45^, causing infections in the remaining birds and/or spring migrants arriving from farther south, especially as IAV presence in sediment is closely related to waterfowl density^46^. The arrival of spring migrants may also be associated with the dispersal of virus strains northward from wintering grounds in the Neotropics, potentially exposing birds in this region to strains for which they lack immunity^5,12,47^. Regardless of the ultimate cause, this peak in viral prevalence within the Mississippi Alluvial Valley may play some role in maintaining viral diversity in the United States^10,26,48^. Moreover, as the geographic coverage of sampling during this period is limited (Fig. 4), increased geographic coverage may shed light on other potential areas of elevated prevalence in spring.

Large amounts of the data in both datasets were not designed to assess prevalence. The USDA data were originally collected for early detection of potentially highly pathogenic viruses of the H5 and H7 subtypes^21,22^. In contrast, though the overarching goal of the IRD dataset is to forecast pandemic potential and effort was spent on all 16 HA subtypes, the specific goals behind data collection varied by research group^23^. In general, large sections of both datasets were collected to maximize the number of detections, creating biases related to when and where samples were collected and generating temporal and spatial autocorrelation that if ignored would lead to an over estimation of IAV prevalence. We dealt with these biases within the model by including two spatiotemporal components. To handle biases at large spatial scales as well as to assess how prevalence varies across time and space, we included a spatiotemporal stochastic partial differential equation (SPDE) model based on a continuous Gaussian random field. To handle smaller scale spatial variation, such as specific choices of locations to sample, we included an effect of county, accounting for a lack of independence for birds sampled from the same locations. The inclusion of county prevented the SPDE component from overfitting the data and greatly improved overall model fit. Temporal biases in data collection are also confounded with potential species differences, with some species lacking sampling data from certain time periods. As species are likely not fully independent from each other, we included both a phylogenetic random effect and a correlated temporal species effect^49^, which is able to pull statistical power and information for a species that is poorly sampled at a given time from other species with more samples. In short, this method both improved model fit and allowed for justifiable predictions during periods of low sampling. There was also the potential for an interaction between bird species and the spatial components; however, this appears to not be the case as during model validation we found no evidence of significant residual spatial autocorrelation by species and thus excluded this from the model.

Despite our handling of spatiotemporal autocorrelations, it should be noted that there was generally less data, especially for some species, during the late winter and spring (Fig. 4). Consequently, spatial predictions are less informative during this time of year. Moreover, though our model assumes some correlation among species, it is possible that some of the under-sampled species may experience unique patterns during periods when data are missing (Fig. 2). As such, we would recommend caution when making inferences for species during times that they were not sampled. Moreover, as we document both a spring resurgence in several species and relatively high IAV rates in the Mississippi Alluvial Valley during this time, our results provide rationale for increased winter and spring sampling, especially in areas of high duck density, to both monitor outbreaks and better understand viral ecology.

Additionally, several weaknesses exist from potentially important missing variables that we were unable to include in the model. First, though we know susceptibility to IAV varies by age, with immuno-naïve juveniles being more susceptible^8^, we lacked data on the age as well as sex for most birds. Secondly, we lack detailed data on trapping and collection methods. Many birds in both datasets were sampled from hunter harvest, while others were collected through techniques using baited sites, such as swim-in traps or rocket netting^11,23^. The elevated local duck densities caused by congregations of birds around bait stations may have artificially increased rates of IAV transmission when birds were collected with these methods^25^. As such, it is possible that we overestimated IAV prevalence in some places, particularly outside of hunting seasons. Notably, this does not appear to be the case for the samples from the Mississippi Alluvial Valley in spring. Though some birds may have been baited, the reported numbers captured at a given time were small, indicating that densities were not artificially increased from this method, and we also see an increased prevalence in some known hunter-harvested birds in this time and place.

In conclusion, our modelling efforts confirmed previous reports of waterfowl species, times of year, and geographic areas with elevated IAV prevalence that may be useful in future efforts aiming to identify areas of probable viral spillover from wild birds to poultry and to ultimately mitigate the risks of IAV outbreaks among domestic birds and human populations. In addition to supporting a peak in IAV infections during the late summer and early autumn in many dabbling duck species, we also found evidence for a secondary, smaller peak in IAV infections during the spring. Furthermore, we identified potentially important geographic areas of increased prevalence during the annual cycle. As such, future research on IAV in waterfowl would benefit from including sampling throughout the full annual cycle to confirm the inferences derived from this study, as time periods outside the well-established autumn peak may be important for understanding overall viral ecology. Moreover, the underlying drivers of many of the patterns we identify remain unresolved, and future research may be helpful for clarifying their behavioral, physiological, and evolutionary origins.

## Methods

### Datasets

Data on IAV prevalence came from two national-scale surveillance datasets. The first of these is from the U.S. Department of Agriculture (USDA), Animal and Plant Health Inspection Service (APHIS), National Wild Bird Surveillance Program^11,21^, and sought to maximize detections of positive birds for early detection of pathogenic strains. Sampling, stratified by administrative flyways and species, was conducted from 2007–2011 and 2015–2019. Birds were tested with both an oropharyngeal and a cloacal swab, which were added to a single cryovial containing brain–heart infusion media. Samples were tested at National Animal Health Laboratory Network facilities for IAV by rRT-PCR^50,51^.

The second dataset is the National Institutes of Health (NIH), National Institute of Allergy and Infectious Diseases (NIAID) Influenza Research Database^23,52^ (IRD; www.flubd.org), which is a publicly available database containing IAV surveillance data from many different academic labs. We downloaded all waterfowl surveillance data on June 15, 2021 that was collected from 2007 to 2019. Labs that contributed data to IRD use a variety of screening methods to detect IAV in samples, including rRT-PCR and VI. Protocols for collecting swabs also varied among labs that deposited data. Most birds were sampled with either cloacal and/or oropharyngeal swabs, though other sample types were also collected (e.g., fecal or tissue), with some birds sampled using multiple methods. We attempted to account for methodological variation by including the number of swabs taken from each bird as a variable in the model. However, we found that the effect was both negligible and insignificant, and it was removed from the final model.

After observing similar trends in the raw data for both the IRD and USDA datasets (Supplementary Figs. S7, S8 and S9 online), we combined data from both databases for the continental United States. While developing models to include Canada was desired, the long-term data set did not have spatial and temporal coverage sufficient for creating robust predictions at our current model scale and thus were not included. Taxonomically, we included all Anseriformes species with more than 550 individuals sampled – excluding hybrids, captives, or domestics – leaving 30 species. In total, this included 292,230 and 114,569 samples from the USDA and IRD datasets respectively. Due to variation in spatial precision within the two datasets, we aggregated all data to county using latitude and longitude coordinates and TIGER/Line files from the U.S. Census (www.census.gov/geographies/mapping-files/time-series/geo/tiger-line-file.html), reassigning geographic coordinates based on county centroids (Fig. 1, Supplementary Figs. S10, S11 and S12 online).

To better reflect the annual cycle, we replaced calendar year with “biological year” commencing each June 1. Previous studies have used a cutoff date of April 1 to align with the start of mallard breeding in the Prairie Pothole Region^11,18,53^. However, our study considered a larger geographic area encompassing the continental United States. Moreover, in examining the raw data, we found that this date falls during a period of elevated prevalence in some species, including mallard. Instead, we chose to use a cutoff of June 1, which is near the center of an extended period each year with low IAV prevalence, to prevent the year variable breaking in the middle of a period experiencing increased prevalence.

### Statistical analysis

To quantify the probability of a bird testing positive for IAV, we fit a Bayesian hierarchical spatiotemporal model with a correlated species-specific temporal latent effect using integrated nested Laplace approximation^54^ (INLA) with R-INLA version 20.10.12–1. This is a Bayesian approximation method for estimating latent Gaussian models that is computationally efficient, making it ideal for the analysis of large, spatiotemporal datasets. We pooled data by all prediction variables (dataset, biological year, month, week, county, species) to set up a binned-binomial regression model, with 25% of the binned data randomly withheld from model training as a testing dataset. This model estimates the probability of a bird testing positive for IAV (*y*) with a binomial likelihood and logit link^55^ that can be expressed as,

1 $$y\sim binomial\;\left( {n,\pi } \right)$$

2 $$logit\left( \pi \right) = \beta_{0} + \beta_{1} \cdot Dataset + \beta_{2} \cdot f_{y} Year + \beta_{3} \cdot f_{c,m} County + \omega_{l,m} + \psi_{w,s} + \varphi_{s}$$

where *β*_*0*_ is the intercept, *β*_*1*_ is a fixed effect for which dataset the data came from, and *β*_*2*_∙*f*_*y*_*Year* is an independent and identically distributed (iid) latent effect accounting for variation in IAV prevalence by biological year.

The model contained two spatiotemporal components designed to handle spatiotemporal autocorrelation at different spatial scales. The first, *β*_*3*_∙*f*_*c,m*_*County*, is an iid effect of county (*c*) that varies across months (*m*) based off of a cyclic first-order autoregressive (ar1) model^56^ to account for the cyclic nature of the annual cycle and to allow for a correlation between adjacent months. This effect is intended to handle small-scale spatial autocorrelation, such as that derived from differences in methods between labs or choices of specific sampling sites. Secondly, *ω*_*l,m*_ is a spatially structured latent effect of the correlation between location (*l*) and month (*m*) modeled as a cyclic ar1 model with the same temporal realizations using an SPDE approach^57^ and is intended to quantify large-scale spatial variation. The SPDE method allows for efficiently computing large spatial models and quantifies continuous spatial autocorrelations. It uses a continuous Gaussian random field constructed with a three-dimensional triangular mesh which had 8202 nodes, comparable to studies at similar spatial scales^39,58^, and was projected onto a three-dimensional sphere scaled to one Earth radius. The temporal effect of these spatial models is at the level of month instead of week to maintain sufficient data in each temporal realization to inform the spatial field.

The model also contained a correlated species-specific latent effect (*ψ*_*w,s*_) quantifying weekly (*w*) variation among species (*s*). The effect of week was included as a cyclic ar1 model, and was set up to generate a separate, but correlated, trend for each species. This was done as there were strong temporal biases in the data with many species missing data for certain parts of the year, leading to poor model predictions when we considered species as completely independent. However, due to similar physiology and phenology, as well as the ability to transmit IAV among each other^48^, we expected some level of correlation among species. To account for this, we used correlated stratum-specific smoothing priors^49^, which allow for better predictions when data is missing by pulling statistical power from other species based on the among-species correlation.

Additionally, as more closely related species may have more similar immune responses and physiology, we included a random phylogenetic effect (${\varphi }_{s}$) as a precision matrix. We downloaded 10,000 samples from the posterior distribution of the phylogenetic hypothesis available at BirdTree^59^ (www.birdtree.org) for all Anseriformes to generate an ultrametric consensus tree^60^. This tree was then used to generate a variance–covariance matrix using the package ape^61^ using a Brownian motion model, where the diagonal represents the root-to-tip distance and the off-diagonal elements represent the shared branch length between two species. This variance–covariance matrix was subset to only include the species considered here. The matrix was then standardized by dividing it by the determinant raised to the power of 1/N_species_^62^ and inverted into a precision matrix^63^. We also tested a model that allowed for an interaction between the phylogenetic effect and week of year; however, this model received less support (Δlog(Σ CPO) = 144), and was removed from further consideration.

We used penalized complexity (pc) priors for all correlation terms, which are robust priors designed to penalize the model for any deviations from a simpler base model in support of Occam’s razor. Pc priors for the spatial effect^64^ were specified so that the spatial range had a 0.5 probability of falling within one half the radius of the earth (*ρ*_*0*_ = 1, *p*_*ρ*_ = 0.5) and reasonable standard deviation (*σ*_*0*_ = 1, *α*_*σ*_ = 0.01), which was intended to be non-informative. All temporal autocorrelation terms as well as the among-species correlation used the same pc priors (*μ* = 0, *α* = 0.9) where the base model for the correlation is *ρ* = 1^56,65^. All other components used INLA’s default priors^54^.

### Model checking

Statistical inference was based on means and 95% credible intervals of the posterior distributions. We measured goodness of fit using a coefficient of determination (R^2^) based on a regression of the posterior predictive distribution against the observed values, weighted by the number of samples in the binned data (*n*)^58^. We performed out-of-sample cross validation by computing Spearman’s correlation, again weighted by *n*, between the training and test datasets using the package wCorr^66^ and used the package PresenceAbsence^67^ to calculate the area under the receiver operating characteristic (AUC) curve. Finally, we generated model predictions of the probability of a bird testing positive for IAV for each species at the county level in weekly intervals. These predictions were generated by averaging the year effect across all years, and we scaled the results to the overall prevalence in the USDA dataset because of the consistency of its methods, though values for IRD and the two datasets averaged together are provided in the data release. It should be noted that as all species shared a common spatial field, we did not choose to mask the geographic regions outside of species’ ranges, and thus predictions do exist for locations beyond a species’ natural range.

## Supplementary Information

Supplementary Information 1.

Supplementary Information 2.

Supplementary Information 3.

Supplementary Information 4.

## Data Availability

The datasets analyzed during the current study come from three sources. Phylogenetic data came from BirdTree and are publicly available at www.birdtree.org. Surveillance data from IRD are publicly available at the NIAID Influenza Research Database www.fludb.org. Surveillance data from the USDA are not publicly available as they are the property of the U.S. Department of Agriculture and are available from the corresponding author on reasonable request. All model predictions are available online^17^.
