# Supplementary material for: Spatiotemporal changes in influenza A virus prevalence among wild waterfowl inhabiting the continental United States throughout the annual cycle
Source: Sci Rep. 2022 Jul 29;12:13083. doi: 10.1038/s41598-022-17396-5 (PMC9338306; doi:10.1038/s41598-022-17396-5)
Supplement: Supplementary file 1 — Supplementary Information 1. [file 41598_2022_17396_MOESM1_ESM.docx]

Supplementary Material 1: Supplemental Figures


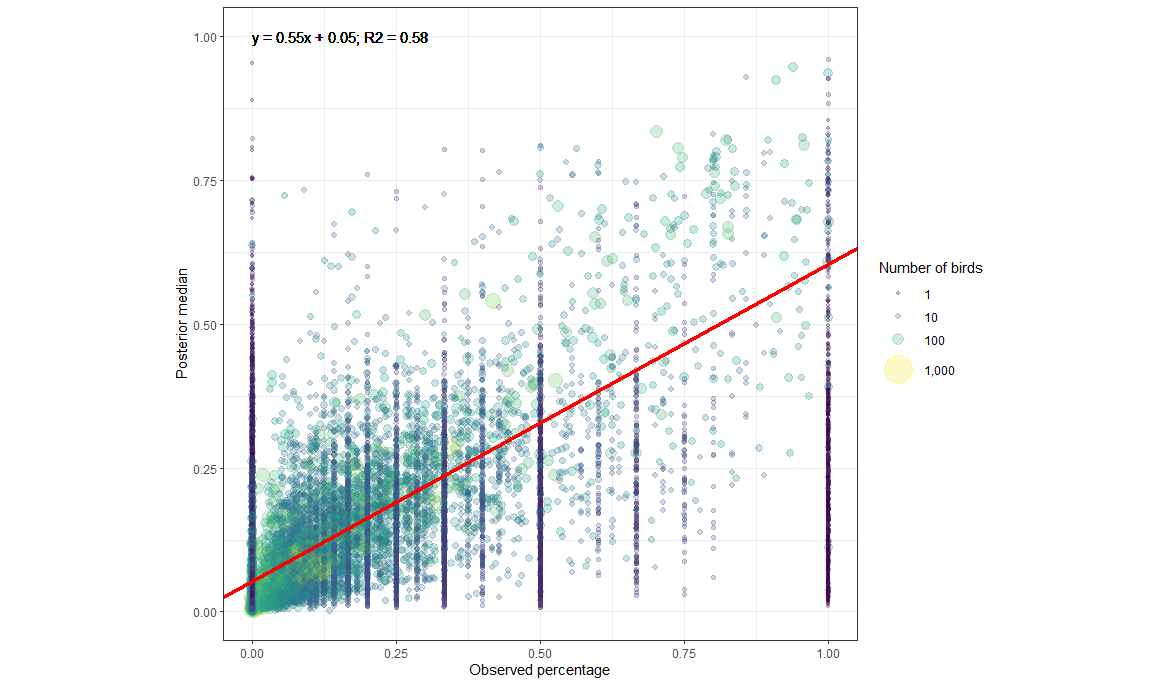


Supplementary Figure S1. Weighted regression of the posterior median against the observed percentage of IAV positive birds, weighted by the number of birds sampled (*n*).


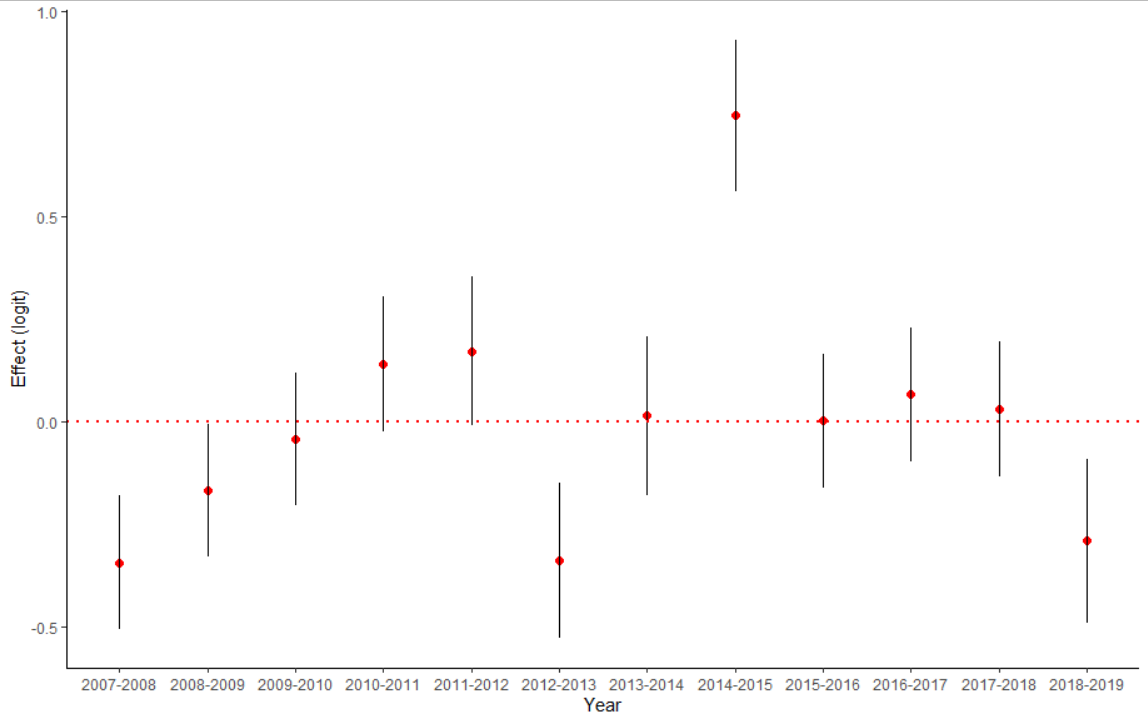


Supplementary Figure S2. Changes in prevalence estimates by biological year. The increased variance from 2012-2014 is likely due to a large decrease in the number of available samples during this period when the USDA was not conducting surveillance.


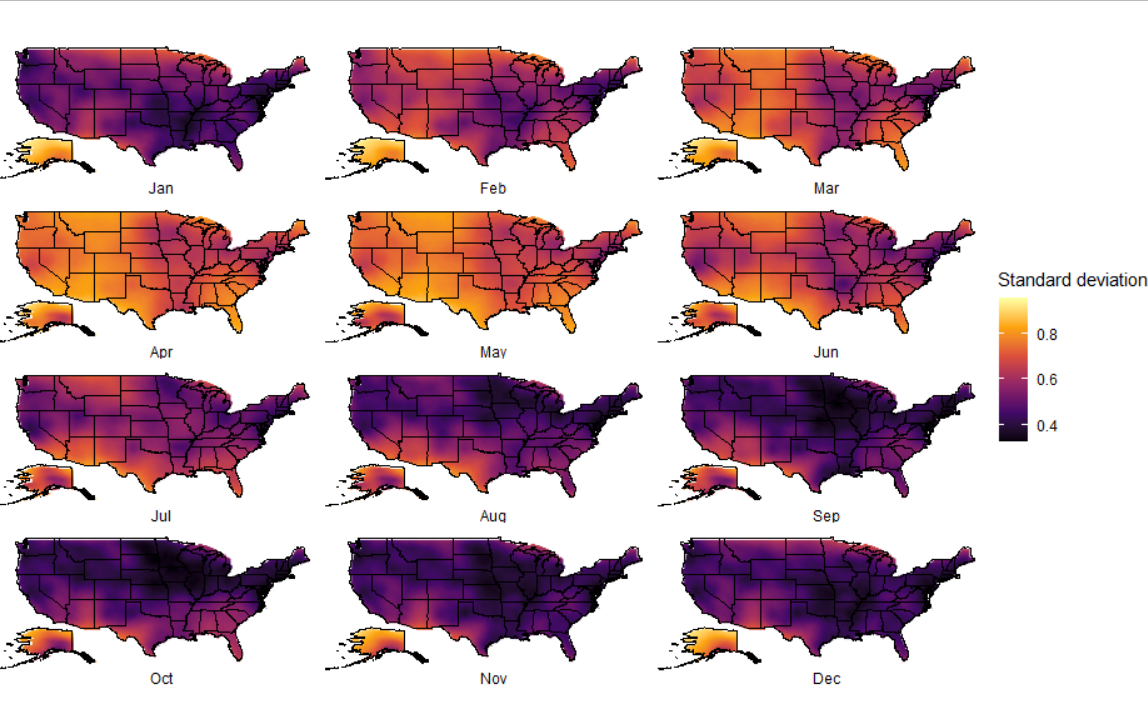


Supplementary Figure S3. Monthly realizations of the standard deviation for the spatial random field.


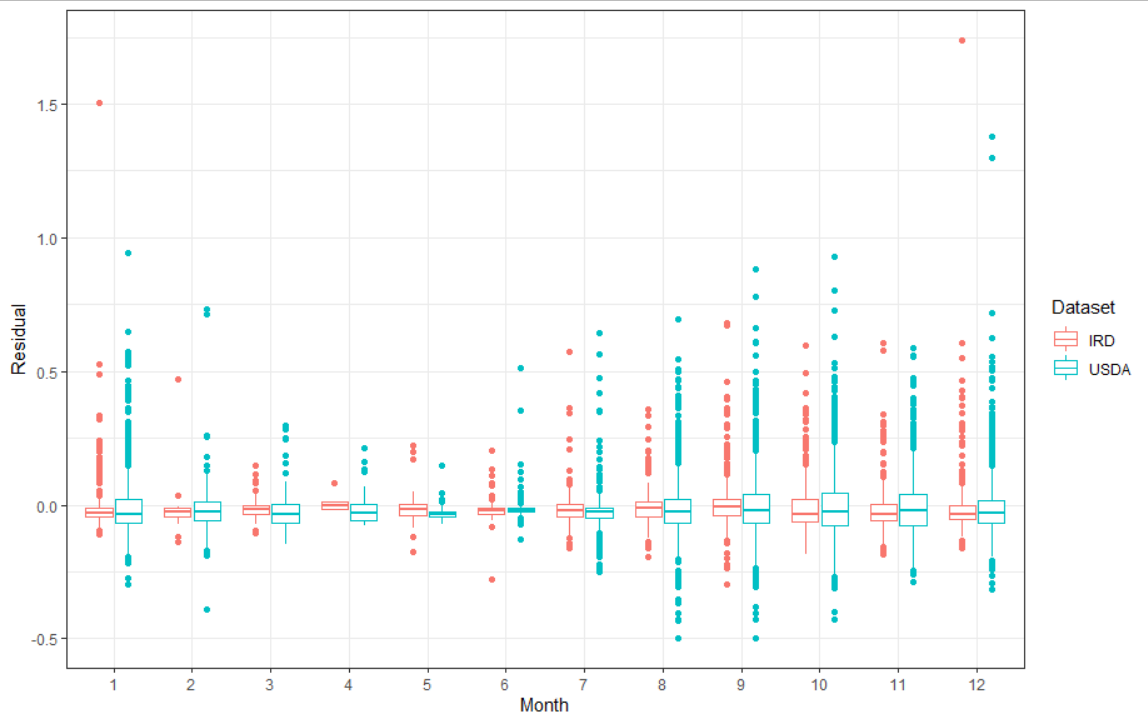


Supplementary Figure S4. Model residuals showing no interaction between dataset and month.


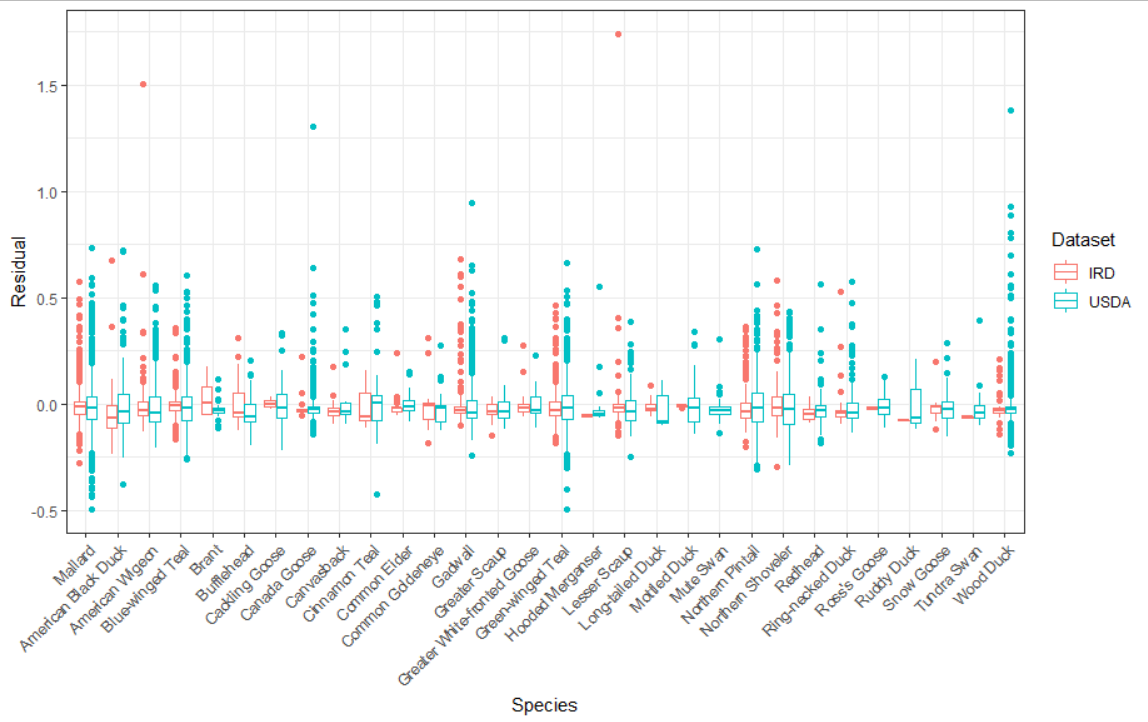


Supplementary Figure S5. Model residuals showing no interaction between dataset and species.


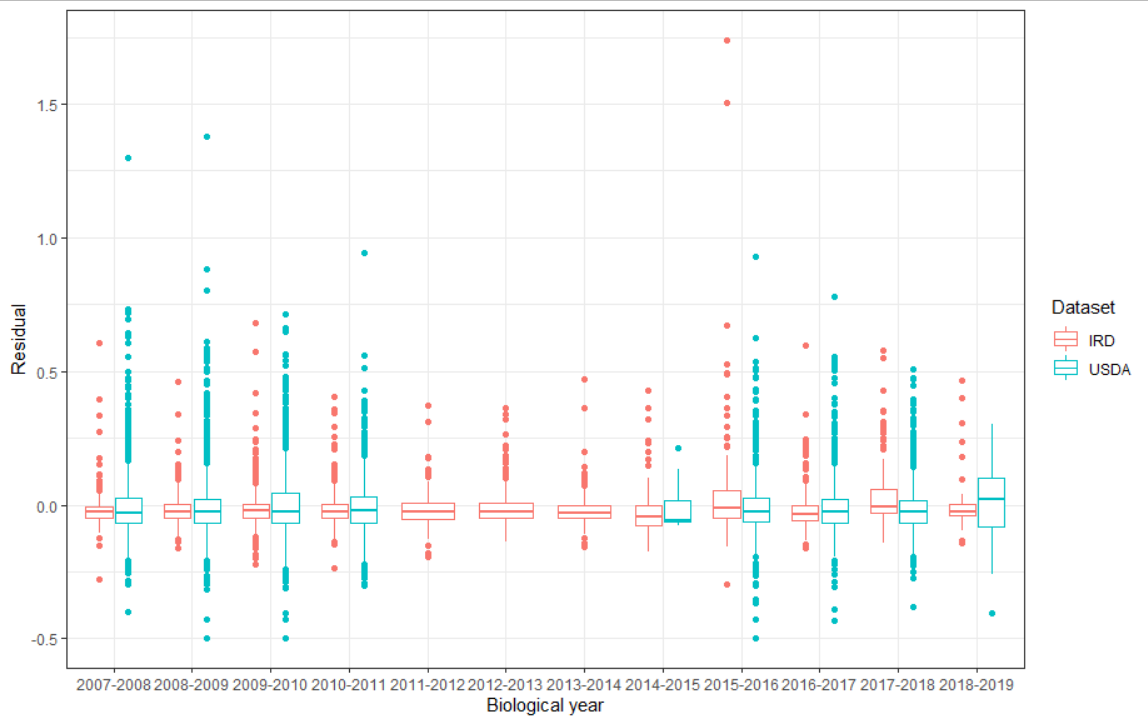


Supplementary Figure S6. Model residuals showing no interaction between dataset and biological year.


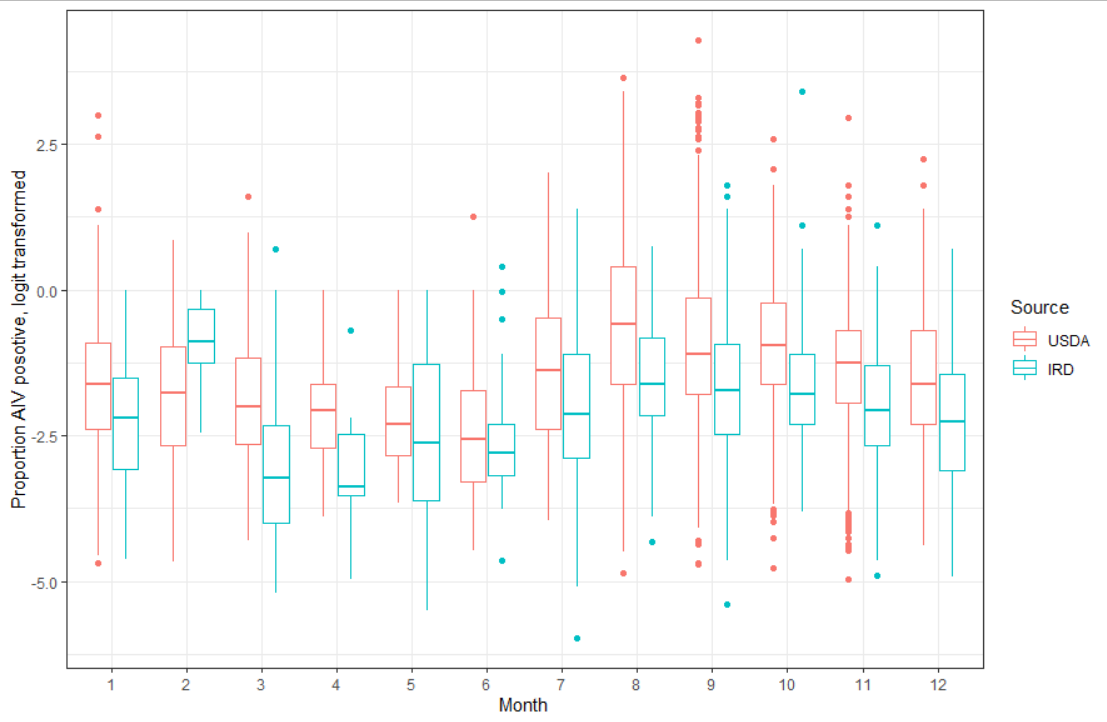
Supplementary Figure S7. Proportion of birds testing positive for IAV in both datasets binned by all prediction variables (species, county, year, and week) by month. Data has been logit transformed for better visualization. USDA consistently has a slightly higher prevalence level than IRD, with the exception of February where there is limited IRD data.


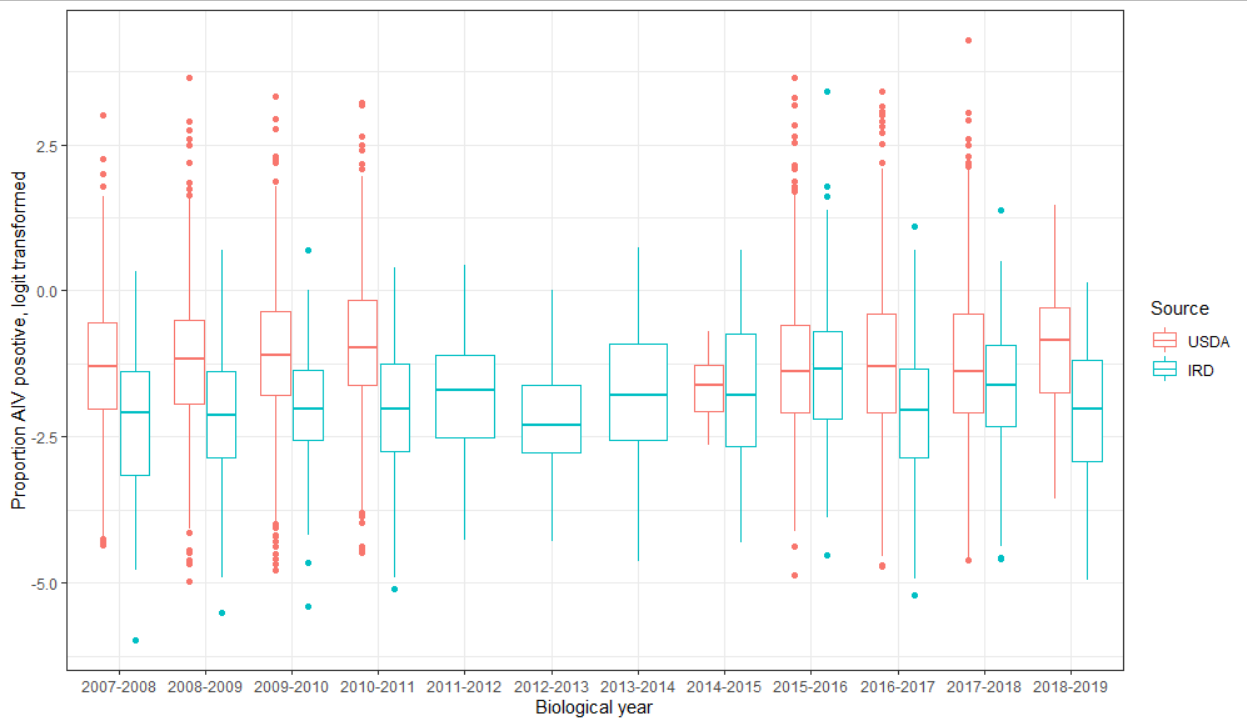


Supplementary Figure S8. Proportion of birds testing positive for IAV in both datasets binned by all prediction variables (species, county, year, and week) by biological year. Data has been logit transformed for better visualization. USDA consistently has a slightly higher prevalence level than IRD.


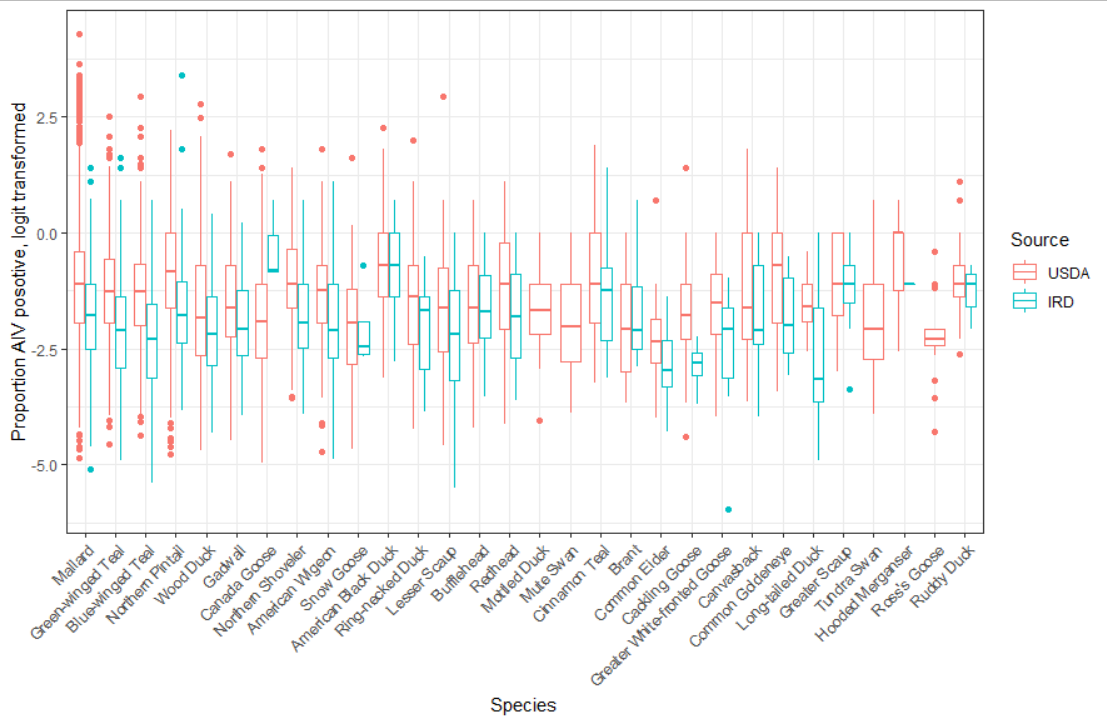
Supplementary Figure S9. Proportion of birds testing positive for IAV in both datasets binned by all prediction variables (species, county, year, and week) by species. Data has been logit transformed for better visualization. Species or sorted in descending order based on the total number of birds sampled. USDA consistently has a slightly higher prevalence level than IRD for well sampled bird species.


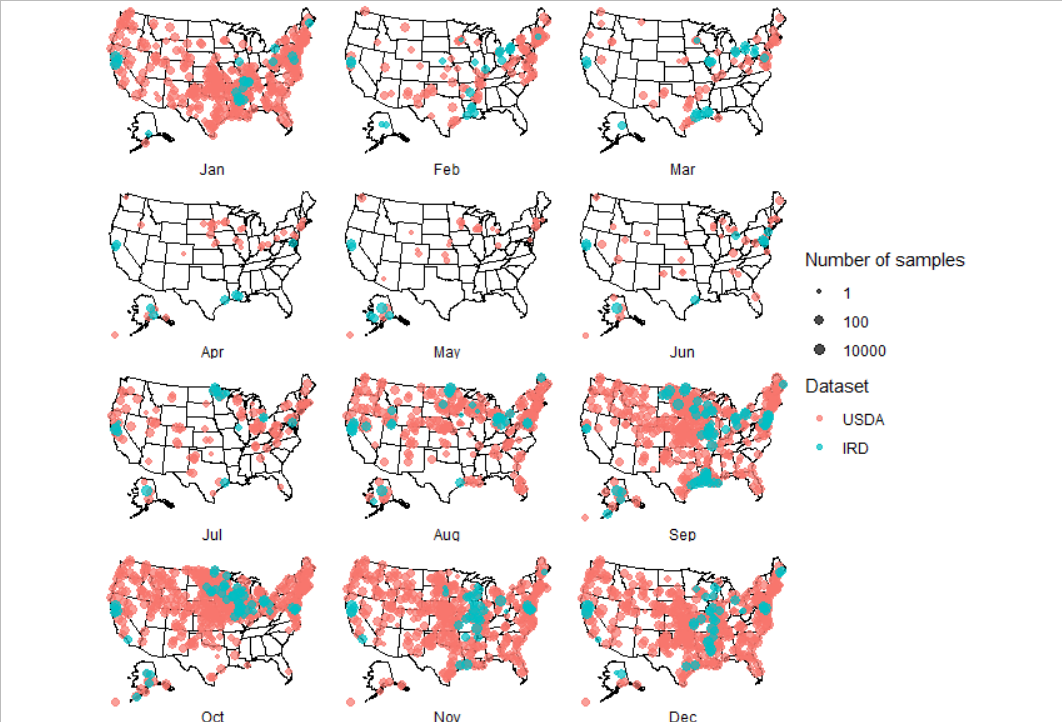


Supplementary Figure S10. Sampling effort of dabbling ducks by month reported by USDA national surveillance program and the NIAID Influenza Research Database datasets.


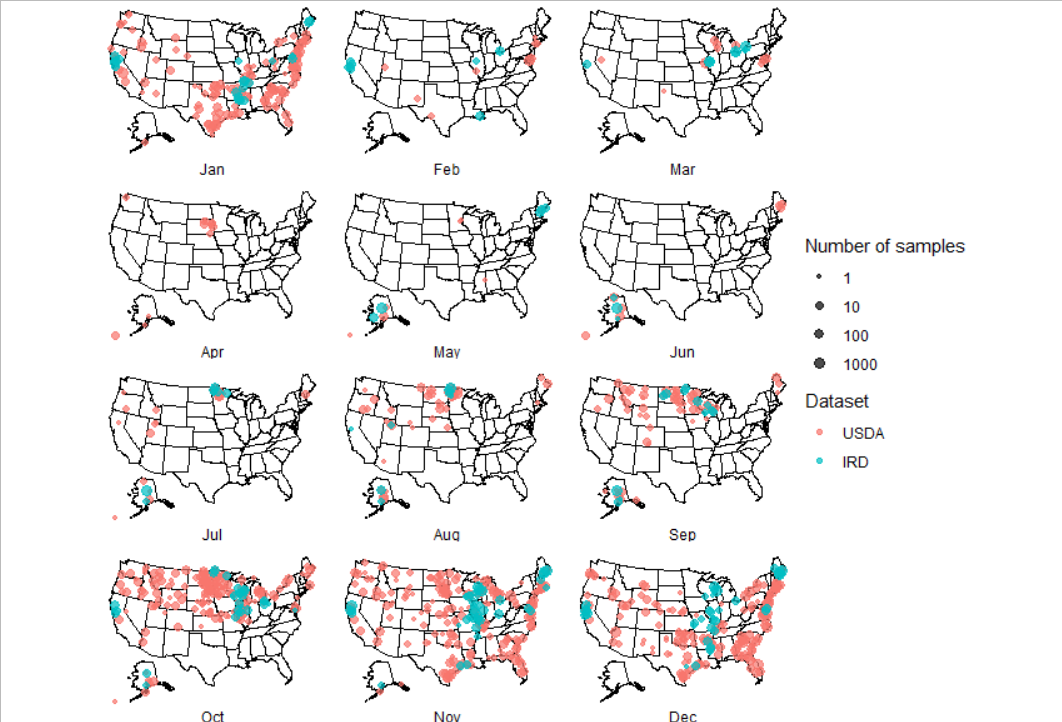


Supplementary Figure S11. Sampling effort of diving and sea ducks by month for both USDA national surveillance program and the NIAID Influenza Research Database datasets.


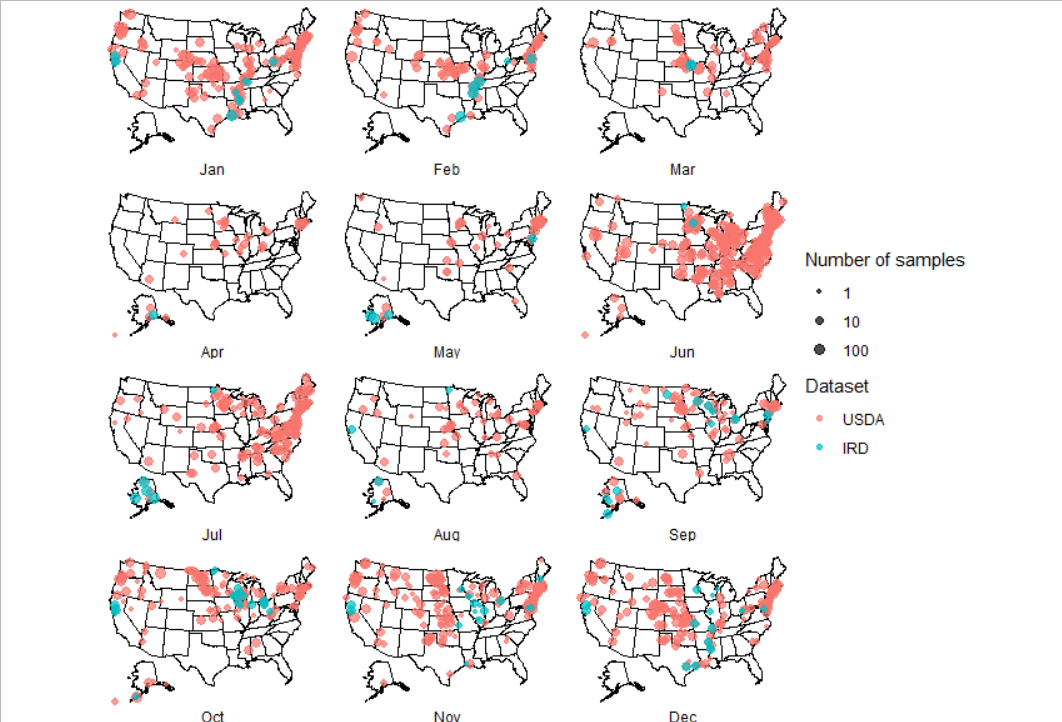


Supplementary Figure S12. Sampling effort of geese by month for both USDA national surveillance program and the NIAID Influenza Research Database datasets.
