# Supplementary figures and images for: Spatiotemporal changes in influenza A virus prevalence among wild waterfowl inhabiting the continental United States throughout the annual cycle
Source: Sci Rep. 2022 Jul 29;12:13083. doi: 10.1038/s41598-022-17396-5 (PMC9338306; doi:10.1038/s41598-022-17396-5)

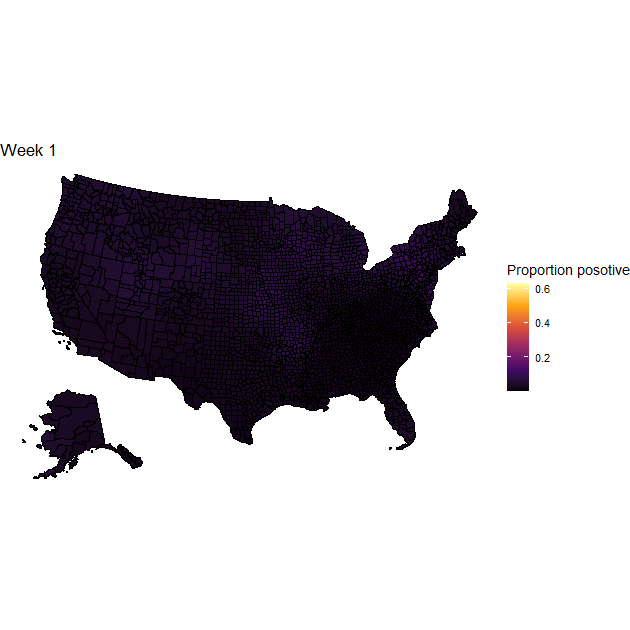

Supplement: Supplementary file 2 — Supplementary Information 2. [file 41598_2022_17396_MOESM2_ESM.zip › GIF/American Black Duck.gif]

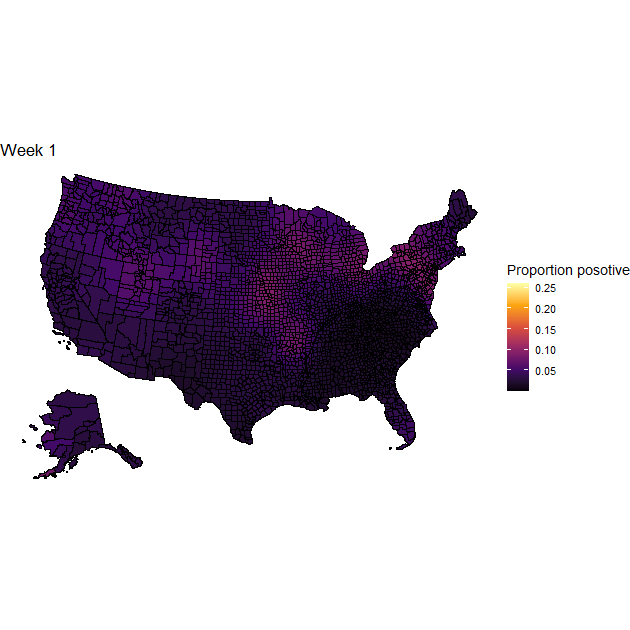

Supplement: Supplementary file 2 — Supplementary Information 2. [file 41598_2022_17396_MOESM2_ESM.zip › GIF/American Wigeon.gif]

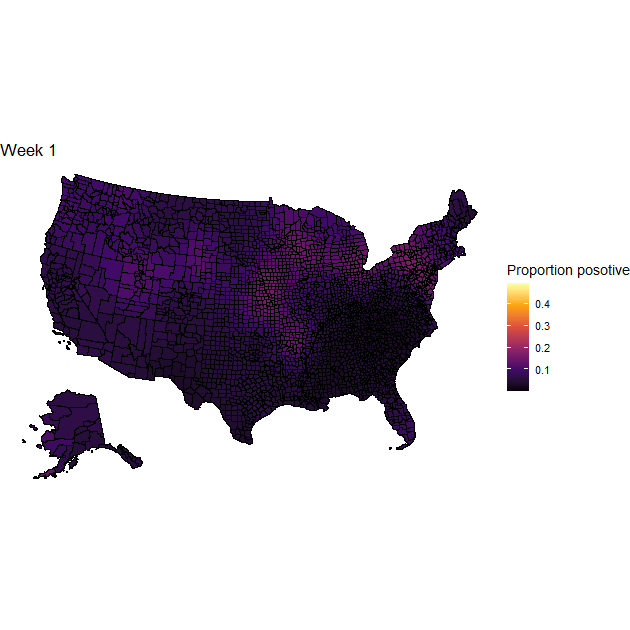

Supplement: Supplementary file 2 — Supplementary Information 2. [file 41598_2022_17396_MOESM2_ESM.zip › GIF/Blue-winged Teal.gif]

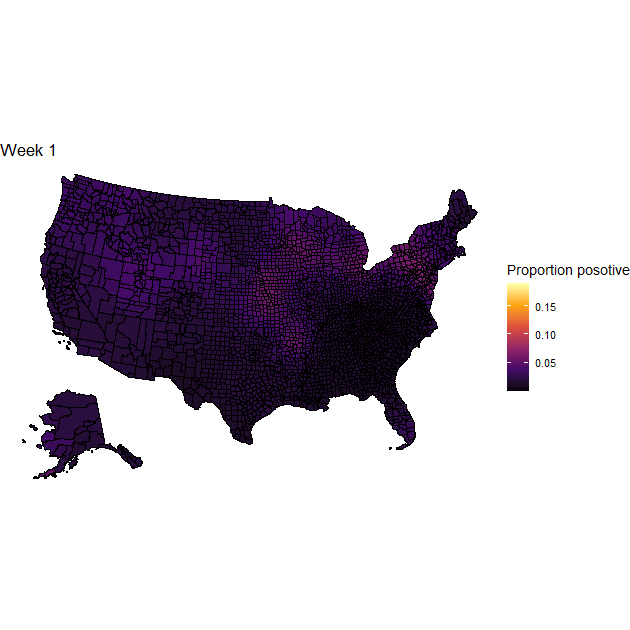

Supplement: Supplementary file 2 — Supplementary Information 2. [file 41598_2022_17396_MOESM2_ESM.zip › GIF/Brant.gif]

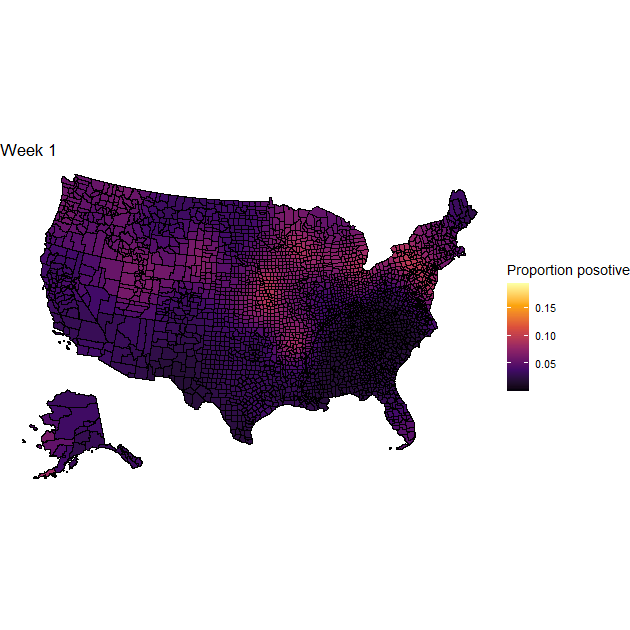

Supplement: Supplementary file 2 — Supplementary Information 2. [file 41598_2022_17396_MOESM2_ESM.zip › GIF/Bufflehead.gif]

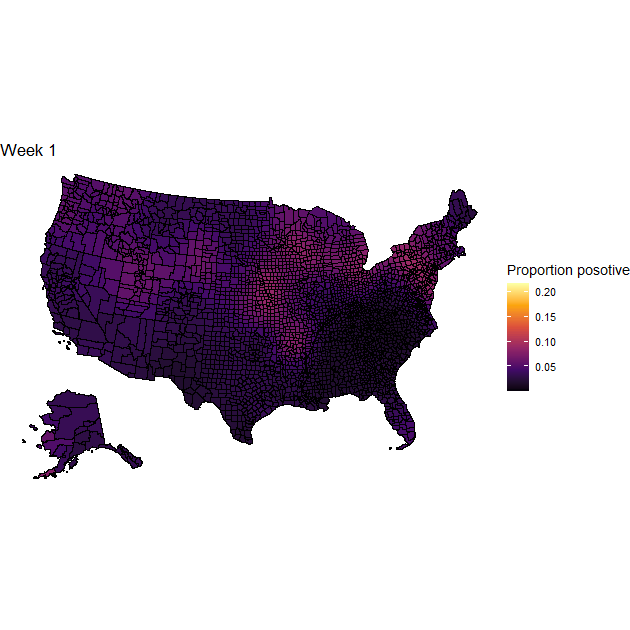

Supplement: Supplementary file 2 — Supplementary Information 2. [file 41598_2022_17396_MOESM2_ESM.zip › GIF/Cackling Goose.gif]

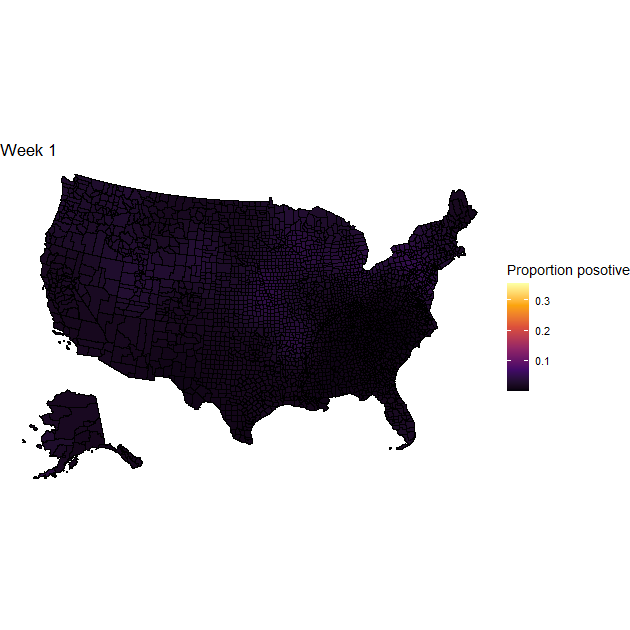

Supplement: Supplementary file 2 — Supplementary Information 2. [file 41598_2022_17396_MOESM2_ESM.zip › GIF/Canada Goose.gif]

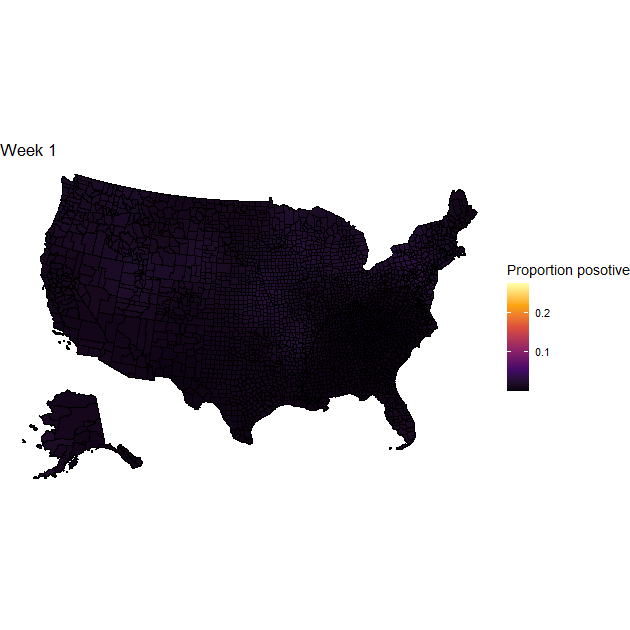

Supplement: Supplementary file 2 — Supplementary Information 2. [file 41598_2022_17396_MOESM2_ESM.zip › GIF/Canvasback.gif]

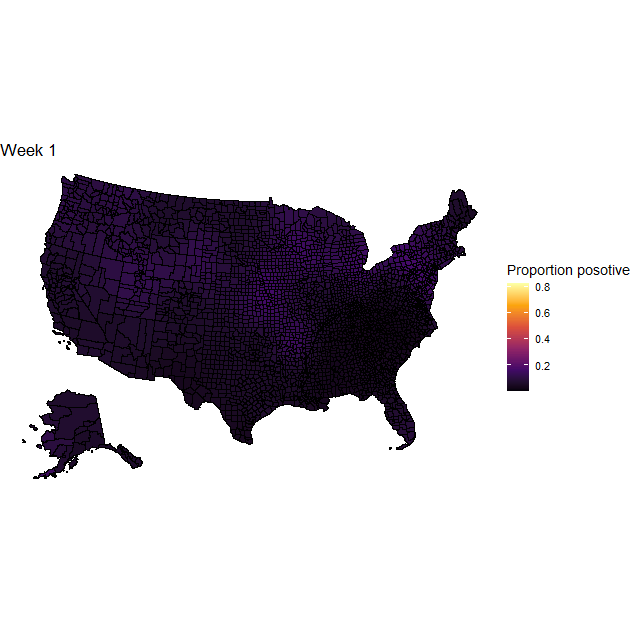

Supplement: Supplementary file 2 — Supplementary Information 2. [file 41598_2022_17396_MOESM2_ESM.zip › GIF/Cinnamon Teal.gif]

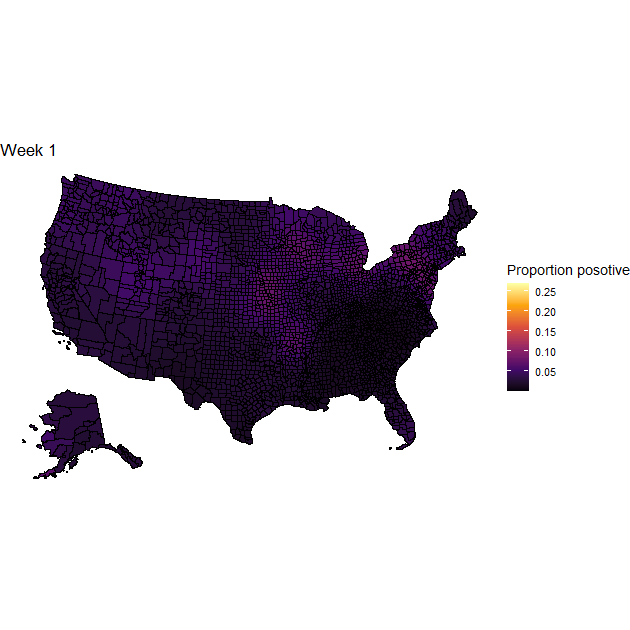

Supplement: Supplementary file 2 — Supplementary Information 2. [file 41598_2022_17396_MOESM2_ESM.zip › GIF/Common Eider.gif]

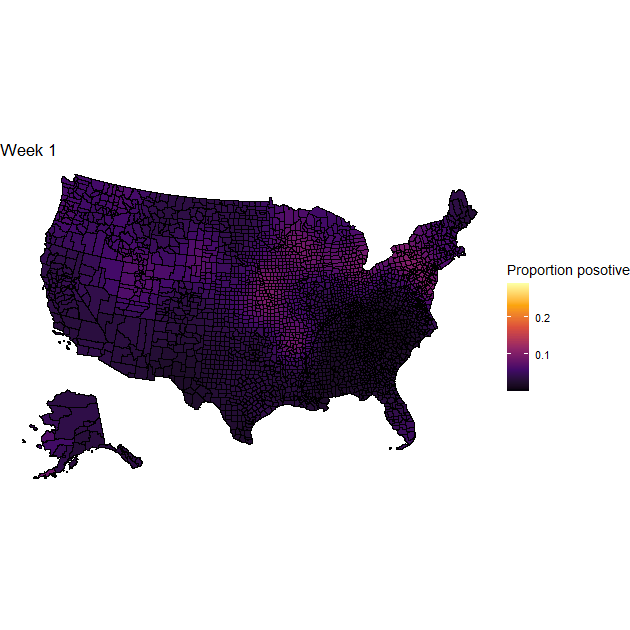

Supplement: Supplementary file 2 — Supplementary Information 2. [file 41598_2022_17396_MOESM2_ESM.zip › GIF/Common Goldeneye.gif]

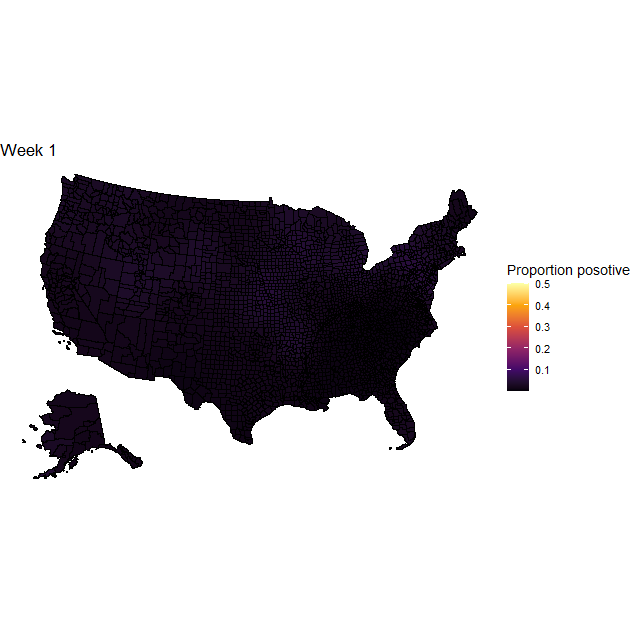

Supplement: Supplementary file 2 — Supplementary Information 2. [file 41598_2022_17396_MOESM2_ESM.zip › GIF/Gadwall.gif]

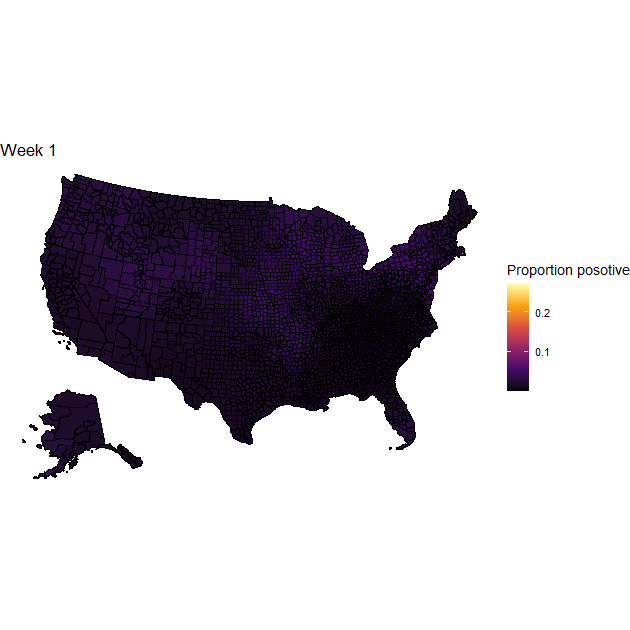

Supplement: Supplementary file 2 — Supplementary Information 2. [file 41598_2022_17396_MOESM2_ESM.zip › GIF/Greater Scaup.gif]

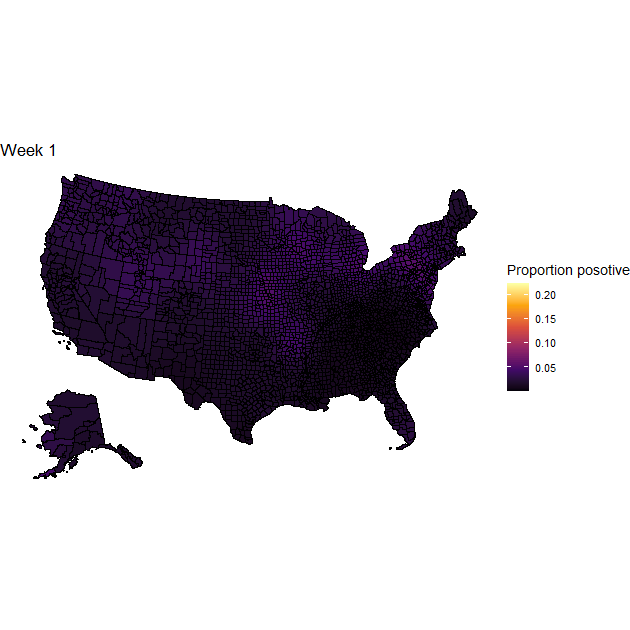

Supplement: Supplementary file 2 — Supplementary Information 2. [file 41598_2022_17396_MOESM2_ESM.zip › GIF/Greater White-fronted Goose.gif]

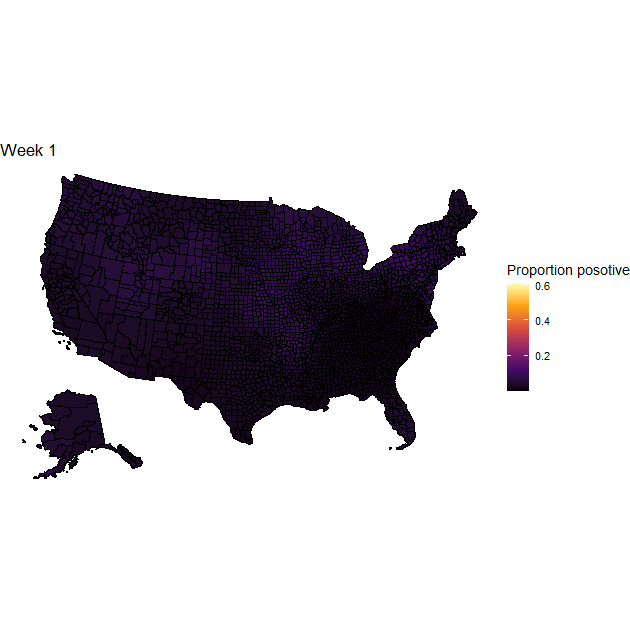

Supplement: Supplementary file 2 — Supplementary Information 2. [file 41598_2022_17396_MOESM2_ESM.zip › GIF/Green-winged Teal.gif]

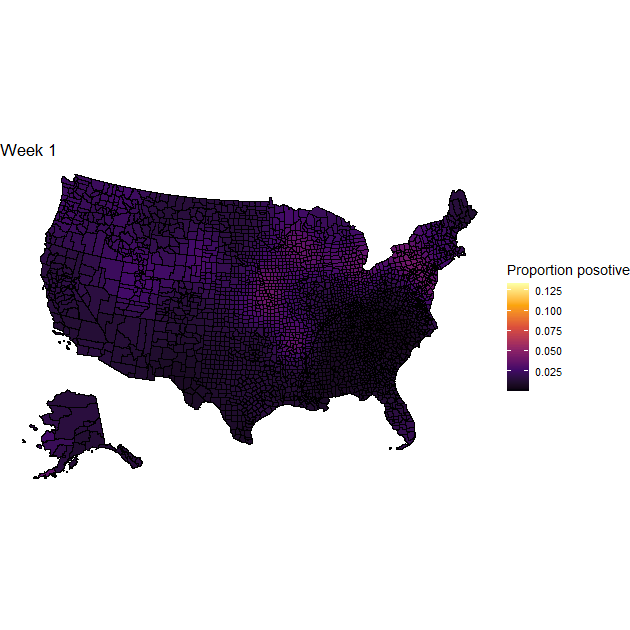

Supplement: Supplementary file 2 — Supplementary Information 2. [file 41598_2022_17396_MOESM2_ESM.zip › GIF/Hooded Merganser.gif]

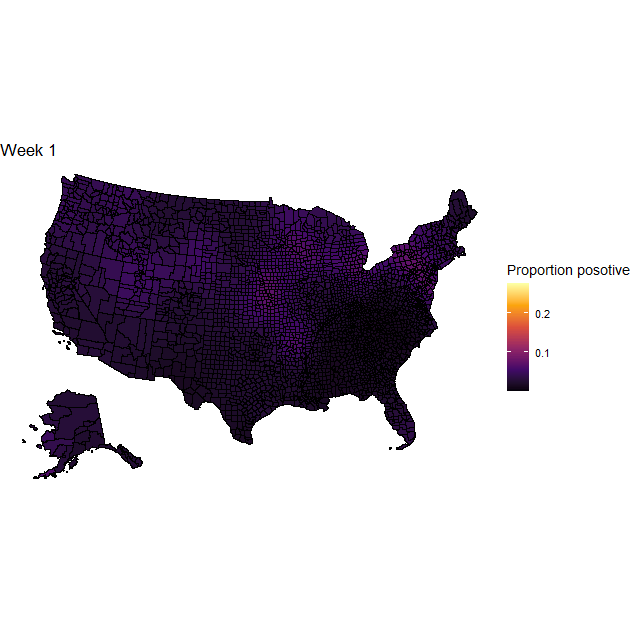

Supplement: Supplementary file 2 — Supplementary Information 2. [file 41598_2022_17396_MOESM2_ESM.zip › GIF/Lesser Scaup.gif]

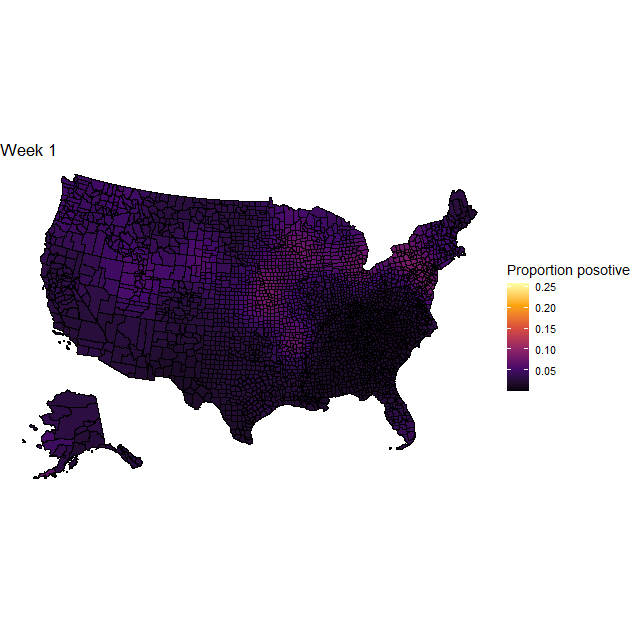

Supplement: Supplementary file 2 — Supplementary Information 2. [file 41598_2022_17396_MOESM2_ESM.zip › GIF/Long-tailed Duck.gif]

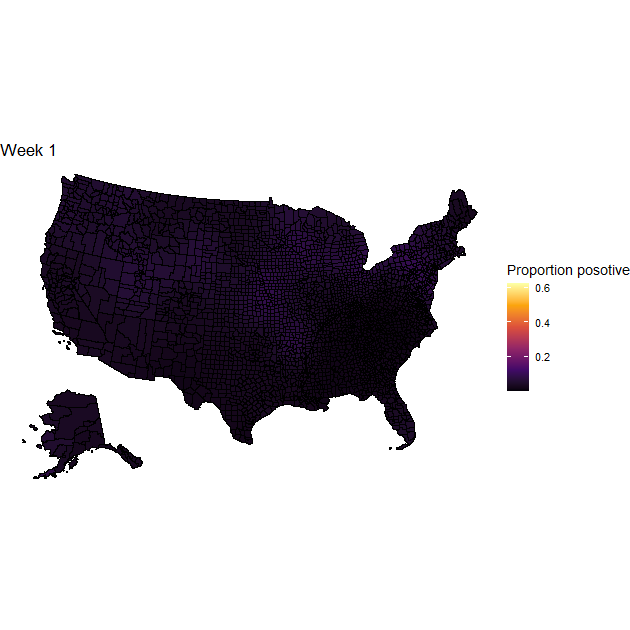

Supplement: Supplementary file 2 — Supplementary Information 2. [file 41598_2022_17396_MOESM2_ESM.zip › GIF/Mallard.gif]

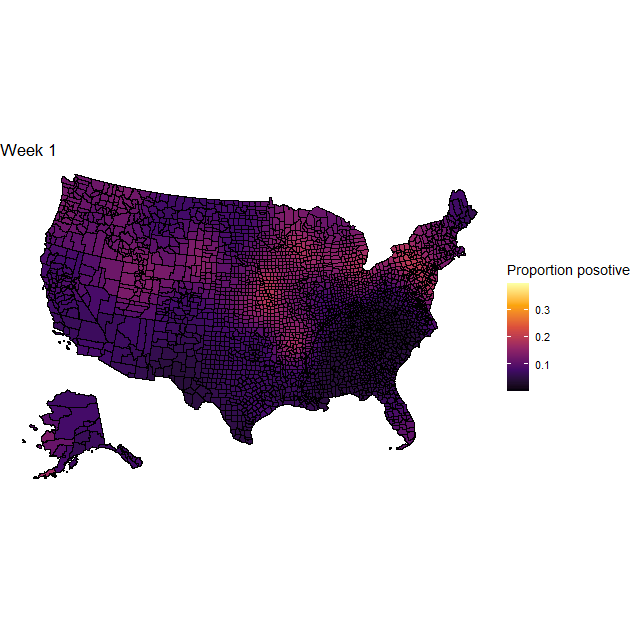

Supplement: Supplementary file 2 — Supplementary Information 2. [file 41598_2022_17396_MOESM2_ESM.zip › GIF/Mottled Duck.gif]

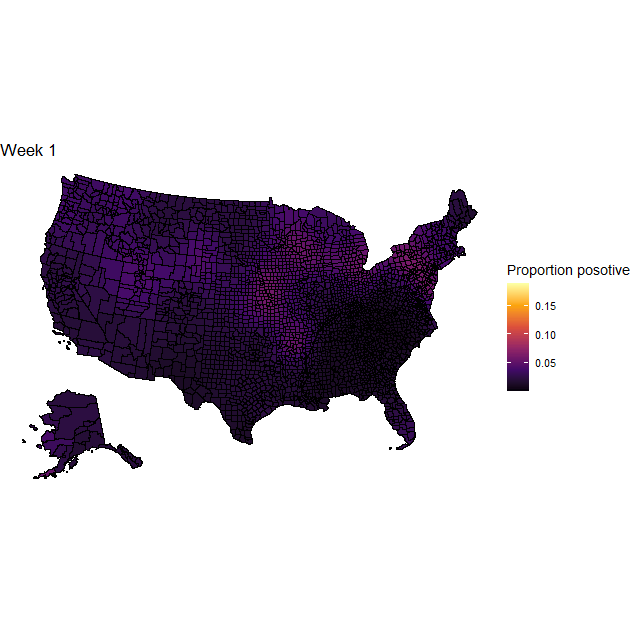

Supplement: Supplementary file 2 — Supplementary Information 2. [file 41598_2022_17396_MOESM2_ESM.zip › GIF/Mute Swan.gif]

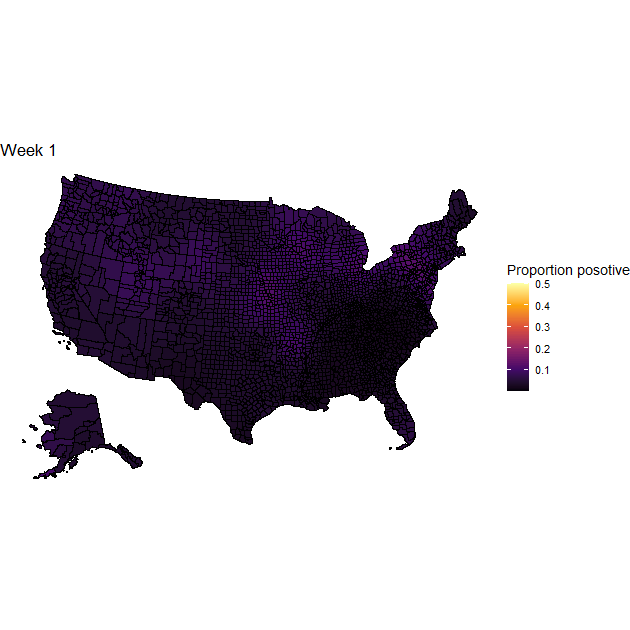

Supplement: Supplementary file 2 — Supplementary Information 2. [file 41598_2022_17396_MOESM2_ESM.zip › GIF/Northern Pintail.gif]

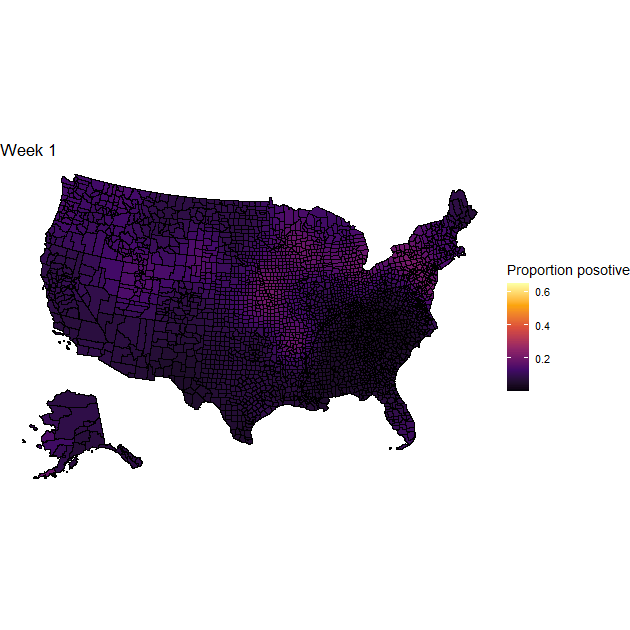

Supplement: Supplementary file 2 — Supplementary Information 2. [file 41598_2022_17396_MOESM2_ESM.zip › GIF/Northern Shoveler.gif]

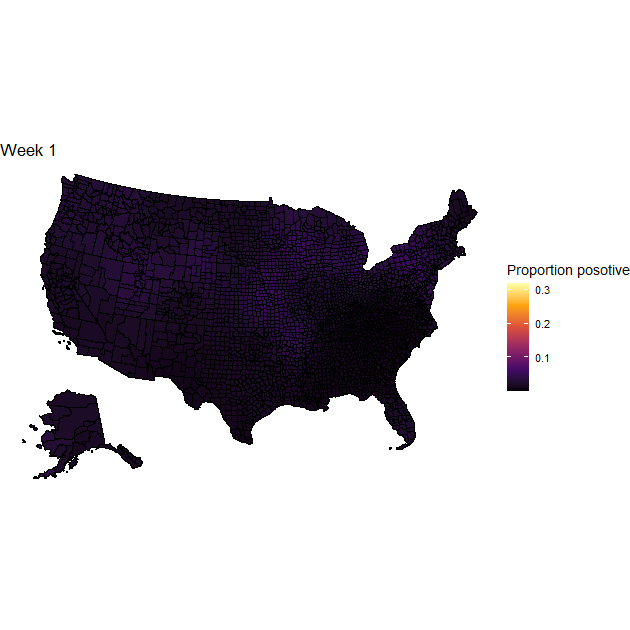

Supplement: Supplementary file 2 — Supplementary Information 2. [file 41598_2022_17396_MOESM2_ESM.zip › GIF/Redhead.gif]

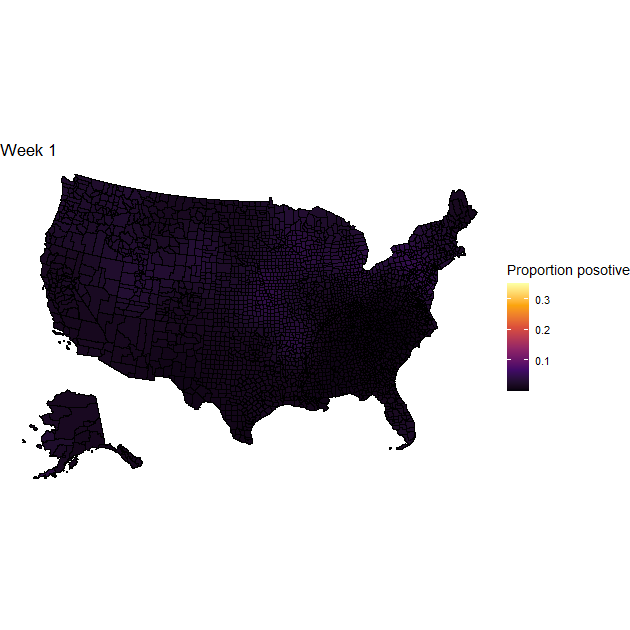

Supplement: Supplementary file 2 — Supplementary Information 2. [file 41598_2022_17396_MOESM2_ESM.zip › GIF/Ring-necked Duck.gif]

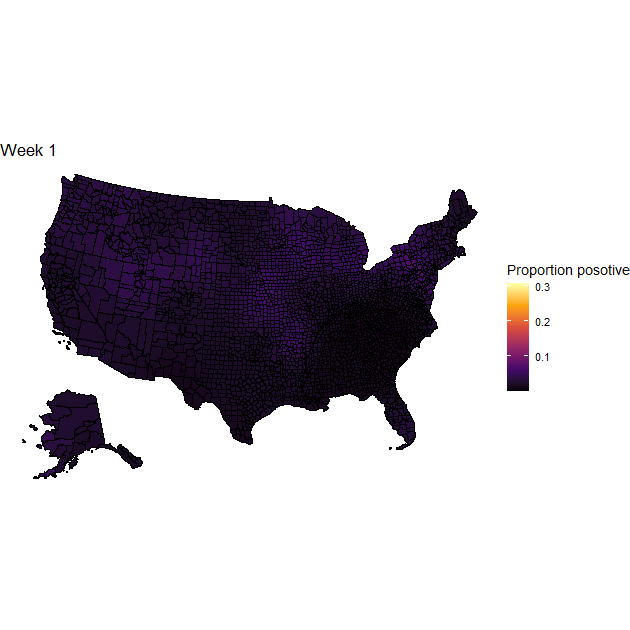

Supplement: Supplementary file 2 — Supplementary Information 2. [file 41598_2022_17396_MOESM2_ESM.zip › GIF/Ross's Goose.gif]

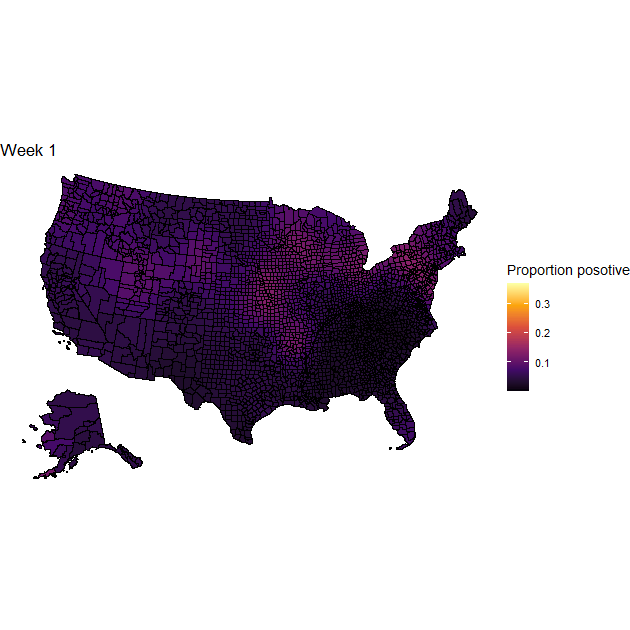

Supplement: Supplementary file 2 — Supplementary Information 2. [file 41598_2022_17396_MOESM2_ESM.zip › GIF/Ruddy Duck.gif]

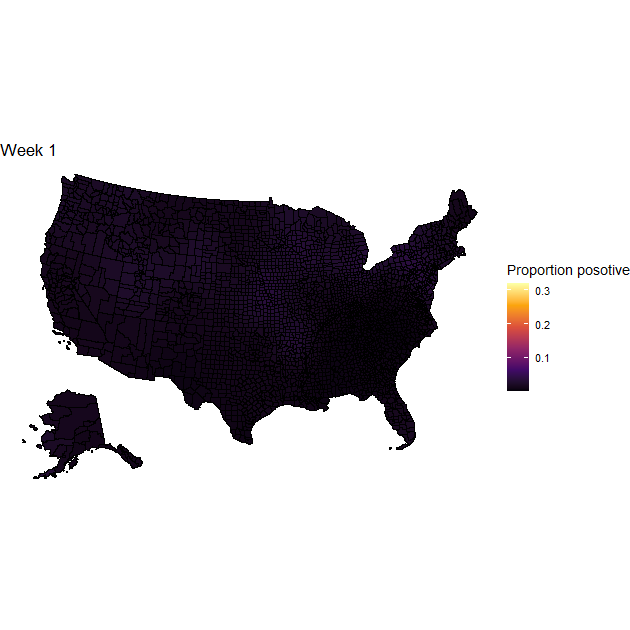

Supplement: Supplementary file 2 — Supplementary Information 2. [file 41598_2022_17396_MOESM2_ESM.zip › GIF/Snow Goose.gif]

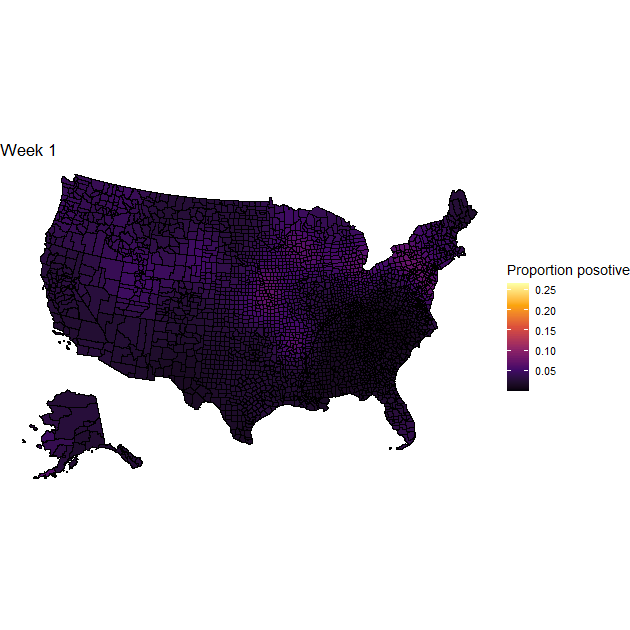

Supplement: Supplementary file 2 — Supplementary Information 2. [file 41598_2022_17396_MOESM2_ESM.zip › GIF/Tundra Swan.gif]

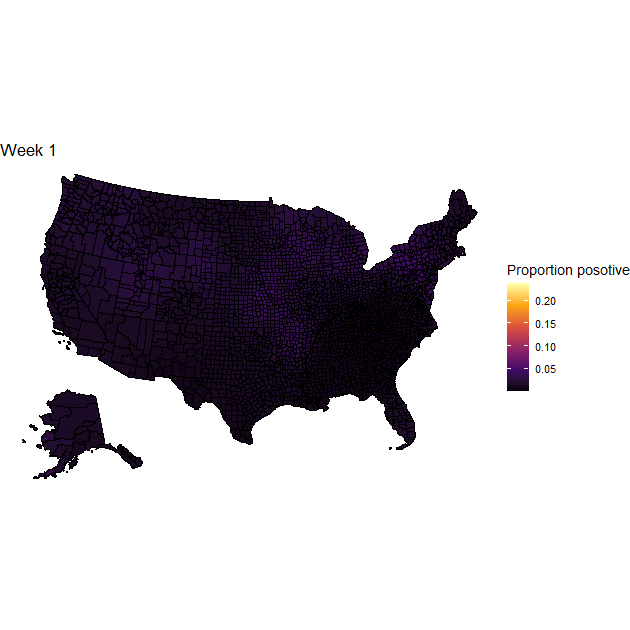

Supplement: Supplementary file 2 — Supplementary Information 2. [file 41598_2022_17396_MOESM2_ESM.zip › GIF/Wood Duck.gif]

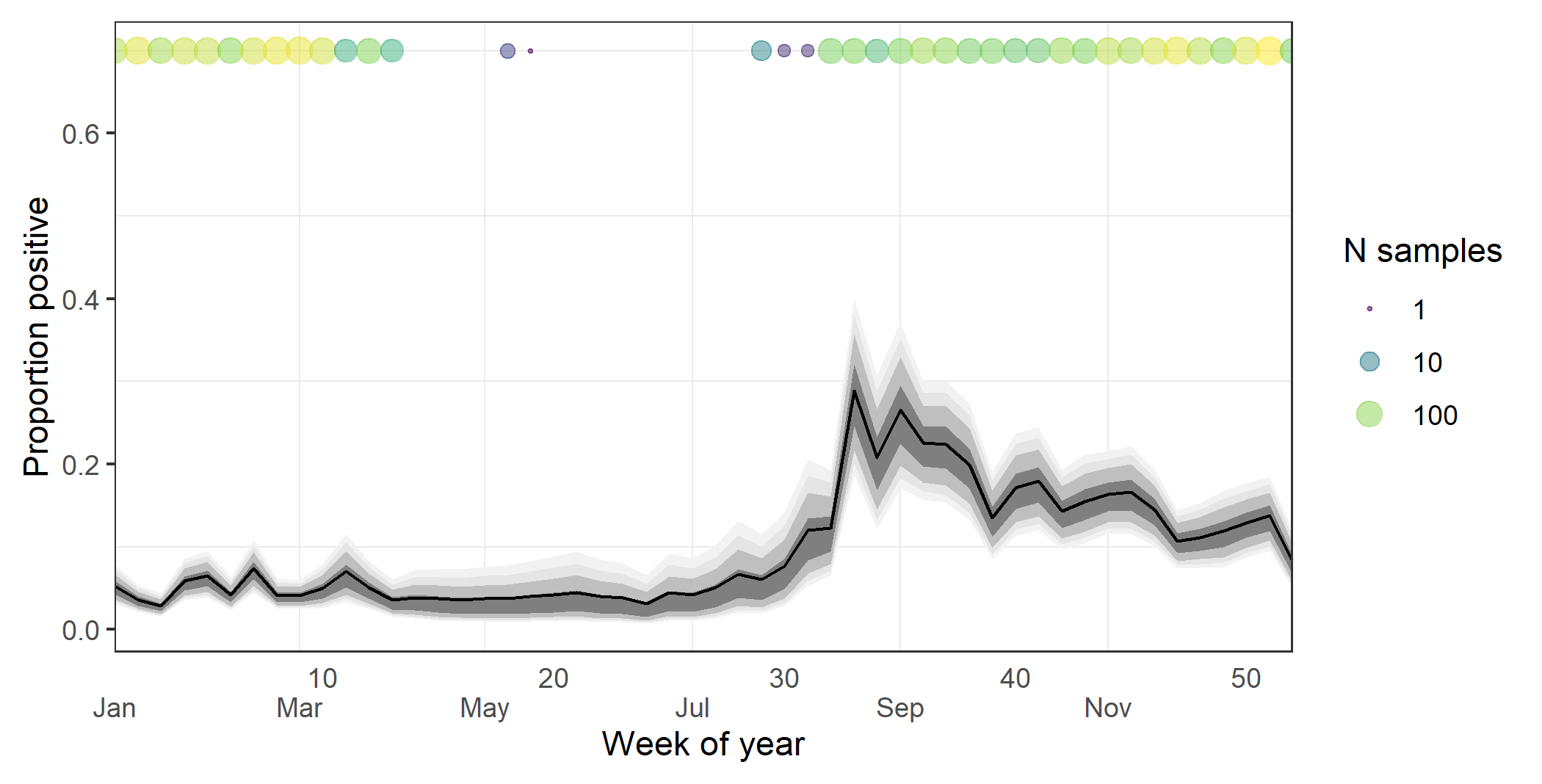

Supplement: Supplementary file 3 — Supplementary Information 3. [file 41598_2022_17396_MOESM3_ESM.zip › SupplementaryMaterials3/American Black Duck.png]

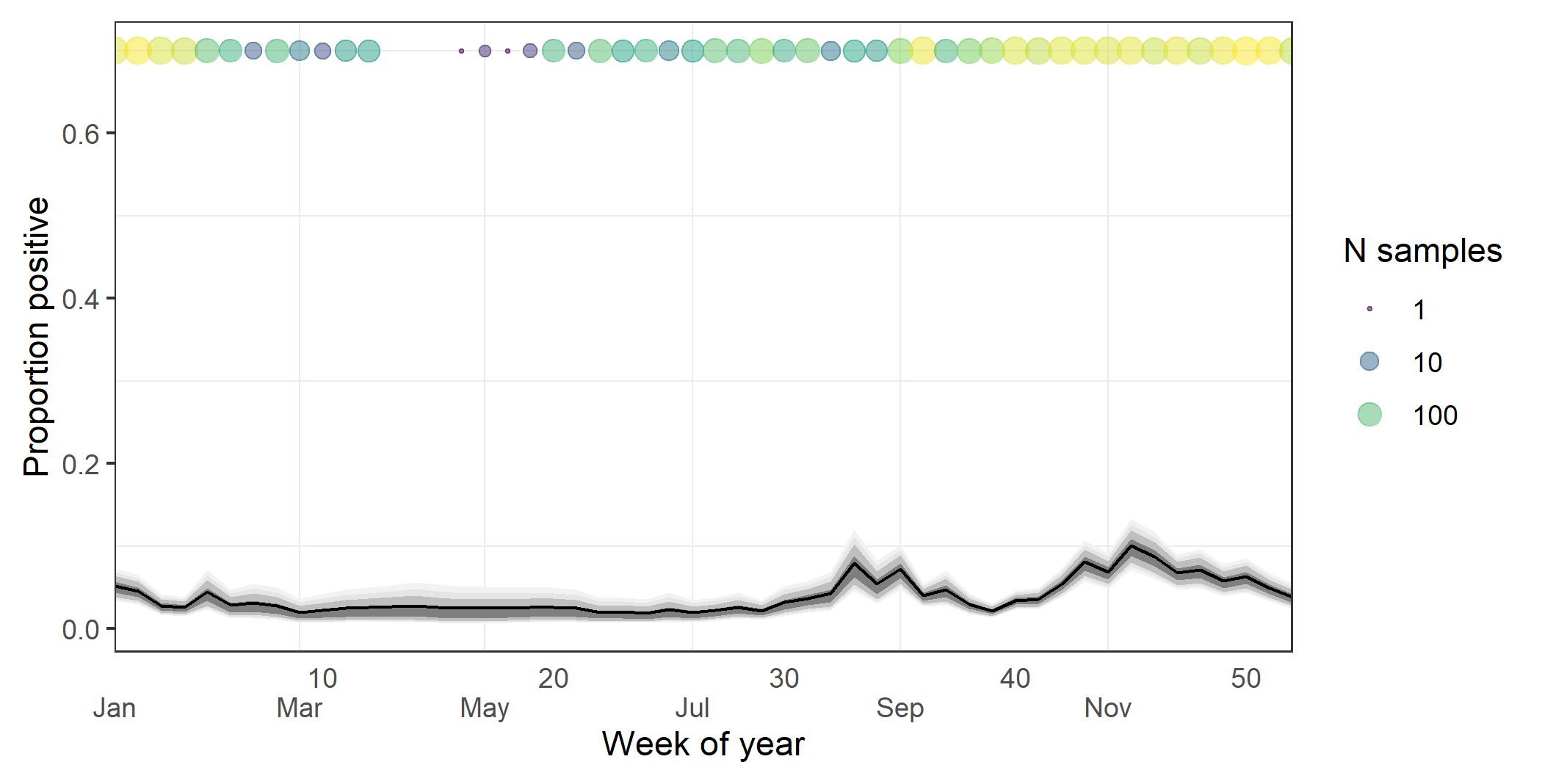

Supplement: Supplementary file 3 — Supplementary Information 3. [file 41598_2022_17396_MOESM3_ESM.zip › SupplementaryMaterials3/American Wigeon.png]

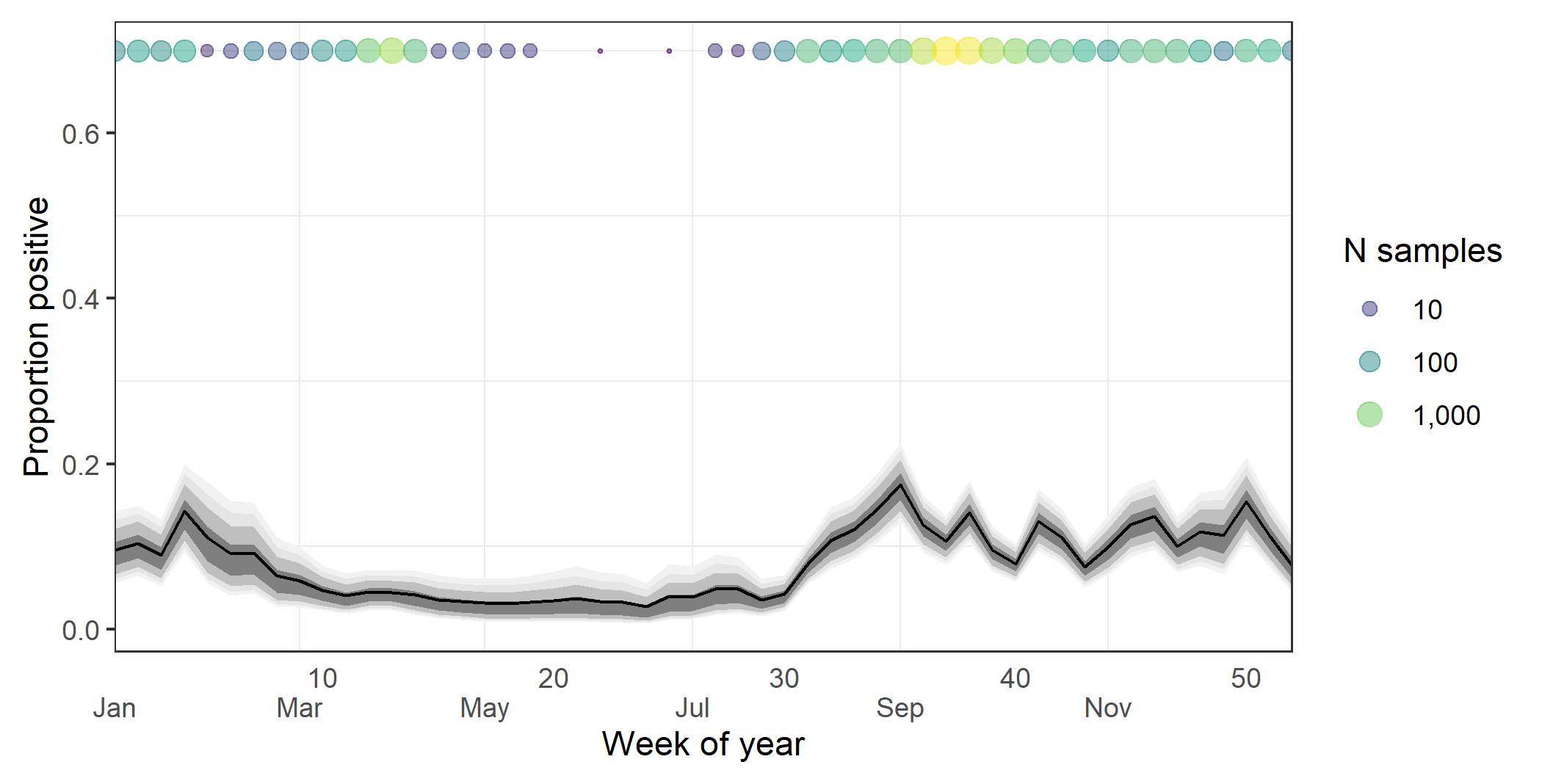

Supplement: Supplementary file 3 — Supplementary Information 3. [file 41598_2022_17396_MOESM3_ESM.zip › SupplementaryMaterials3/Blue-winged Teal.png]

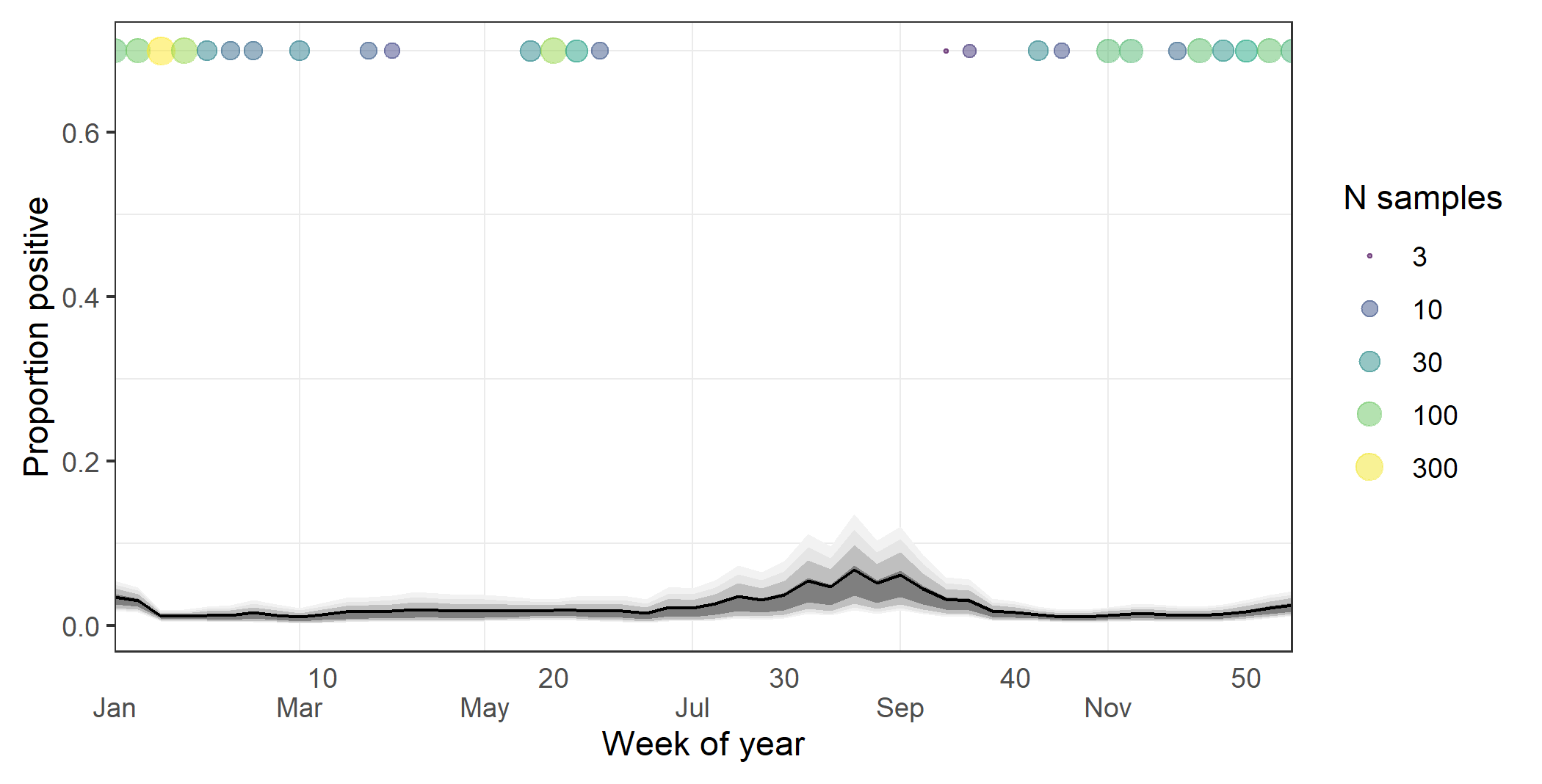

Supplement: Supplementary file 3 — Supplementary Information 3. [file 41598_2022_17396_MOESM3_ESM.zip › SupplementaryMaterials3/Brant.png]

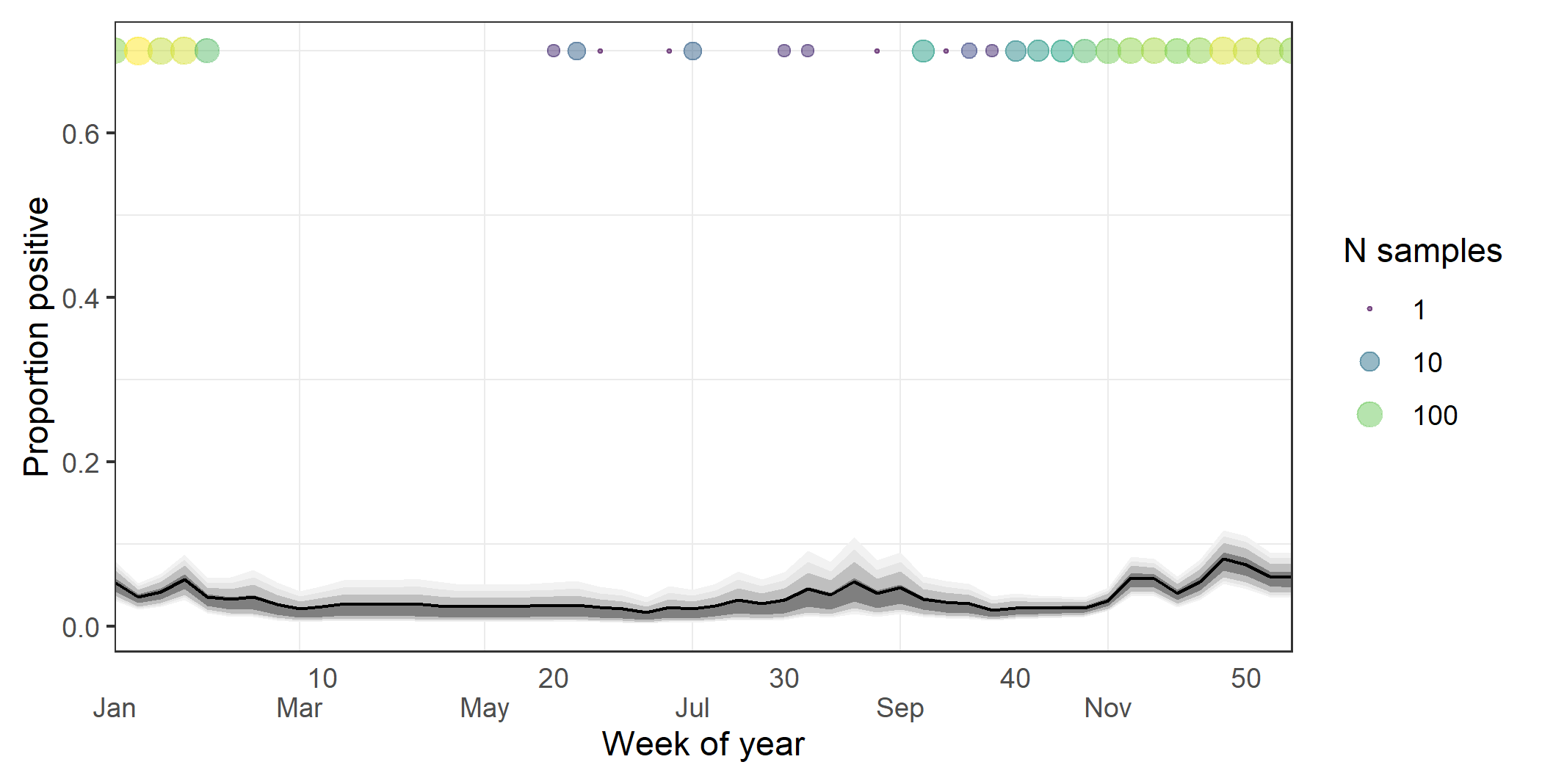

Supplement: Supplementary file 3 — Supplementary Information 3. [file 41598_2022_17396_MOESM3_ESM.zip › SupplementaryMaterials3/Bufflehead.png]

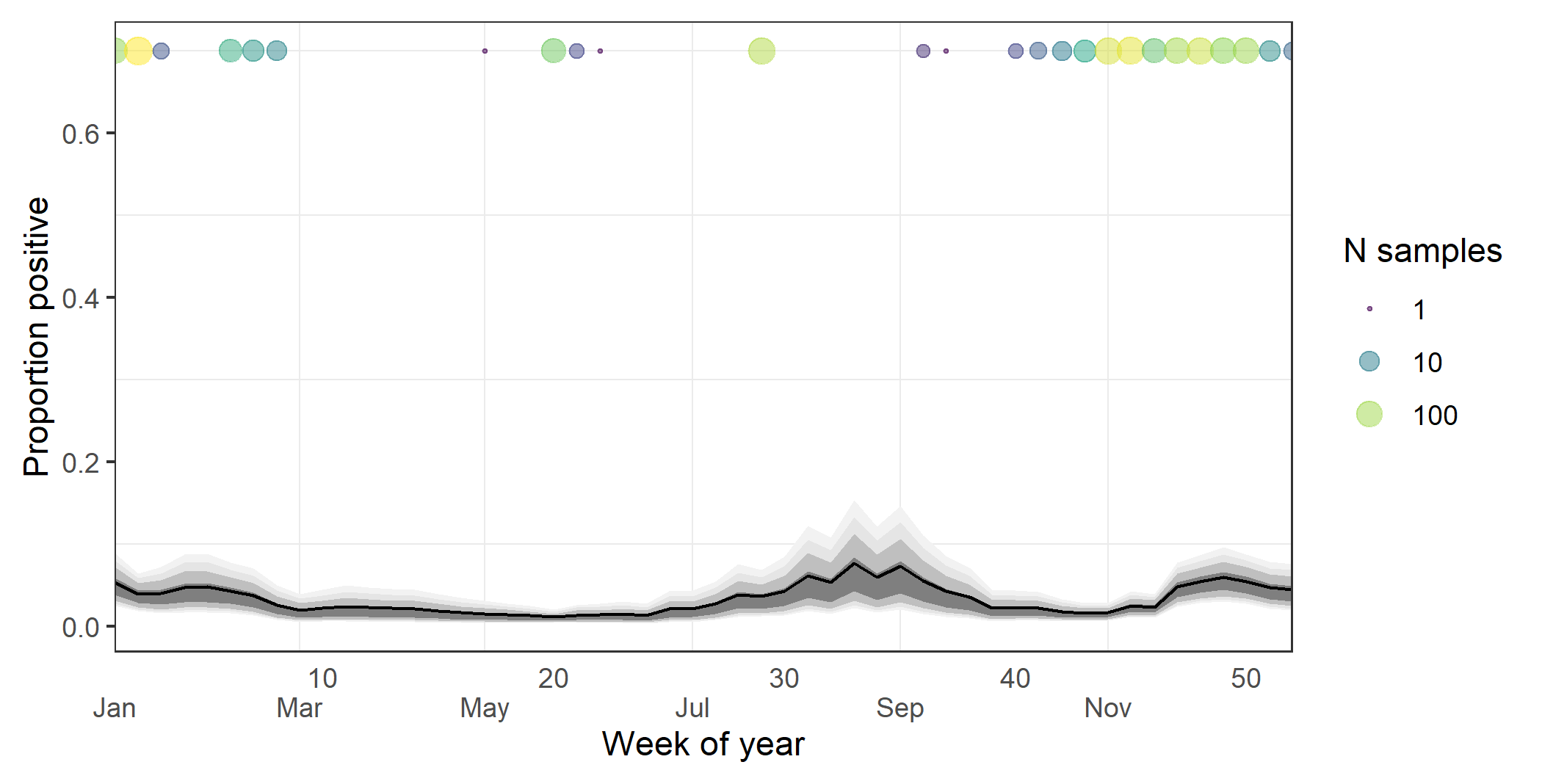

Supplement: Supplementary file 3 — Supplementary Information 3. [file 41598_2022_17396_MOESM3_ESM.zip › SupplementaryMaterials3/Cackling Goose.png]

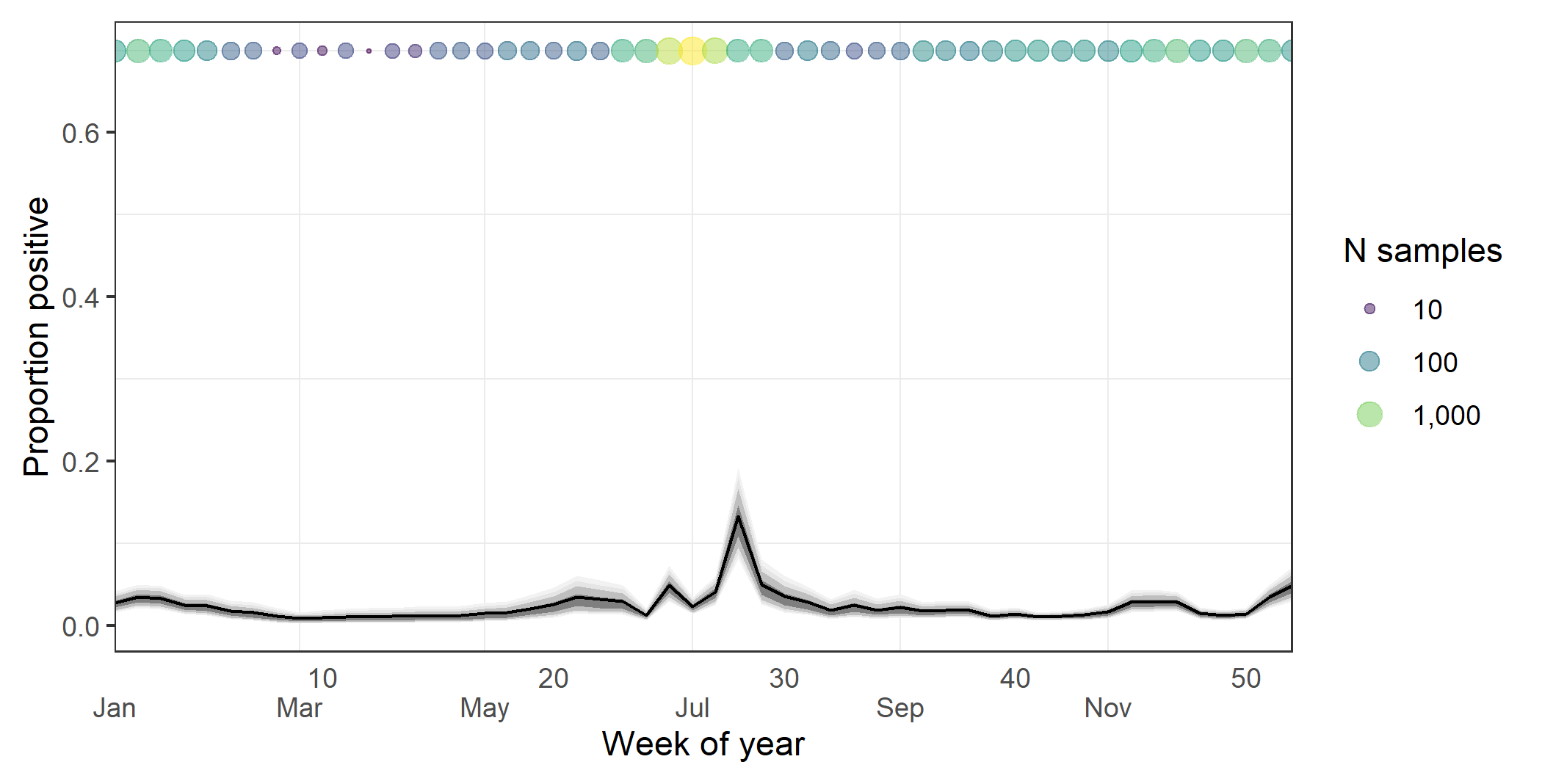

Supplement: Supplementary file 3 — Supplementary Information 3. [file 41598_2022_17396_MOESM3_ESM.zip › SupplementaryMaterials3/Canada Goose.png]

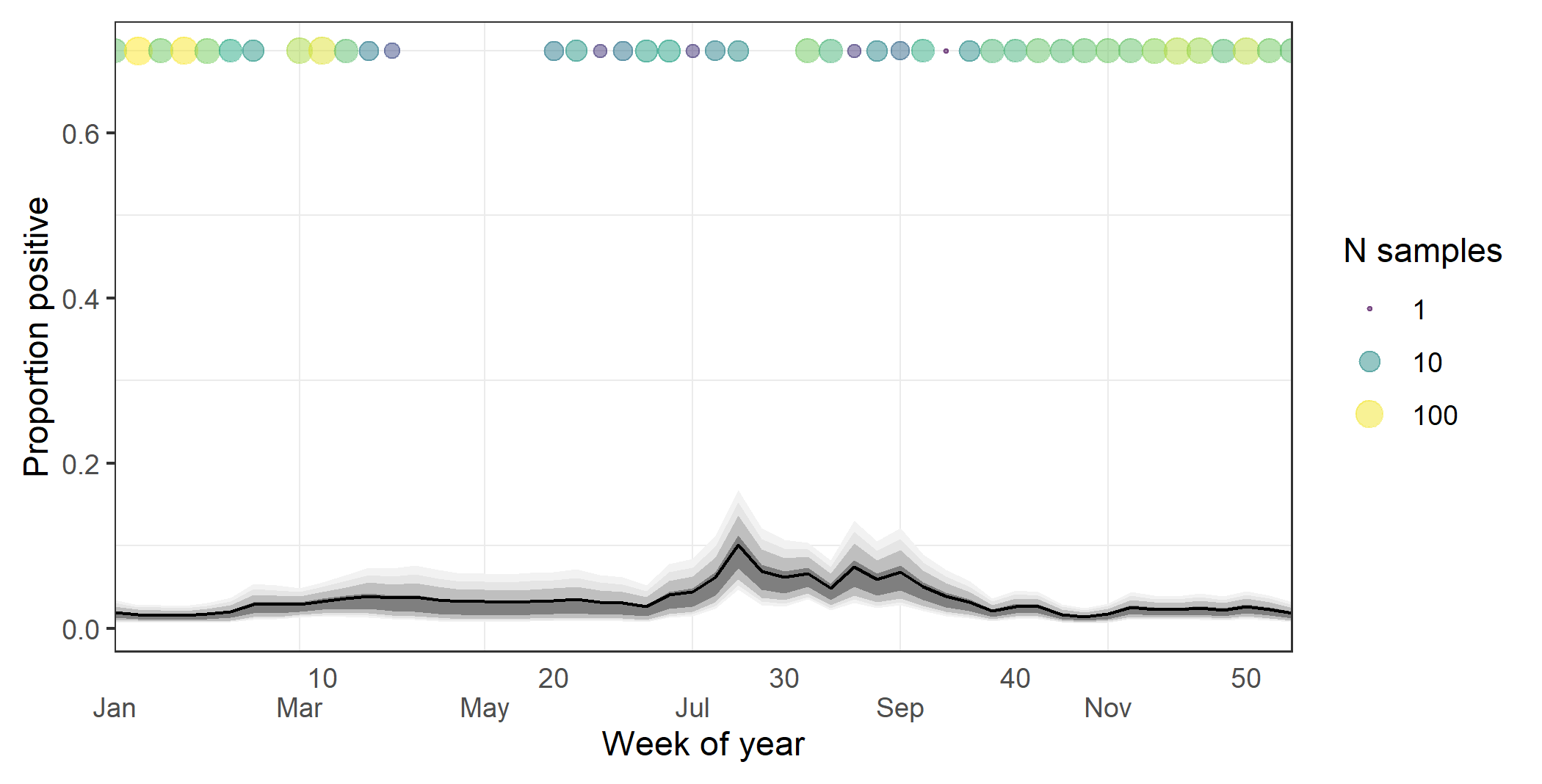

Supplement: Supplementary file 3 — Supplementary Information 3. [file 41598_2022_17396_MOESM3_ESM.zip › SupplementaryMaterials3/Canvasback.png]

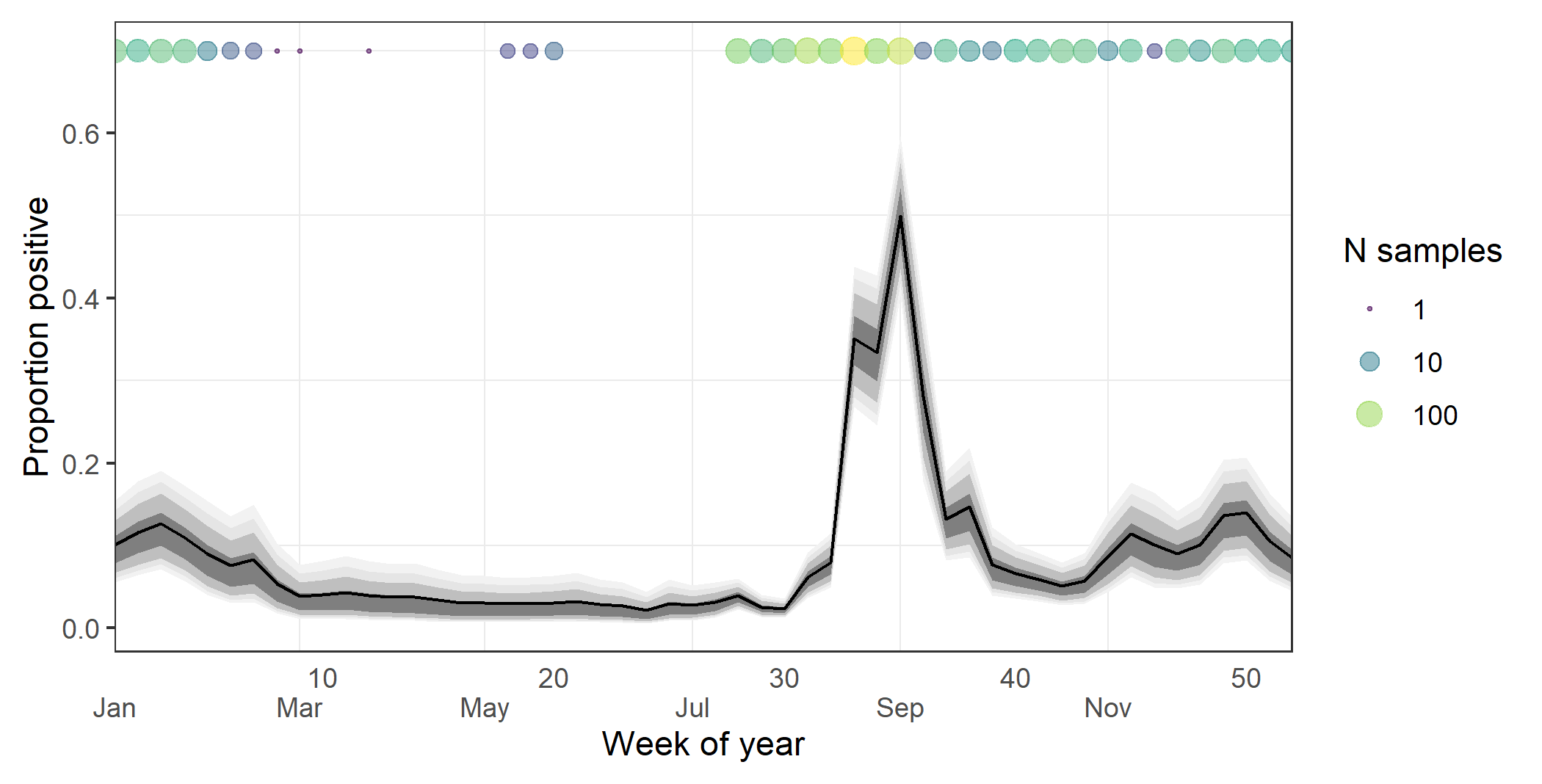

Supplement: Supplementary file 3 — Supplementary Information 3. [file 41598_2022_17396_MOESM3_ESM.zip › SupplementaryMaterials3/Cinnamon Teal.png]

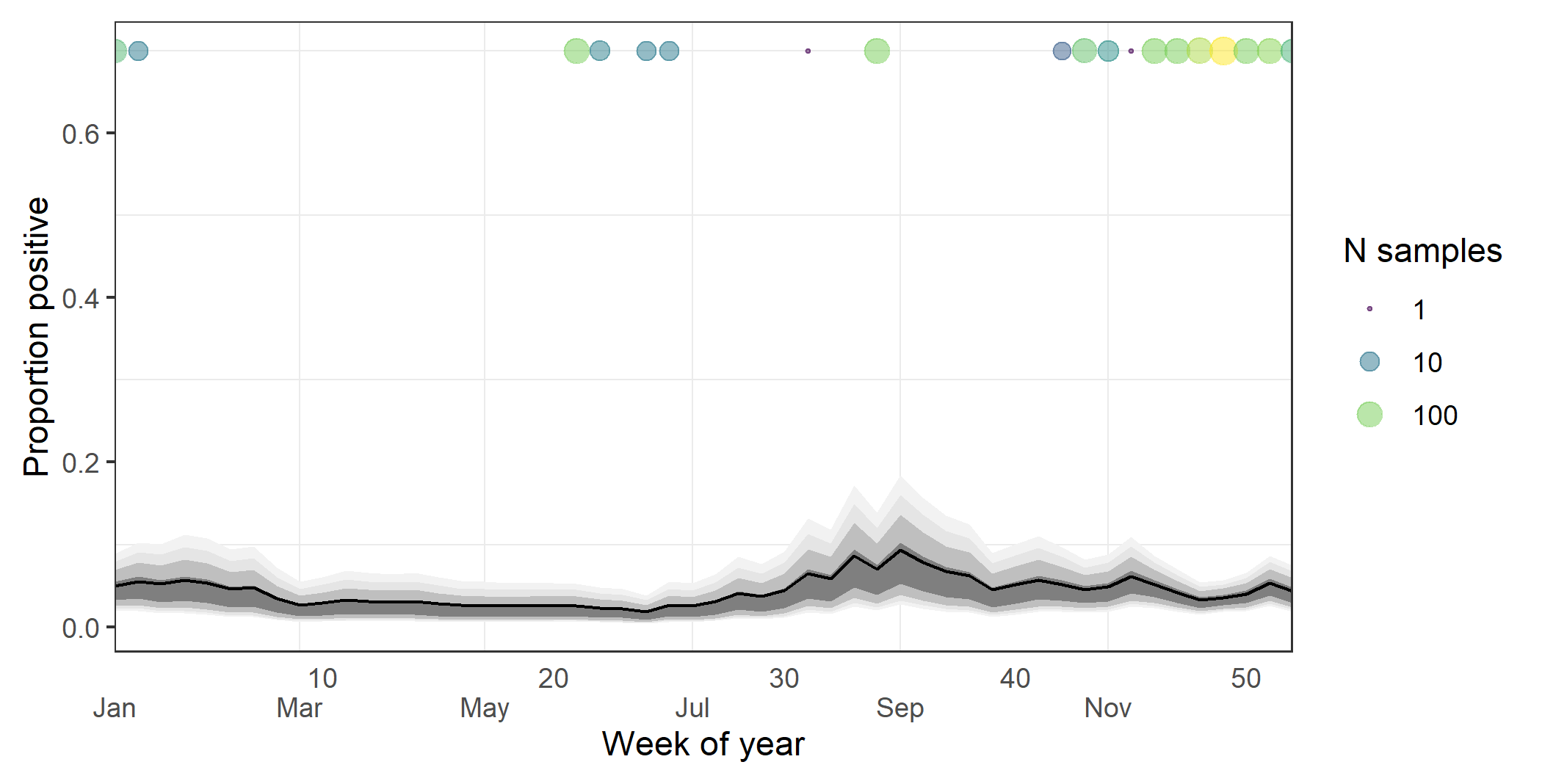

Supplement: Supplementary file 3 — Supplementary Information 3. [file 41598_2022_17396_MOESM3_ESM.zip › SupplementaryMaterials3/Common Eider.png]

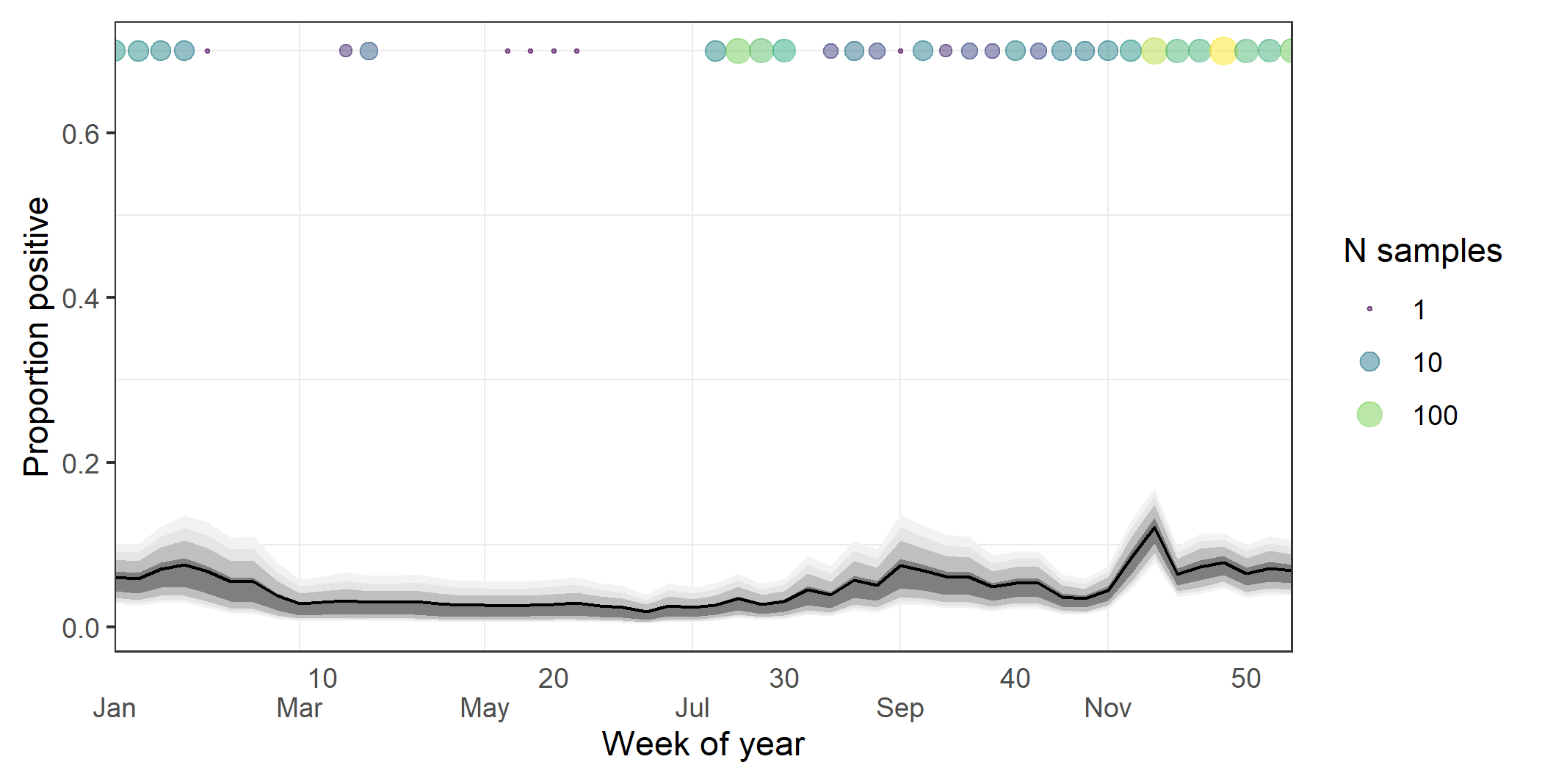

Supplement: Supplementary file 3 — Supplementary Information 3. [file 41598_2022_17396_MOESM3_ESM.zip › SupplementaryMaterials3/Common Goldeneye.png]

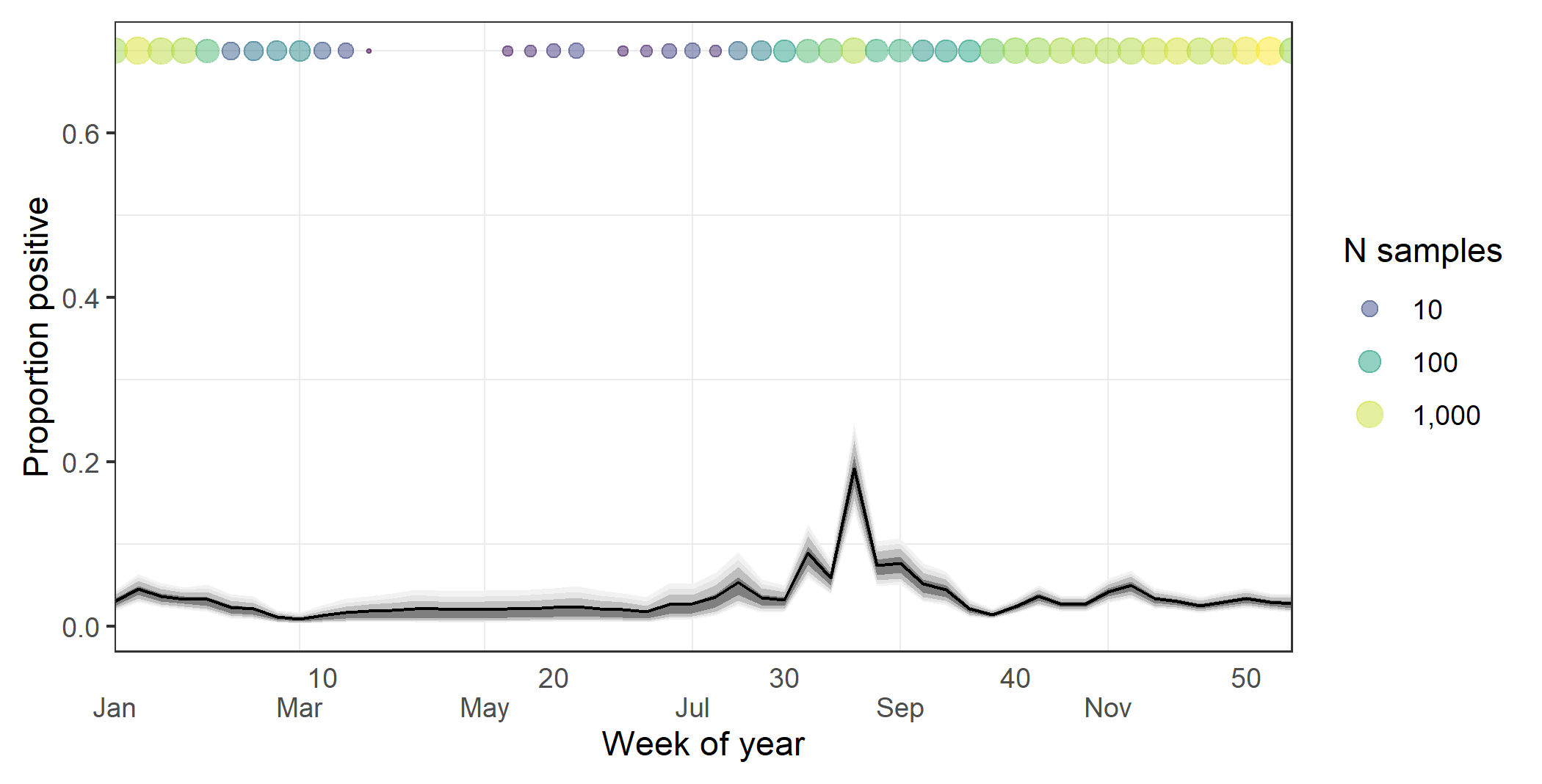

Supplement: Supplementary file 3 — Supplementary Information 3. [file 41598_2022_17396_MOESM3_ESM.zip › SupplementaryMaterials3/Gadwall.png]

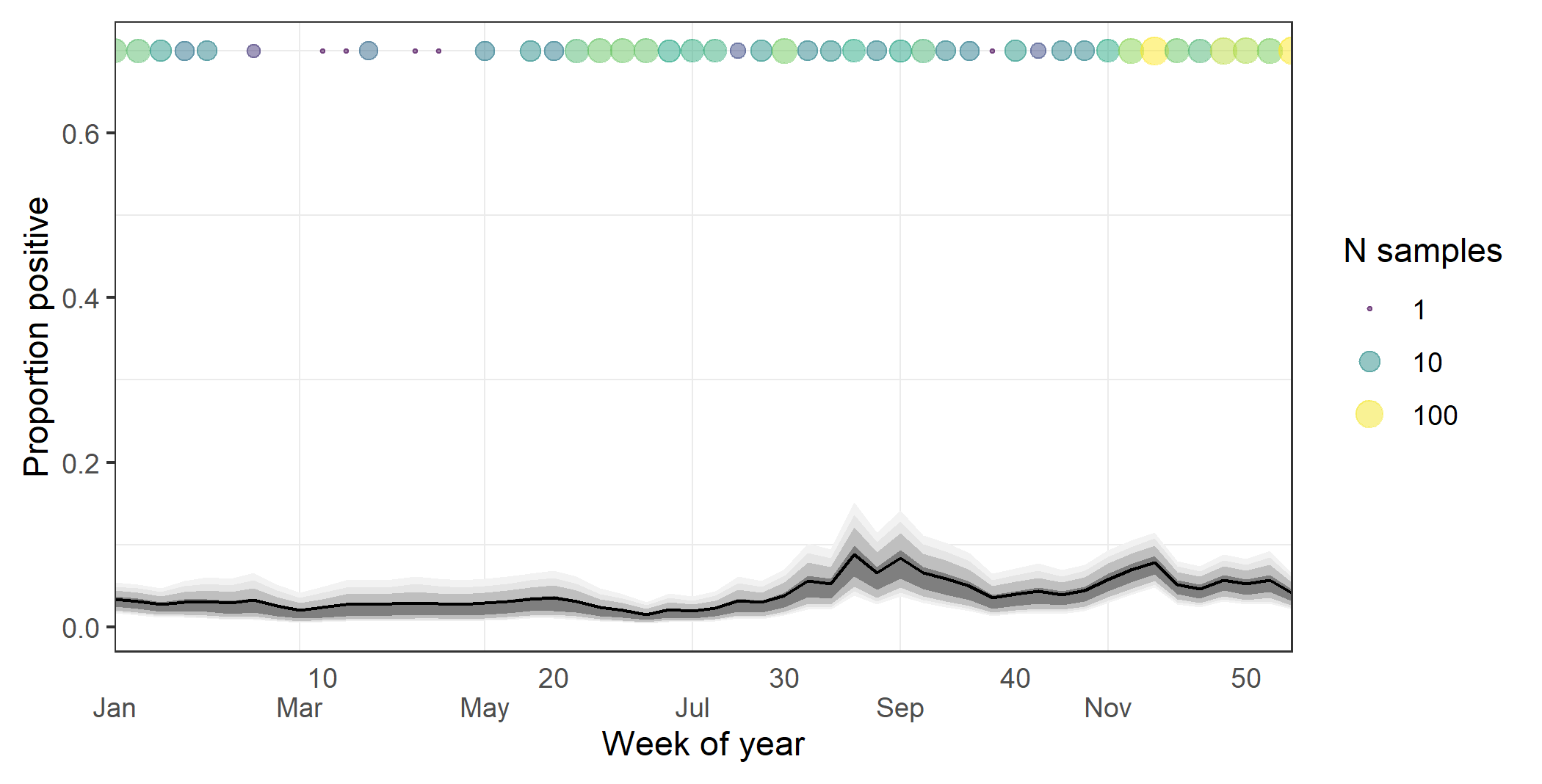

Supplement: Supplementary file 3 — Supplementary Information 3. [file 41598_2022_17396_MOESM3_ESM.zip › SupplementaryMaterials3/Greater Scaup.png]

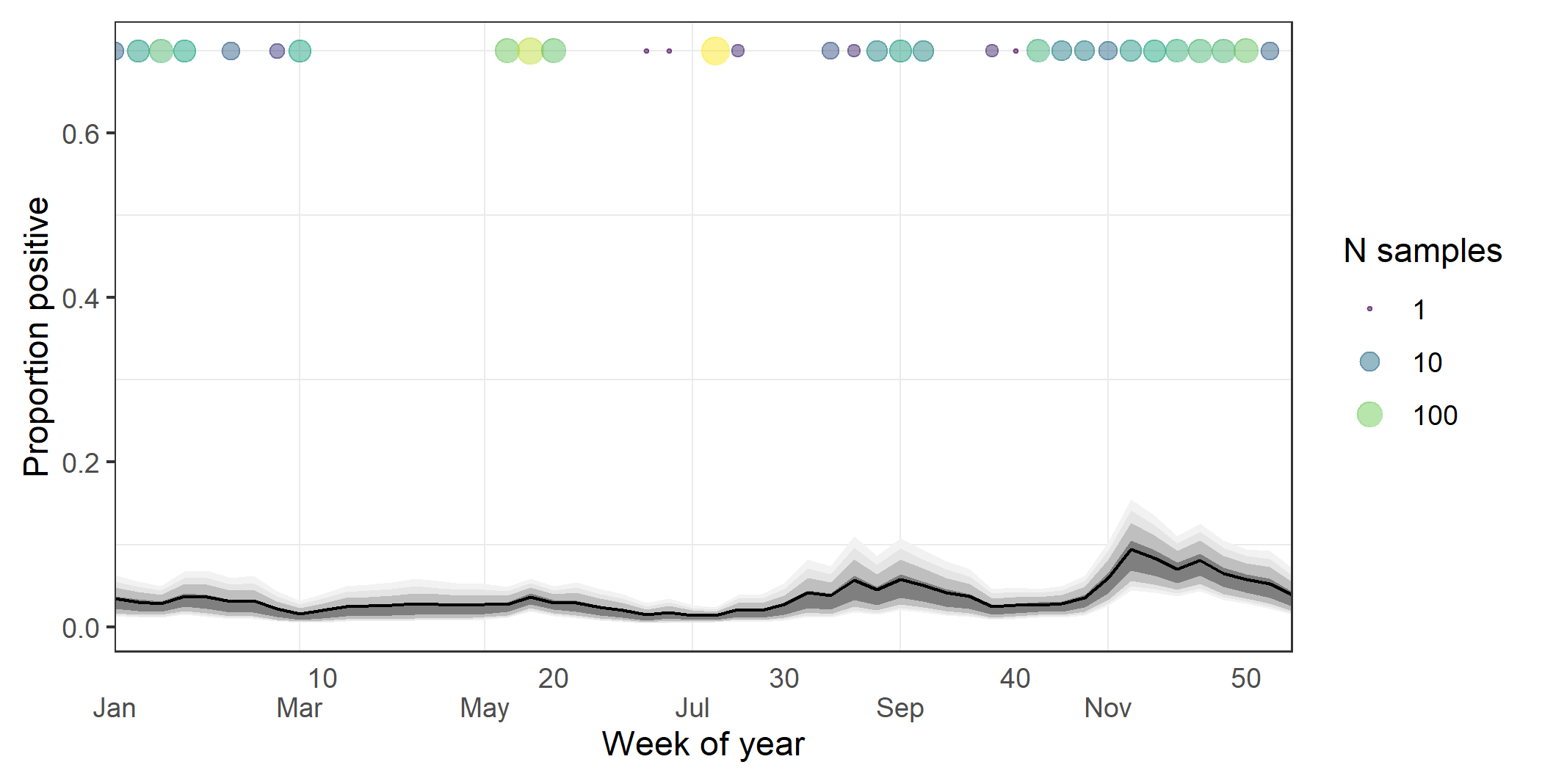

Supplement: Supplementary file 3 — Supplementary Information 3. [file 41598_2022_17396_MOESM3_ESM.zip › SupplementaryMaterials3/Greater White-fronted Goose.png]

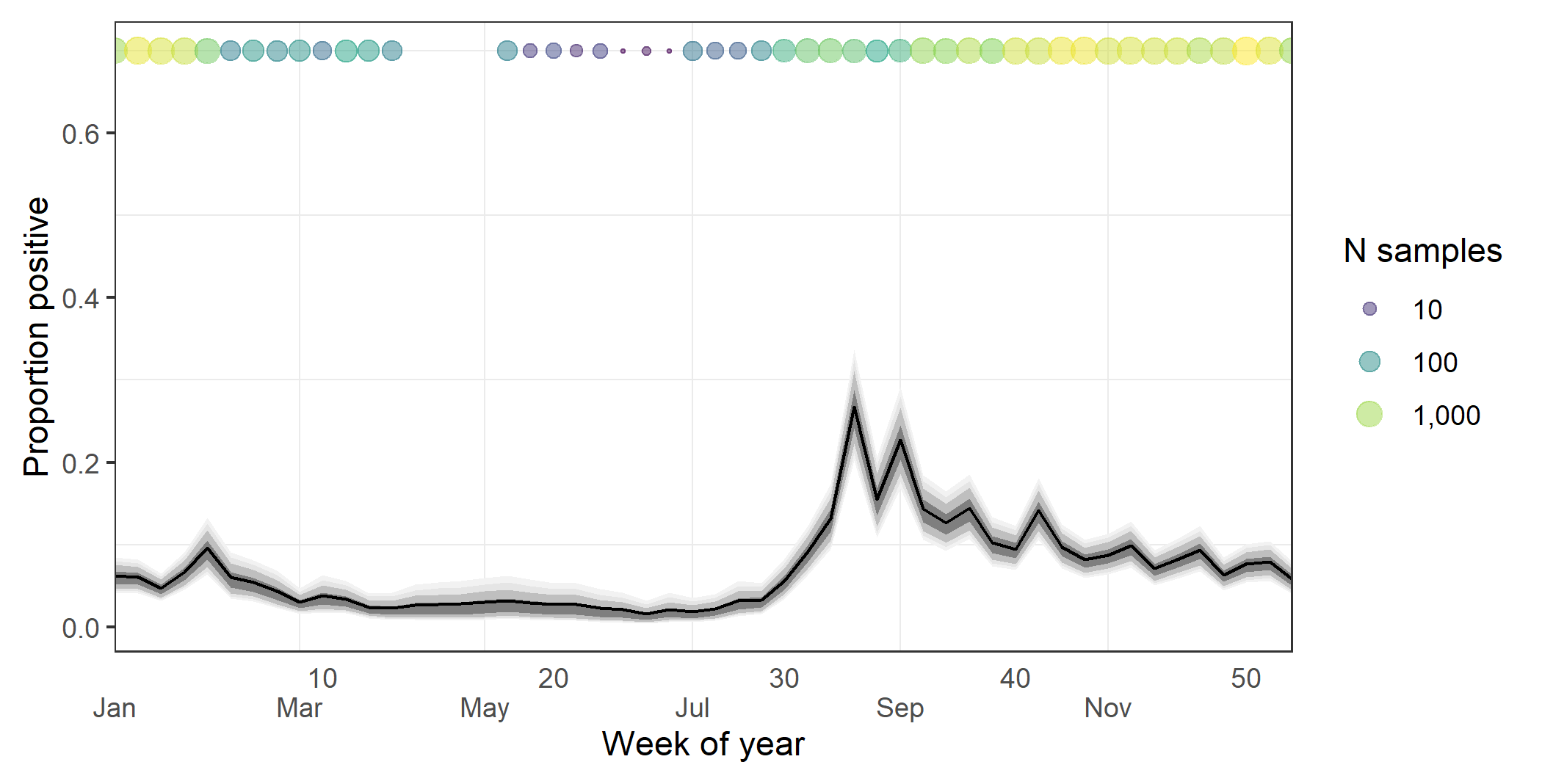

Supplement: Supplementary file 3 — Supplementary Information 3. [file 41598_2022_17396_MOESM3_ESM.zip › SupplementaryMaterials3/Green-winged Teal.png]

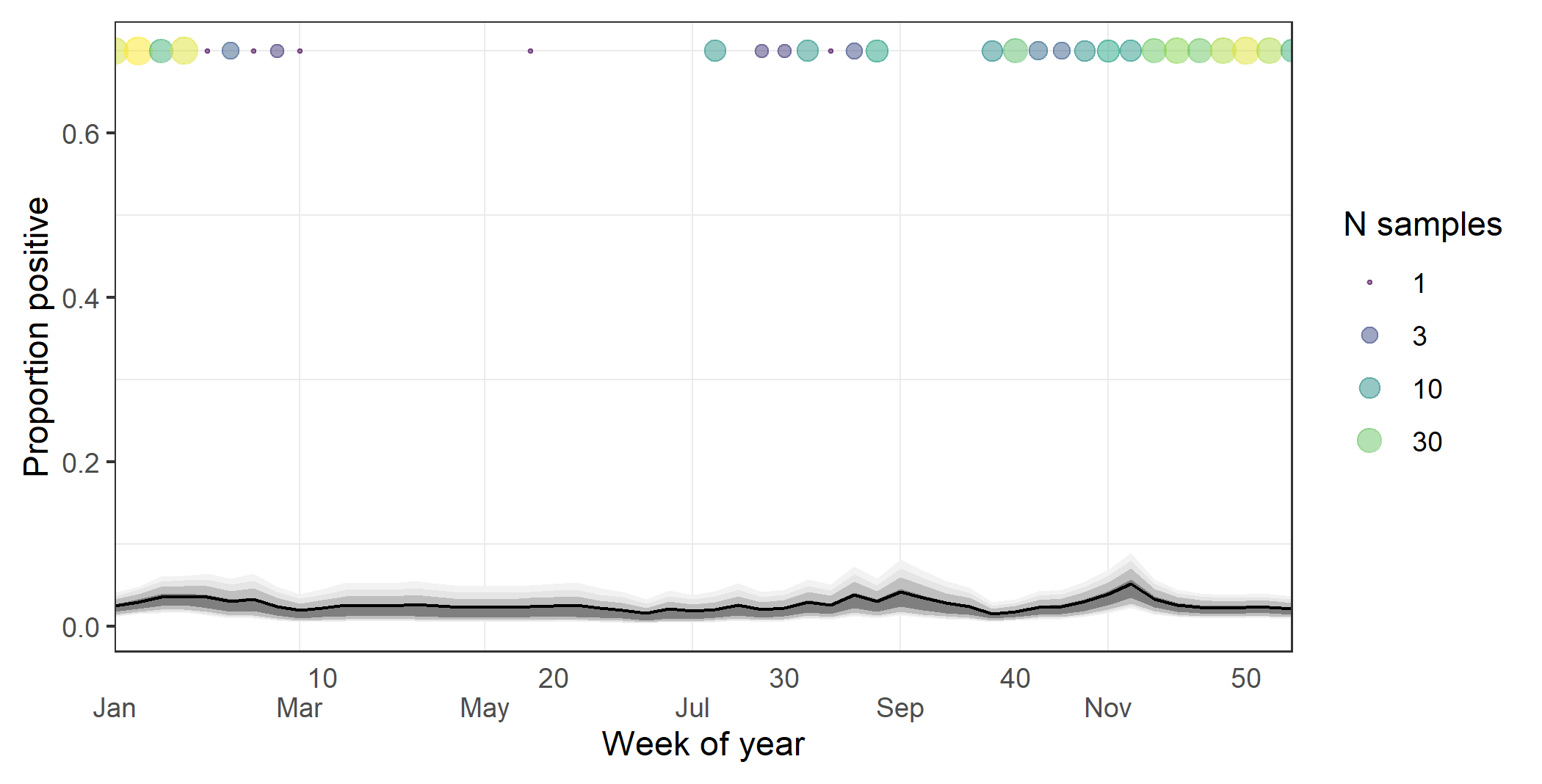

Supplement: Supplementary file 3 — Supplementary Information 3. [file 41598_2022_17396_MOESM3_ESM.zip › SupplementaryMaterials3/Hooded Merganser.png]

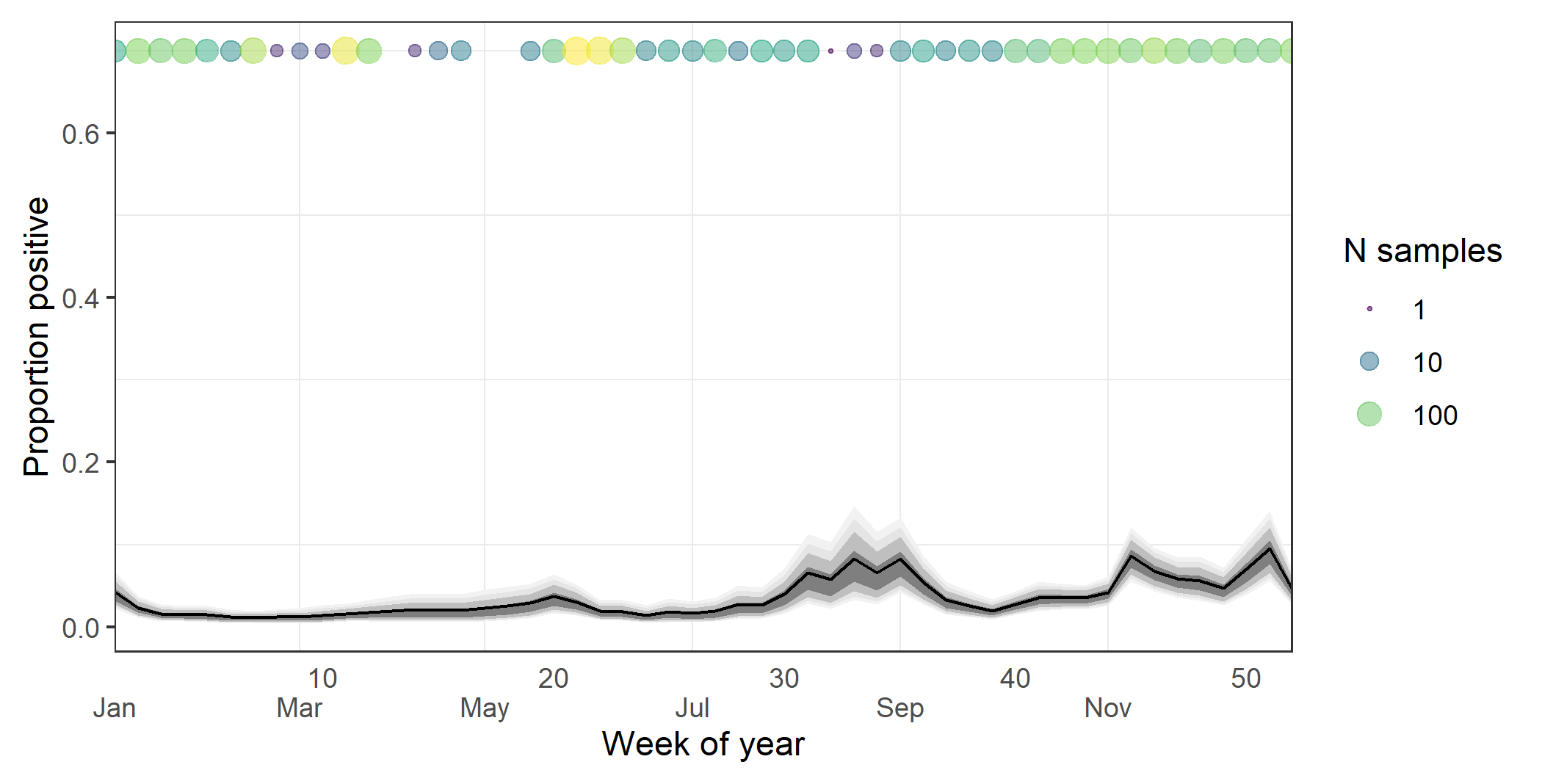

Supplement: Supplementary file 3 — Supplementary Information 3. [file 41598_2022_17396_MOESM3_ESM.zip › SupplementaryMaterials3/Lesser Scaup.png]

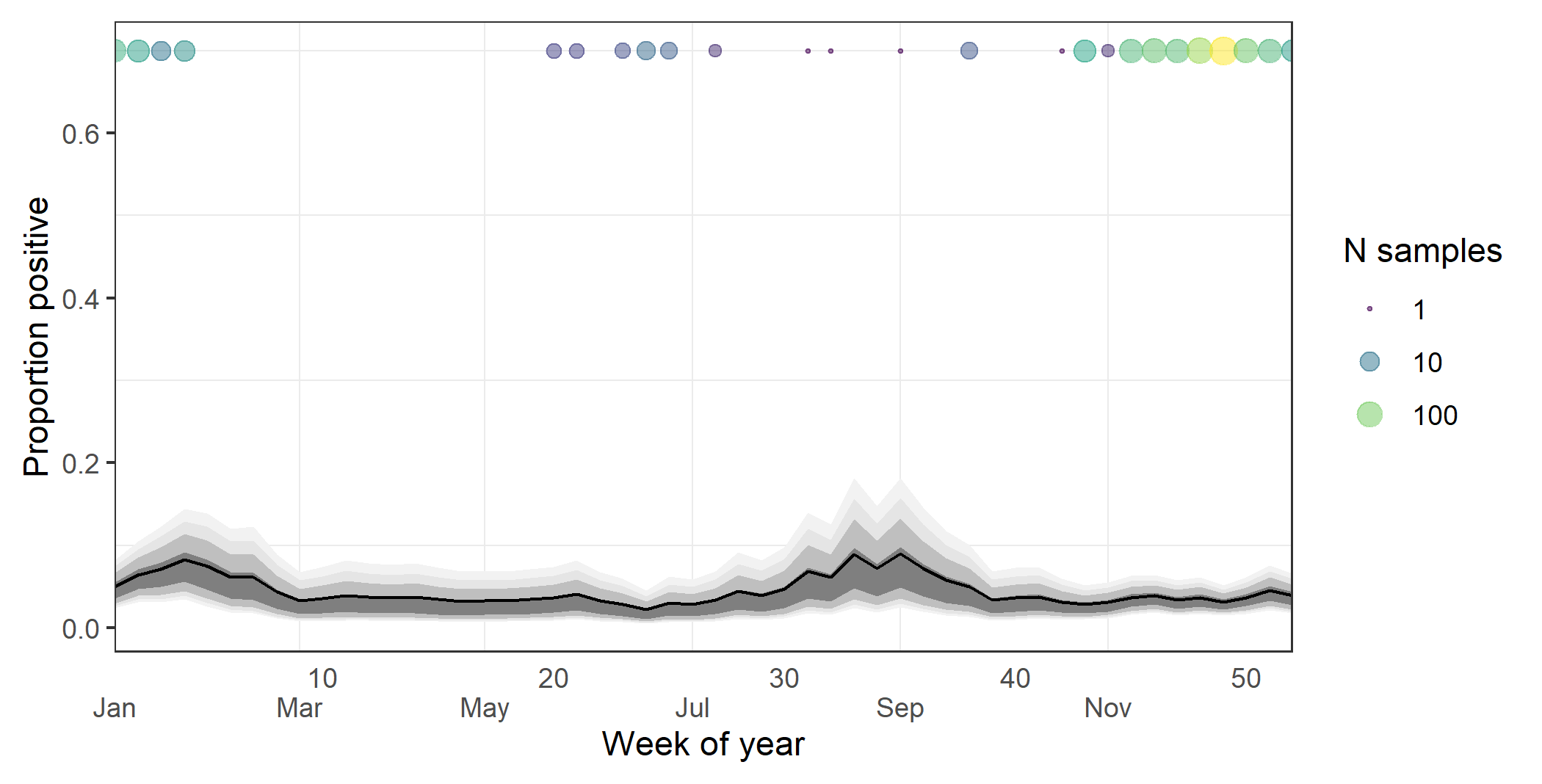

Supplement: Supplementary file 3 — Supplementary Information 3. [file 41598_2022_17396_MOESM3_ESM.zip › SupplementaryMaterials3/Long-tailed Duck.png]

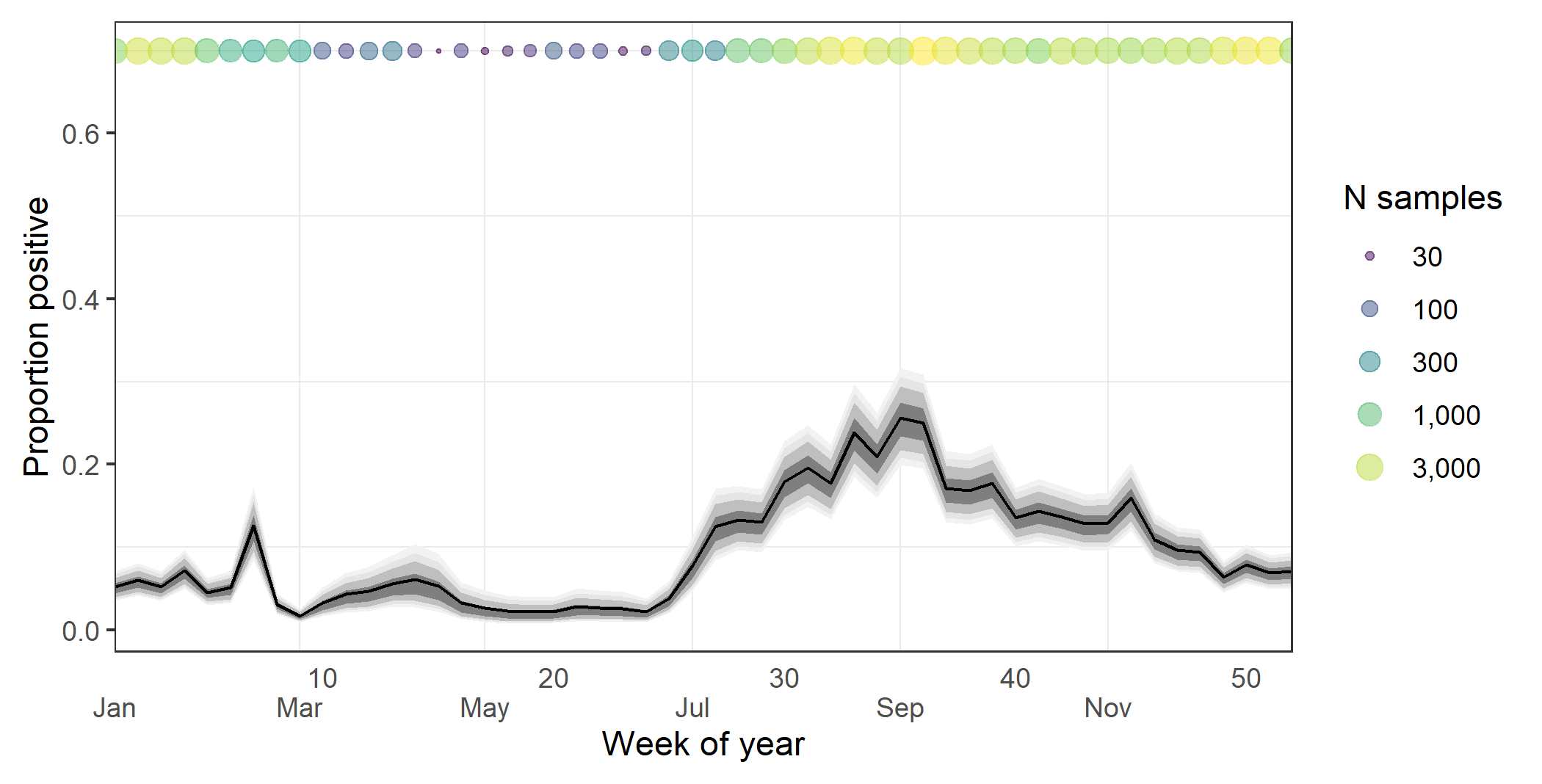

Supplement: Supplementary file 3 — Supplementary Information 3. [file 41598_2022_17396_MOESM3_ESM.zip › SupplementaryMaterials3/Mallard.png]

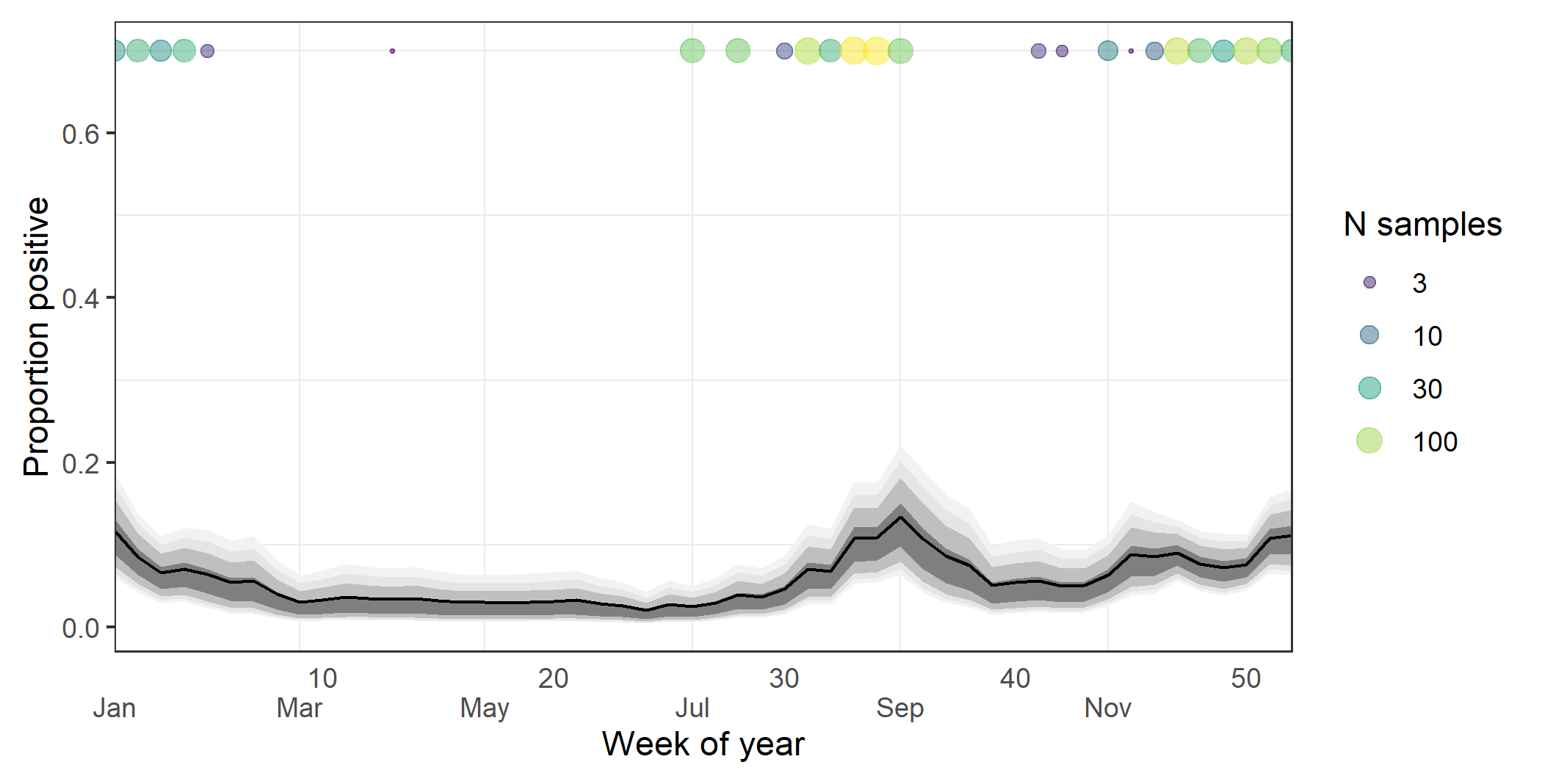

Supplement: Supplementary file 3 — Supplementary Information 3. [file 41598_2022_17396_MOESM3_ESM.zip › SupplementaryMaterials3/Mottled Duck.png]

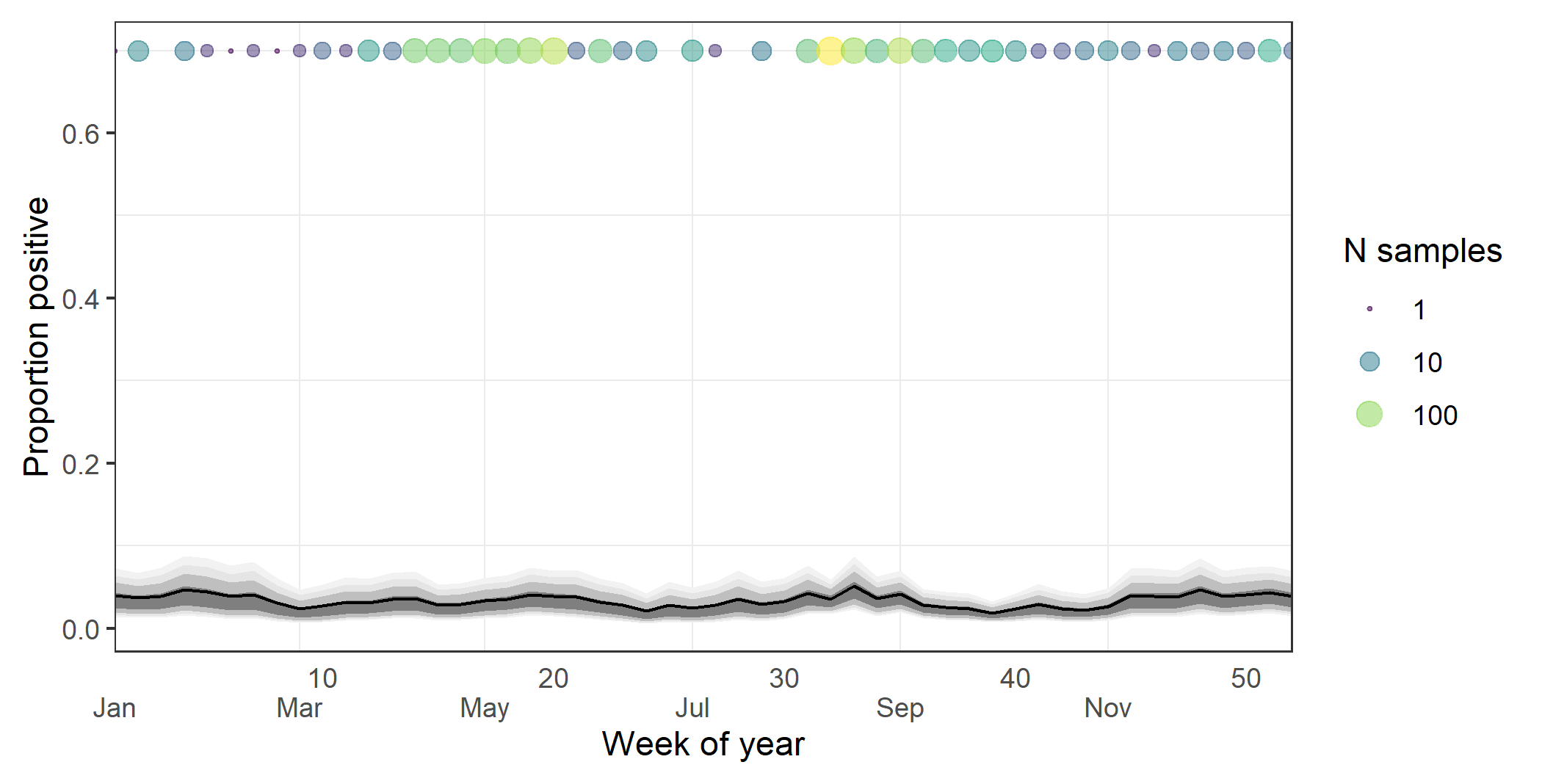

Supplement: Supplementary file 3 — Supplementary Information 3. [file 41598_2022_17396_MOESM3_ESM.zip › SupplementaryMaterials3/Mute Swan.png]

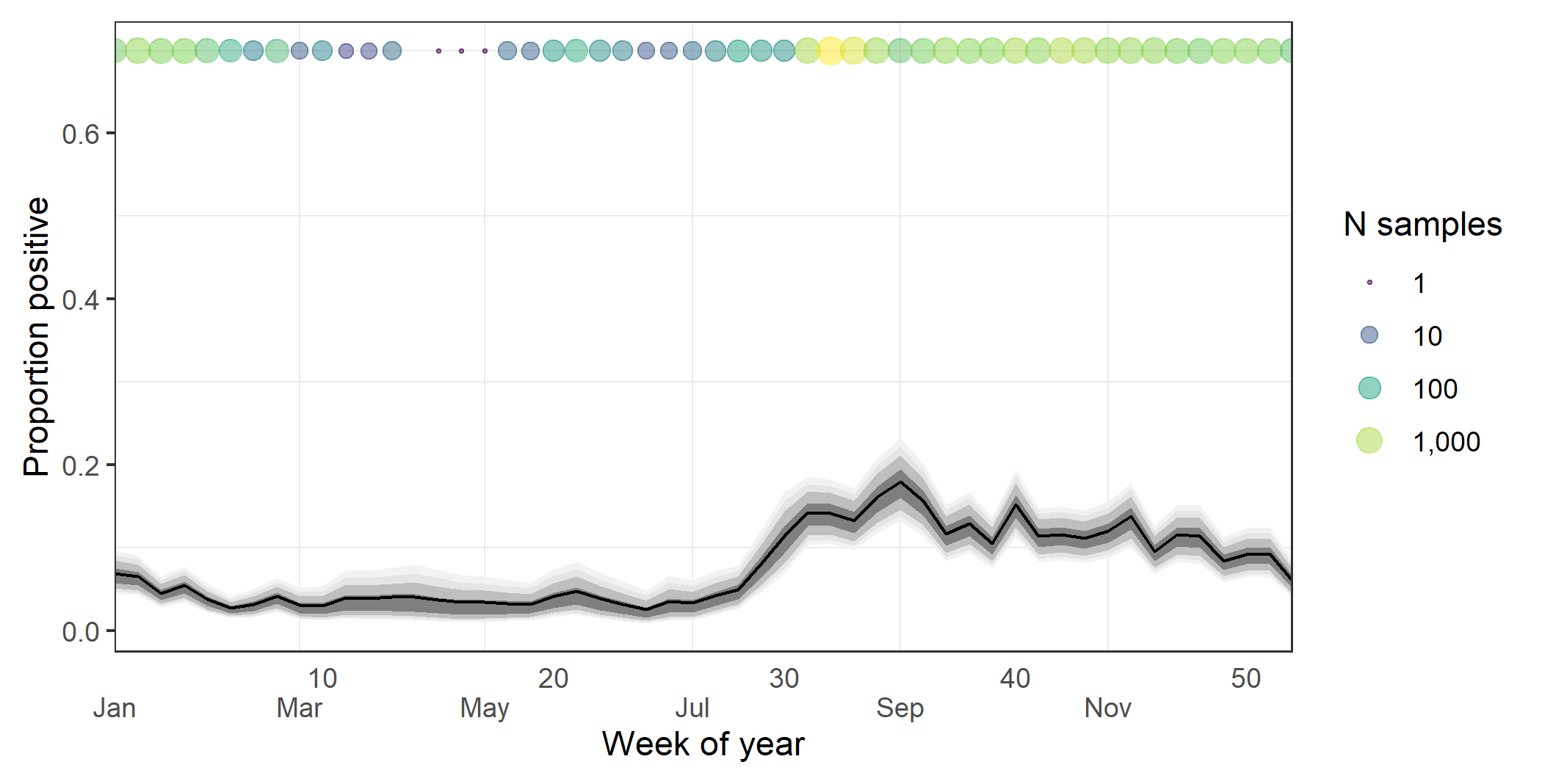

Supplement: Supplementary file 3 — Supplementary Information 3. [file 41598_2022_17396_MOESM3_ESM.zip › SupplementaryMaterials3/Northern Pintail.png]

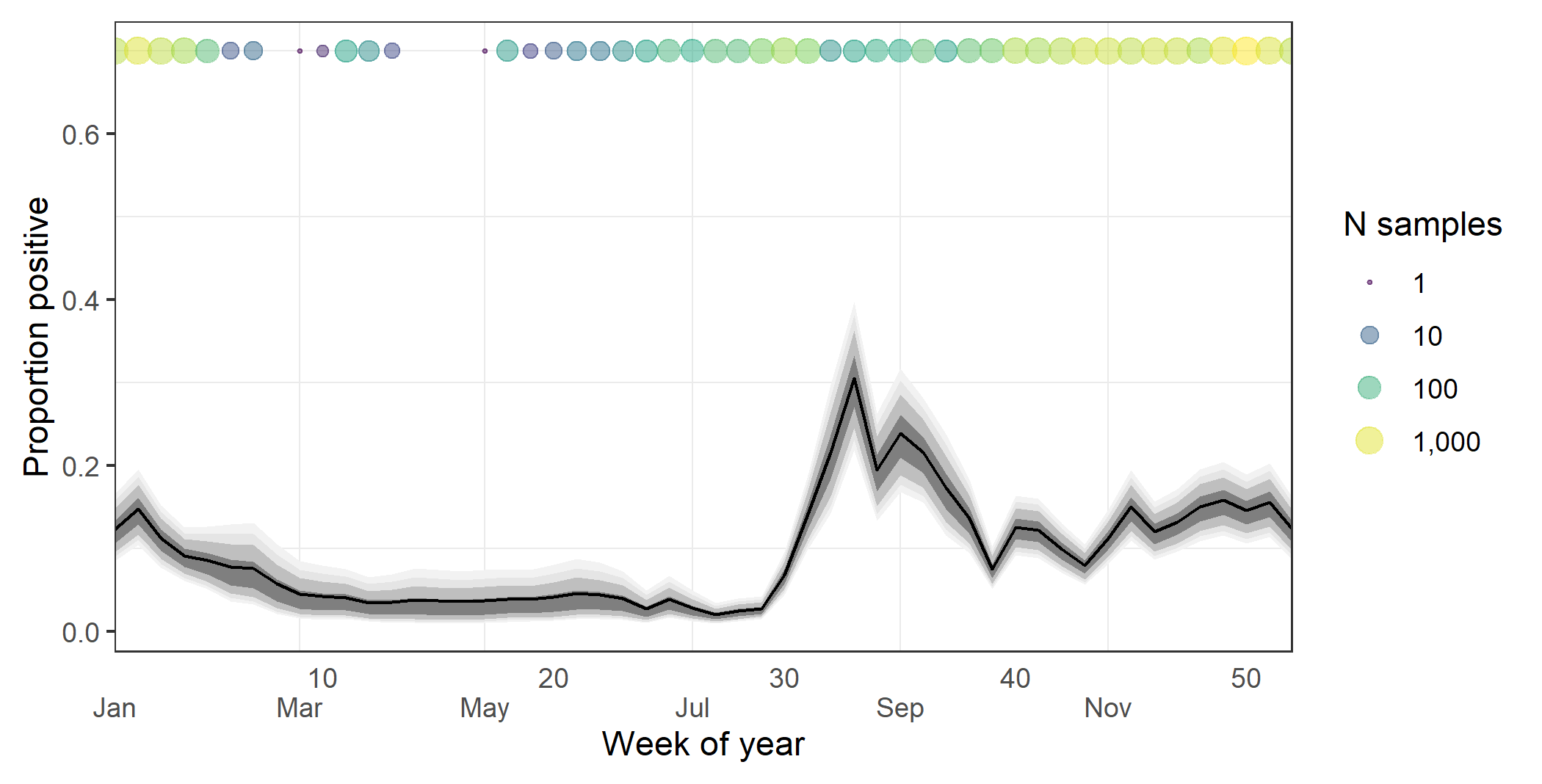

Supplement: Supplementary file 3 — Supplementary Information 3. [file 41598_2022_17396_MOESM3_ESM.zip › SupplementaryMaterials3/Northern Shoveler.png]

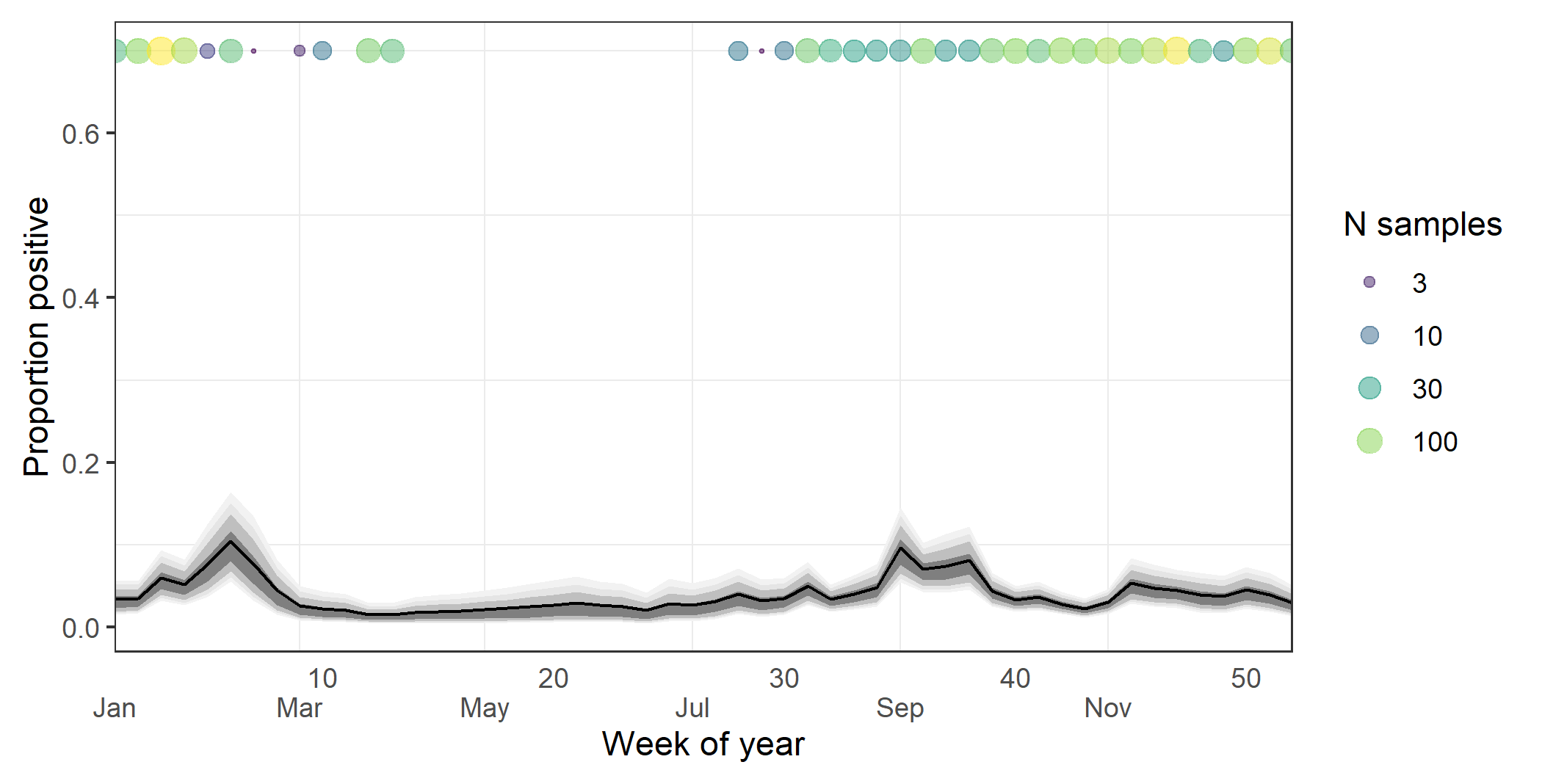

Supplement: Supplementary file 3 — Supplementary Information 3. [file 41598_2022_17396_MOESM3_ESM.zip › SupplementaryMaterials3/Redhead.png]

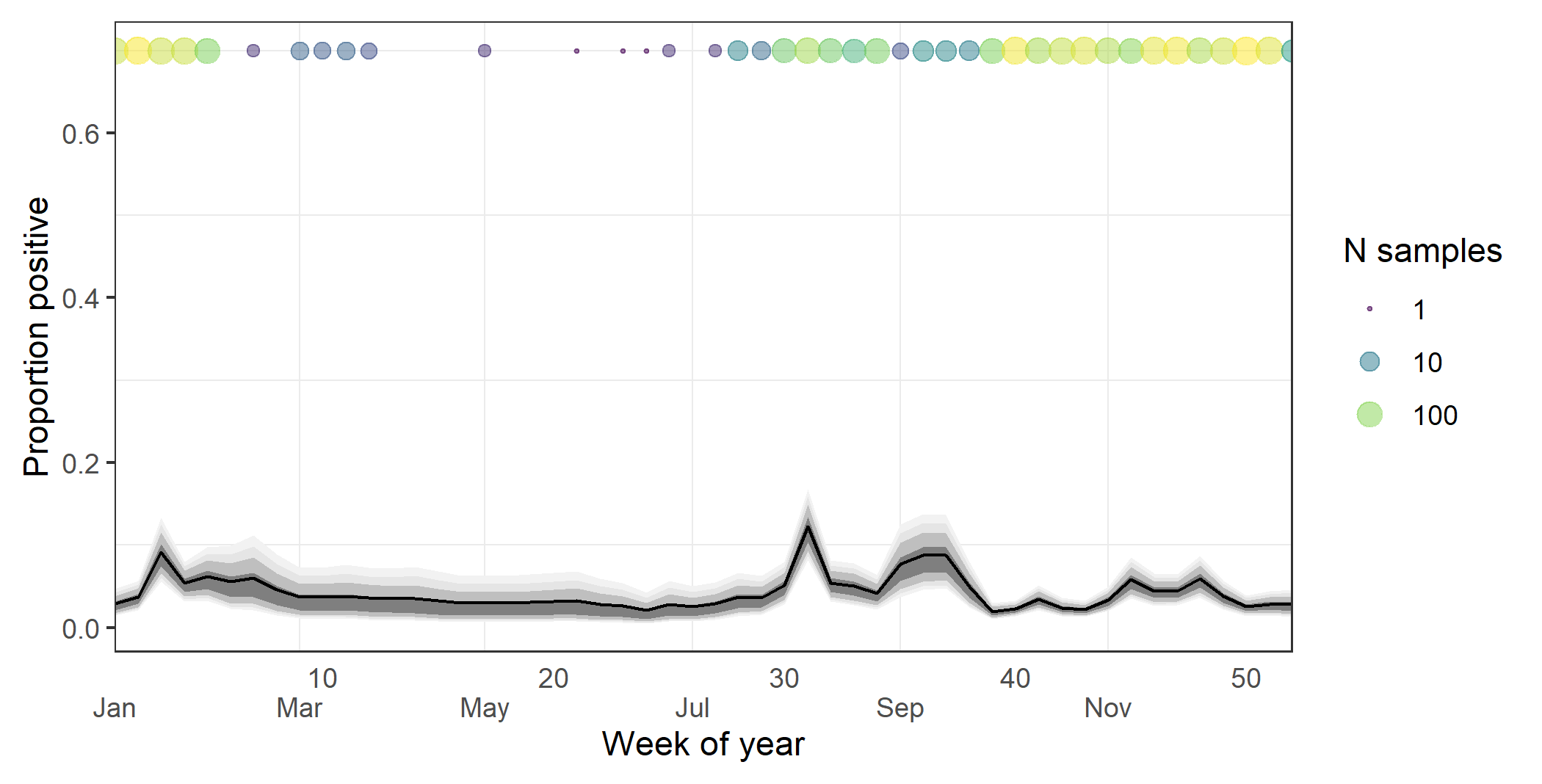

Supplement: Supplementary file 3 — Supplementary Information 3. [file 41598_2022_17396_MOESM3_ESM.zip › SupplementaryMaterials3/Ring-necked Duck.png]

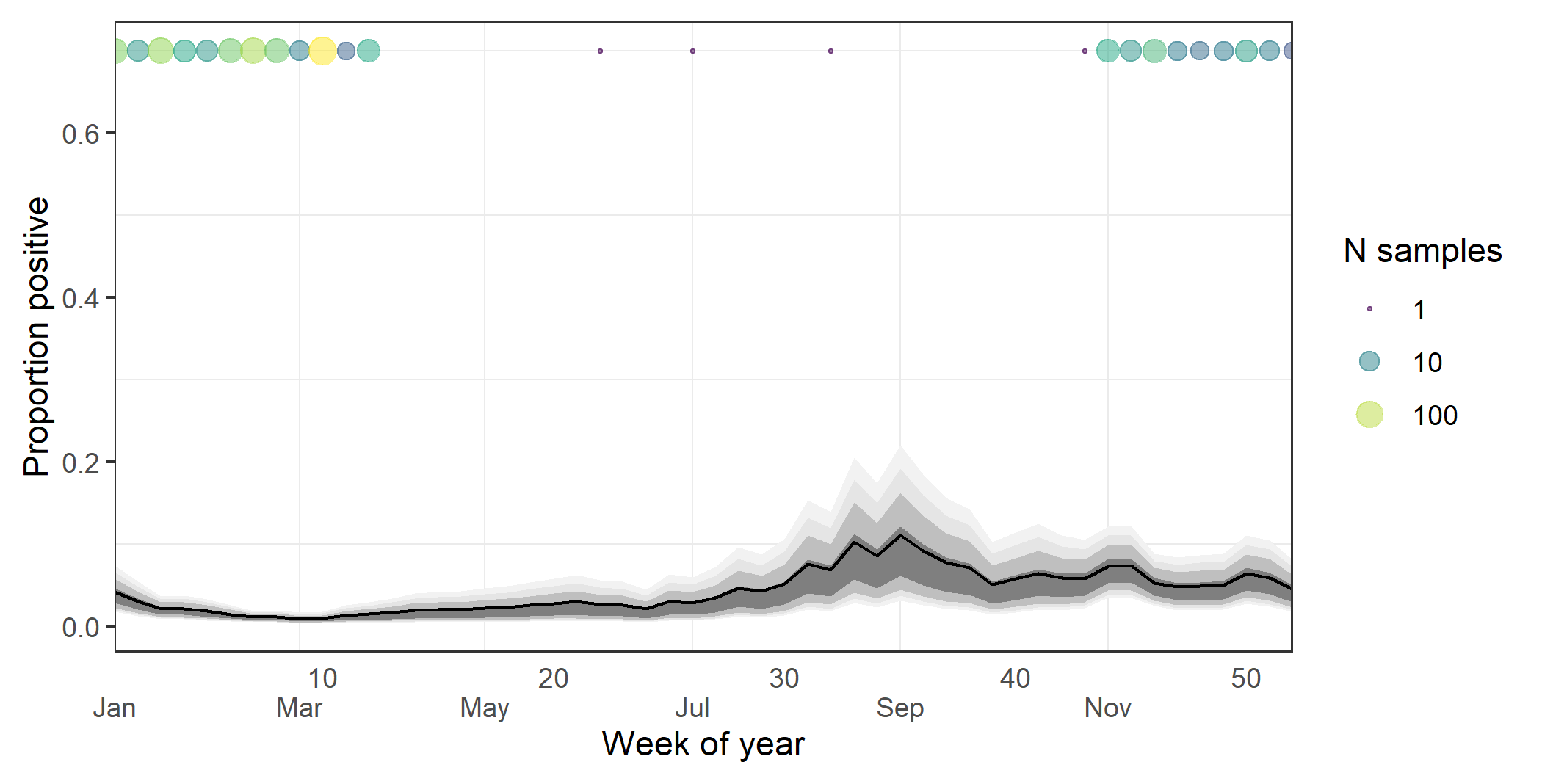

Supplement: Supplementary file 3 — Supplementary Information 3. [file 41598_2022_17396_MOESM3_ESM.zip › SupplementaryMaterials3/Ross's Goose.png]

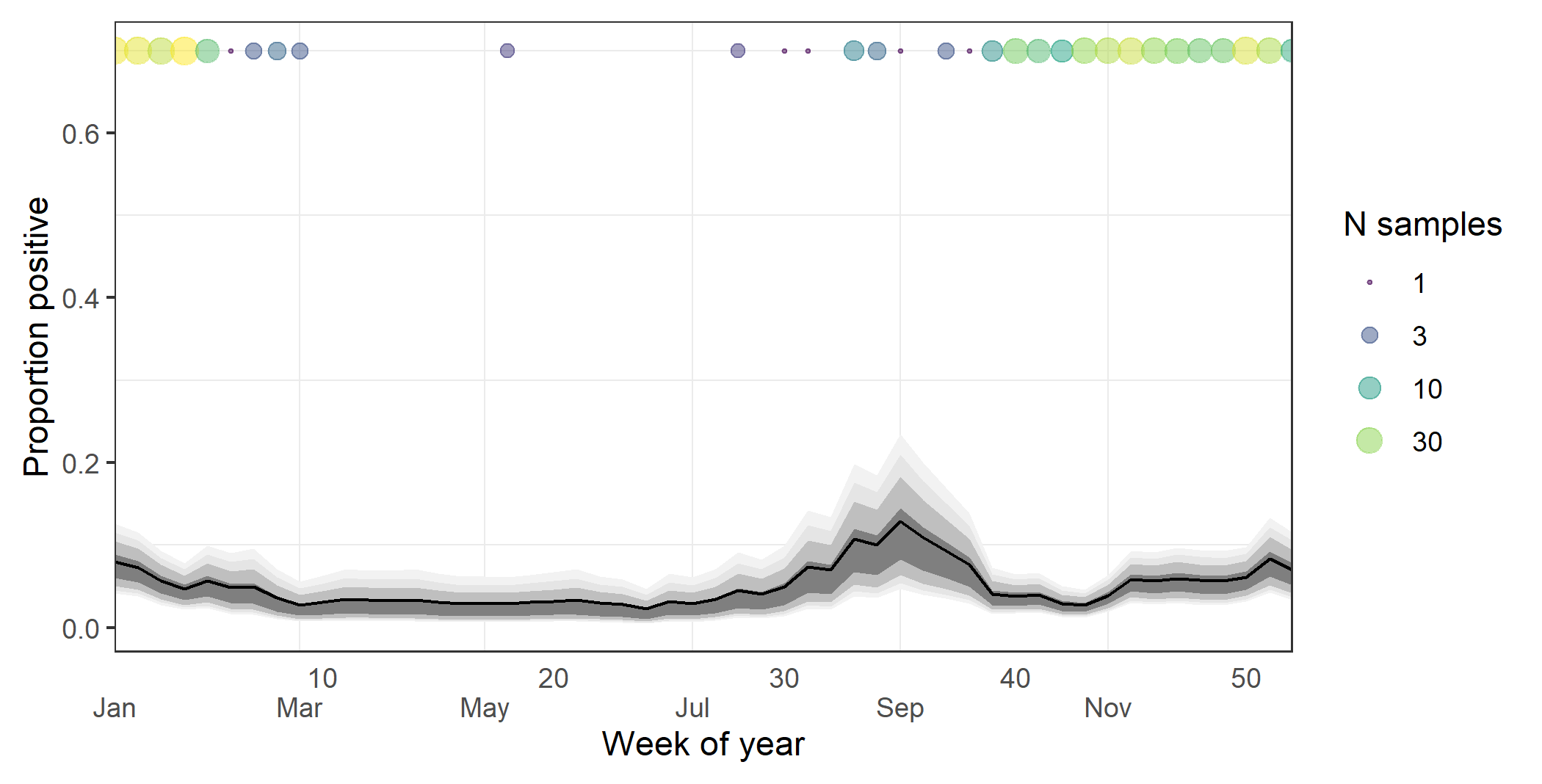

Supplement: Supplementary file 3 — Supplementary Information 3. [file 41598_2022_17396_MOESM3_ESM.zip › SupplementaryMaterials3/Ruddy Duck.png]

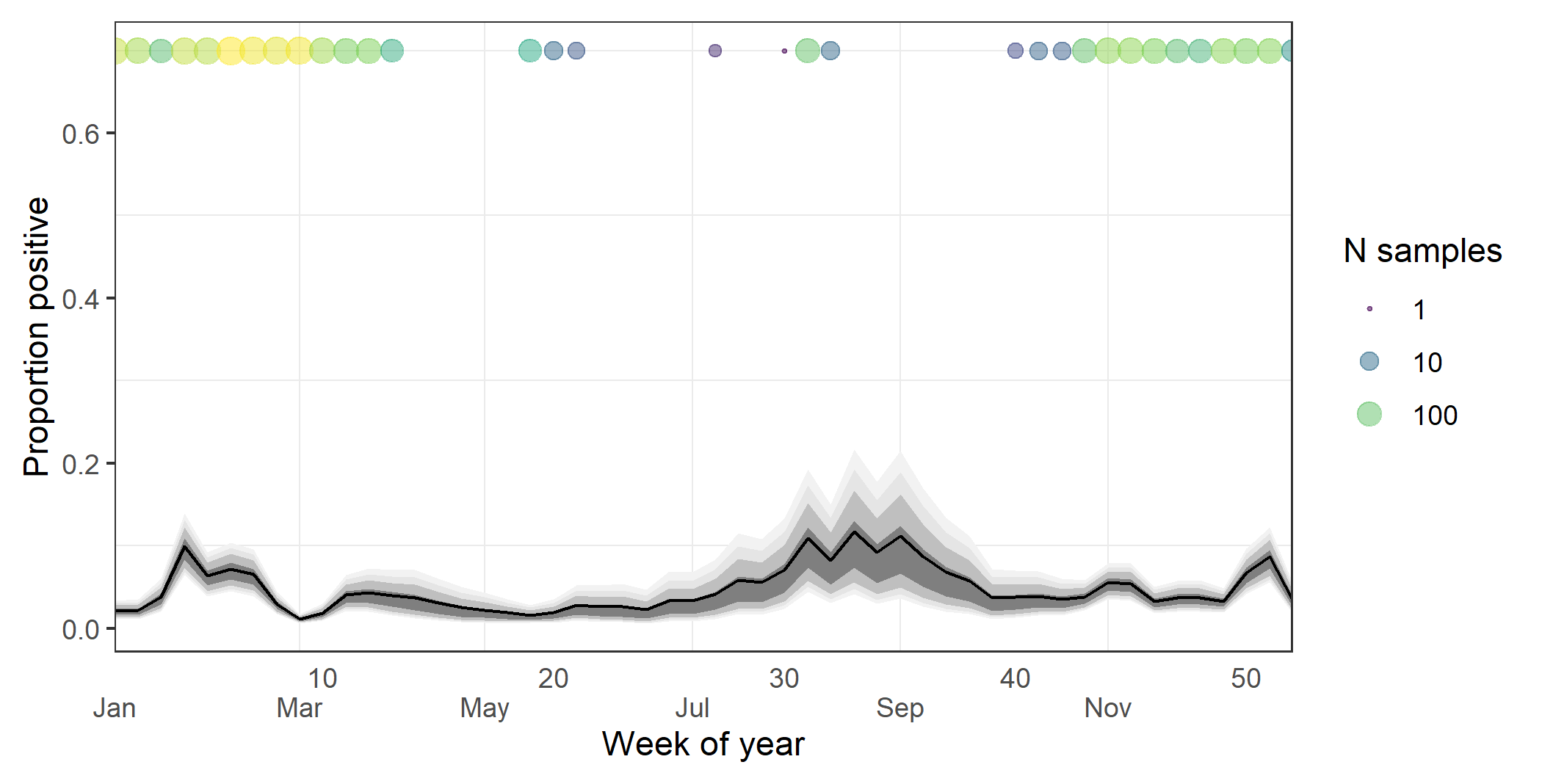

Supplement: Supplementary file 3 — Supplementary Information 3. [file 41598_2022_17396_MOESM3_ESM.zip › SupplementaryMaterials3/Snow Goose.png]

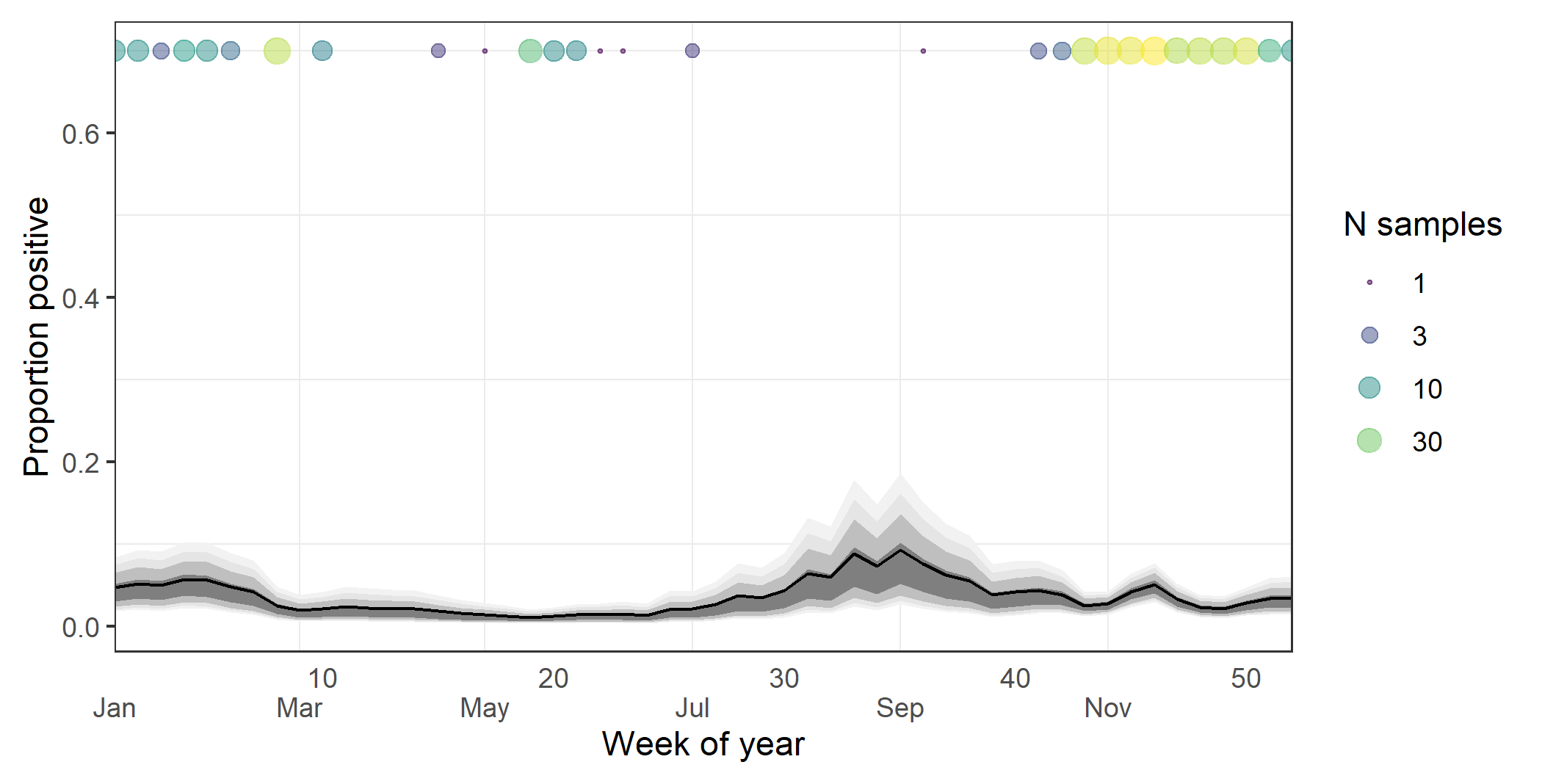

Supplement: Supplementary file 3 — Supplementary Information 3. [file 41598_2022_17396_MOESM3_ESM.zip › SupplementaryMaterials3/Tundra Swan.png]

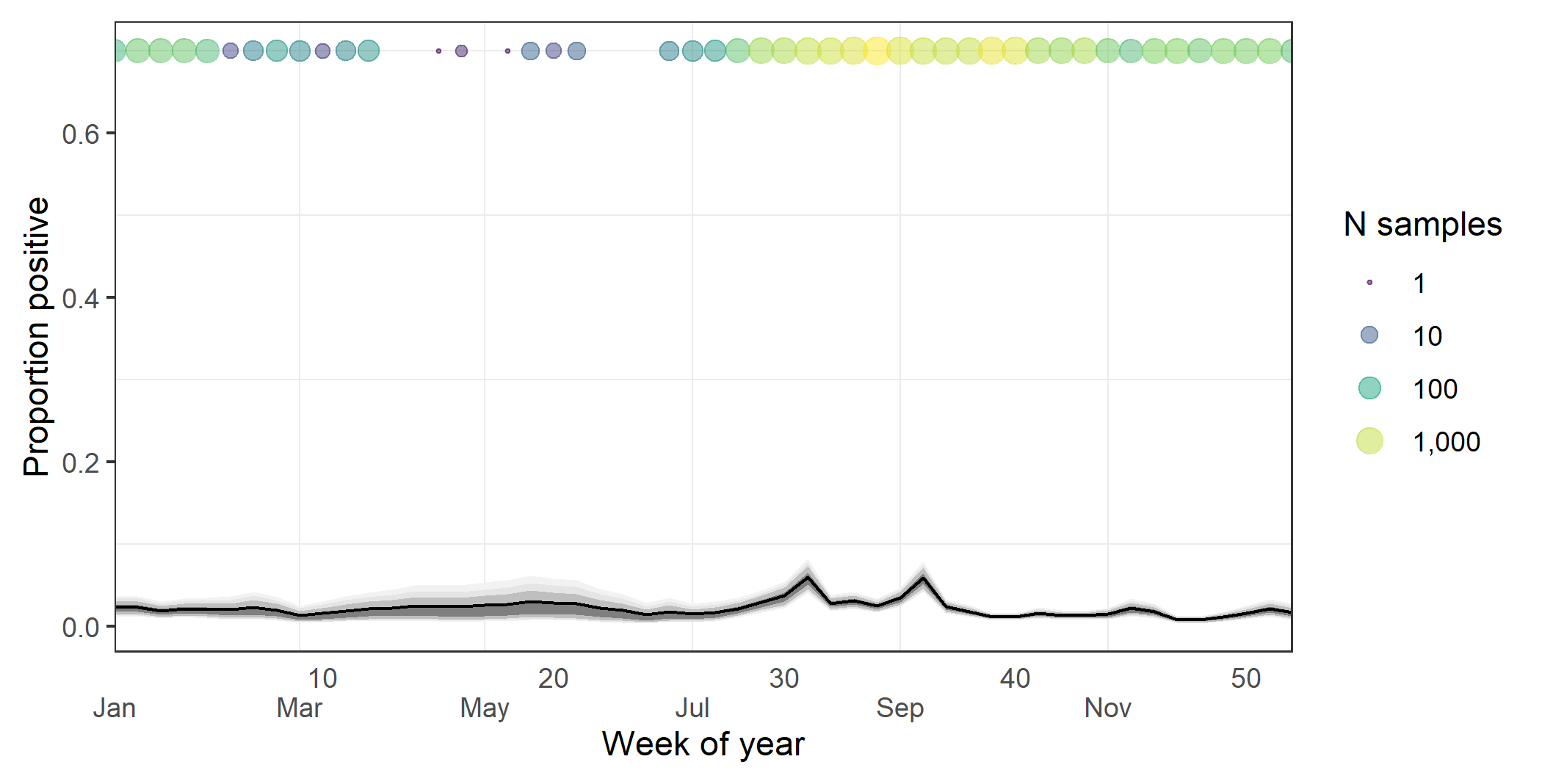

Supplement: Supplementary file 3 — Supplementary Information 3. [file 41598_2022_17396_MOESM3_ESM.zip › SupplementaryMaterials3/Wood Duck.png]
