# Supplementary material for: Spatiotemporal changes in influenza A virus prevalence among wild waterfowl inhabiting the continental United States throughout the annual cycle
Source: Sci Rep. 2022 Jul 29;12:13083. doi: 10.1038/s41598-022-17396-5 (PMC9338306; doi:10.1038/s41598-022-17396-5)
Supplement: Supplementary file 4 — Supplementary Information 4. [file 41598_2022_17396_MOESM4_ESM.docx]

Supplementary File Legends

Supplementary Materials 1. Supplementary figures.

Supplementary Material 2. Predicted proportion of birds testing positive for IAV for each species at weekly intervals for each county centroid in the continental United States. It should be noted that as all species shared a common spatial field, we did not choose to mask the geographic regions outside of species’ ranges, and thus predictions do exist for locations beyond a species’ natural range.

Supplementary Material 3. Percentage of individuals predicted to test positive for IAV (± 50, 80, 90, and 95% CI) for each species for each week. Predictions are based on the overall prevalence values in the USDA dataset, which had a higher detection rate, and ignore the spatial component. Circles running along the x-axis indicate the number of samples for each species taken during that week. Estimates for time periods without samples for a given species should be taken with caution as predictions are primarily based on the among-species correlation.
